# Mechanisms Underlying the Exquisite Sensitivity of *Candida albicans* to Combinatorial Cationic and Oxidative Stress That Enhances the Potent Fungicidal Activity of Phagocytes

**DOI:** 10.1128/mBio.01334-14

**Published:** 2014-07-15

**Authors:** Despoina Kaloriti, Mette Jacobsen, Zhikang Yin, Miranda Patterson, Anna Tillmann, Deborah A. Smith, Emily Cook, Tao You, Melissa J. Grimm, Iryna Bohovych, Celso Grebogi, Brahm H. Segal, Neil A. R. Gow, Ken Haynes, Janet Quinn, Alistair J. P. Brown

**Affiliations:** ^a^ School of Medical Sciences, University of Aberdeen, Foresterhill, Aberdeen, United Kingdom; ^b^ Institute for Cell and Molecular Biosciences, Newcastle University, Newcastle upon Tyne, United Kingdom; ^c^ Department of Biosciences, College of Life and Environmental Sciences, University of Exeter, Exeter, United Kingdom; ^d^ Institute for Complex Systems and Mathematical Biology, King’s College, University of Aberdeen, Old Aberdeen, Aberdeen, United Kingdom; ^e^ Department of Medicine, Roswell Park Cancer Institute, Buffalo, New York, USA; ^f^ Department of Immunology, Roswell Park Cancer Institute, Buffalo, New York, USA; ^g^ Department of Medicine, School of Medicine and Biomedical Sciences, University at Buffalo, Buffalo, New York, USA

## Abstract

Immune cells exploit reactive oxygen species (ROS) and cationic fluxes to kill microbial pathogens, such as the fungus *Candida albicans*. Yet, *C. albicans* is resistant to these stresses *in vitro.* Therefore, what accounts for the potent antifungal activity of neutrophils? We show that simultaneous exposure to oxidative and cationic stresses is much more potent than the individual stresses themselves and that this combinatorial stress kills *C. albicans* synergistically *in vitro*. We also show that the high fungicidal activity of human neutrophils is dependent on the combinatorial effects of the oxidative burst and cationic fluxes, as their pharmacological attenuation with apocynin or glibenclamide reduced phagocytic potency to a similar extent. The mechanistic basis for the extreme potency of combinatorial cationic plus oxidative stress—a phenomenon we term stress pathway interference—lies with the inhibition of hydrogen peroxide detoxification by the cations. In *C. albicans* this causes the intracellular accumulation of ROS, the inhibition of Cap1 (a transcriptional activator that normally drives the transcriptional response to oxidative stress), and altered readouts of the stress-activated protein kinase Hog1. This leads to a loss of oxidative and cationic stress transcriptional outputs, a precipitous collapse in stress adaptation, and cell death. This stress pathway interference can be suppressed by ectopic catalase (Cat1) expression, which inhibits the intracellular accumulation of ROS and the synergistic killing of *C. albicans* cells by combinatorial cationic plus oxidative stress. Stress pathway interference represents a powerful fungicidal mechanism employed by the host that suggests novel approaches to potentiate antifungal therapy.

## IMPORTANCE

The immune system combats infection via phagocytic cells that recognize and kill pathogenic microbes. Human neutrophils combat *Candida* infections by killing this fungus with a potent mix of chemicals that includes reactive oxygen species (ROS) and cations. Yet, *Candida albicans* is relatively resistant to these stresses *in vitro*. We show that it is the combination of oxidative plus cationic stresses that kills yeasts so effectively, and we define the molecular mechanisms that underlie this potency. Cations inhibit catalase. This leads to the accumulation of intracellular ROS and inhibits the transcription factor Cap1, which is critical for the oxidative stress response in *C. albicans*. This triggers a dramatic collapse in fungal stress adaptation and cell death. Blocking either the oxidative burst or cationic fluxes in human neutrophils significantly reduces their ability to kill this fungal pathogen, indicating that combinatorial stress is pivotal to immune surveillance.

## INTRODUCTION

*Candida albicans* is a major opportunistic fungal pathogen of humans (1, 2). It exists as a relatively harmless commensal in the oral cavity, gastrointestinal tract, and urogenital tract of most healthy individuals. However, the fungus is a frequent cause of mucosal infections (thrush), and it can cause potentially fatal infections of the blood and internal organs when immunological defenses are severely compromised (3–6). For example, HIV patients are particularly prone to mucosal infections and neutropenic patients are prone to systemic candidiasis (1–3), although highly active antiretroviral therapy and prophylactic treatment with antifungal drugs, respectively, are reducing the impacts of these threats in these patient groups (7–9). These observations highlight the major importance of immunological defenses in preventing the transition from *C. albicans* colonization to infection (10–12).

Macrophages and neutrophils attempt to kill *C. albicans* cells by exposing them to a battery of toxic chemicals (11, 13, 14). Meanwhile, *C. albicans* attempts to respond and escape these fungicidal mechanisms by activating robust adaptive responses and by triggering morphogenesis (15–21). Phagocytes generally win this battle, killing the fungus and preventing infection in healthy individuals. In contrast, patients undergoing treatments that impose neutropenia or individuals with mutations that perturb the phagocytic oxidative burst display high susceptibilities to fungal infection (11, 22, 23). This highlights the critical importance of reactive oxygen species (ROS) for the fungicidal potency of phagocytes.

*C. albicans* responds to oxidative stress by activating evolutionarily conserved signaling pathways that drive the adaptive mechanisms which lead to the detoxification of the oxidative stress and the repair of damage caused by ROS. The transcriptional response to oxidative stress is largely mediated by the AP-1-like transcription factor Cap1, which is orthologous to *Saccharomyces cerevisiae* Yap1 and *Schizosaccharomyces pombe* Pap1 (19–21, 24, 25). Cap1 activates genes that encode key oxidative stress functions in *C. albicans*, such as catalase (*CAT1*), which detoxifies hydrogen peroxide (H_2_O_2_), as well as thioredoxin (*TRX1*) and thioredoxin reductase (*TRR1*), which contribute to the restoration of redox homeostasis (19, 25, 26).

The Hog1 stress-activated protein kinase, which is orthologous to *Saccharomyces cerevisiae* Hog1 and *Schizosaccharomyces pombe* Sty1, also contributes to oxidative stress resistance (19, 27, 28). However, the contribution of Hog1 to oxidative stress resistance appears to be mediated primarily at posttranscriptional levels (26, 29). In addition, Hog1 contributes to osmotic stress adaptation and resistance (26, 28, 30). The transcriptional outputs of Hog1 are environmentally contingent (26), but under the experimental conditions examined to date Hog1 plays a central role in the transcriptional response to osmotic and cationic stress (Na^+^ and K^+^), activating glycerol permease (*HGT10*) and glycerol biosynthetic genes (*GPD2* and *RHR2*) (26, 28, 30). This response promotes glycerol accumulation and osmo-adaptation.

There is a conundrum with regard to phagocytic potency. Phagocytes are thought to kill *C. albicans* by generating high concentrations of ROS, which are estimated to be equivalent to around 5 mM H_2_O_2_ (11, 13, 14, 17, 31, 32). Also, cation levels can reach over 500 mM in some microenvironments (33). However, *C. albicans* exhibits robust adaptation to these stresses, displaying resistance to high levels of H_2_O_2_ and NaCl *in vitro* (34–36). Therefore, additional factors must contribute to phagocytic potency. Most studies of stress adaptation have focused on responses to individual environmental insults (17–21, 24–30, 34–36). However, many microbes are subjected to combinations of stress in the complex and dynamic microenvironments they normally inhabit. For example, *C. albicans* is exposed simultaneously to combinations of ROS and cationic fluxes following phagocytosis by neutrophils (13, 32, 37). Therefore, we reasoned that the potency of neutrophils might be due to the synergistic combination of oxidative and cationic stresses they impose, rather than the additive effects of the individual stresses. It is conceivable that this potency might be driven by unanticipated interactions between stress signaling pathways that yield nonadditive molecular outputs.

Previously, we described an experimental platform for the analysis of combinatorial stresses in *C. albicans*, and we reported that the growth of this pathogen is significantly impaired by combinations of cationic and oxidative stresses (37). In this earlier study, we reported that cells exposed to a combination of cationic plus oxidative stresses displayed significantly extended adaptation times compared to cells treated with the corresponding individual stresses (37). Here we describe the effects of combinatorial cationic plus oxidative stress upon *C. albicans* viability, revealing that this fungus is exquisitely sensitive to this combinatorial stress. We confirm formally that the effects of the two stresses are synergistic. We then dissect the mechanistic basis for this extreme sensitivity, examining Cap1 and Hog1 functionality during exposure to combinatorial stress. We show that *C. albicans* cells are unable to activate their normal responses to oxidative and cationic stresses under these conditions and that this stress pathway interference is due largely to the inhibition of peroxide detoxification mechanisms by cations. Furthermore, we demonstrate that these mechanisms significantly enhance the anti-*C. albicans* potency of human neutrophils and, therefore, that fungal sensitivity to combinatorial cationic plus oxidative stress is critically important for the outcome of fungus-host interactions in the healthy individual.

## RESULTS

### *Candida albicans* is exquisitely sensitive to combinatorial cationic and oxidative stress.

**FIG 1:**
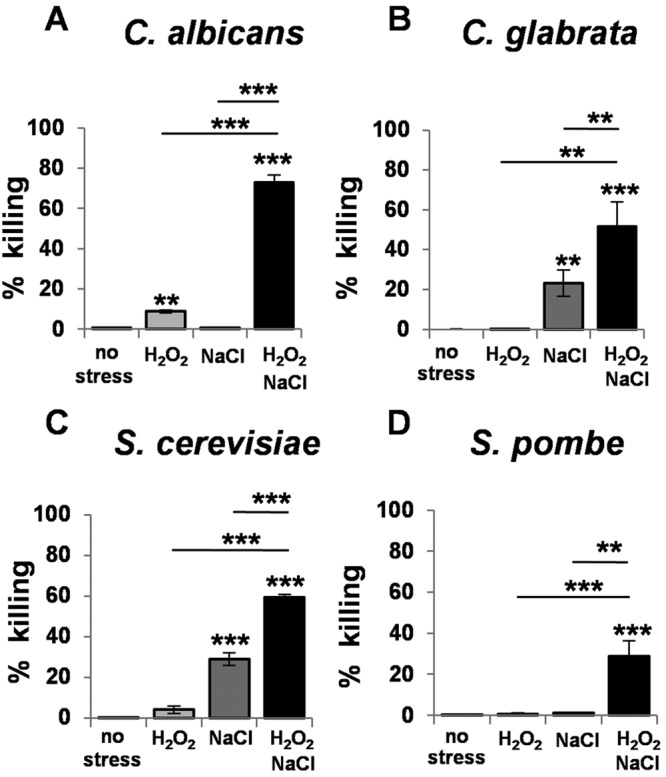
Combinatorial cationic and oxidative stress kills evolutionarily divergent yeasts. *Candida albicans* (CA372) (A), *Candida glabrata* (ATCC 2001) (B), *Saccharomyces cerevisiae* (BY4741) (C), and *Schizosaccharomyces pombe* (972) (D) (see Table S1 in the supplemental material) were grown to exponential phase and then exposed to stress for 4 h, and cell death was quantified by propidium iodide staining and FACS analysis for no stress, 5 mM H_2_O_2_, 1 M NaCl, or 1M NaCl plus 5 mM H_2_O_2_. Data are means standard deviations (*C. albicans*, *n* = 3; *C. glabrata*, *n* = 4; *S. cerevisiae*, *n* = 3; *S. pombe*, *n* = 4).

We reported previously that exposing *C. albicans* cells to combinatorial cationic plus oxidative stress results in extensive growth delays (37), but the impact of these combinatorial stresses upon cell viability has not been described. Therefore, we quantified the levels of killing imposed by a combination of cationic (1 M NaCl) and oxidative (5 mM H_2_O_2_) stresses by measuring the proportion of propidium iodide-positive cells via fluorescence-activated cell sorting (FACS) (see Materials and Methods). This dose of H_2_O_2_, which is used routinely (37), is roughly equivalent to the ROS concentrations generated by phagocytes (38). Also, 1 M NaCl induces a significant adaptive response in *C. albicans* while exerting minimal impacts on growth and viability (37). Simultaneous exposure to this combination of NaCl and H_2_O_2_ killed wild-type *C. albicans* cells (CA372) (see Table S1 in the supplemental material) much more effectively than the corresponding individual stresses (Fig. 1A), indicating that this yeast is particularly sensitive to this combination of cationic plus oxidative stresses.

We tested whether other yeasts showed sensitivity to combinatorial cationic plus oxidative stress. As expected (34, 36), the evolutionarily divergent yeasts tested, which included pathogens (*C. albicans* and *Candida glabrata*) and benign species (*Saccharomyces cerevisiae* and *Schizosaccharomyces pombe*), showed different degrees of sensitivity to NaCl and H_2_O_2_ stresses (Fig. 1). However, without exception, they all displayed a high degree of sensitivity to the combinatorial stress, suggesting that conserved molecular mechanisms might underlie the potency of combinations of cationic plus oxidative stresses.

**FIG 2:**
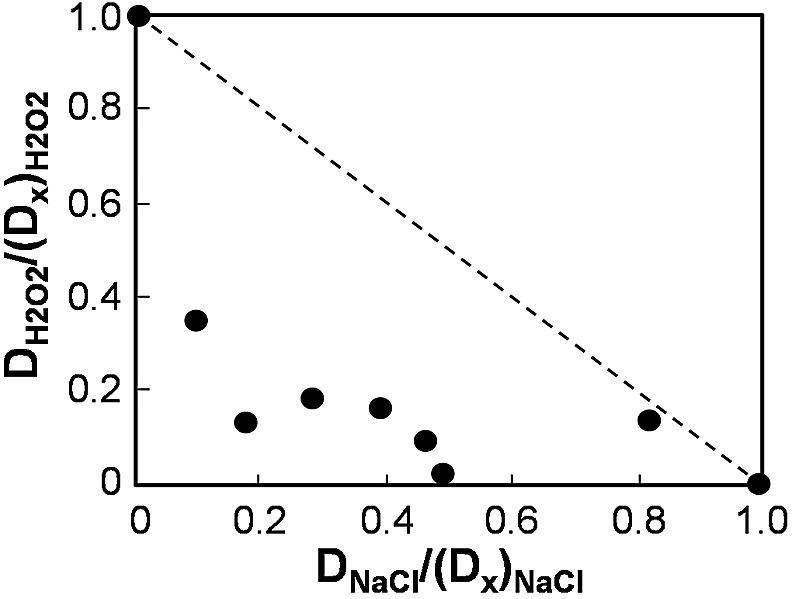
Cationic and oxidative stresses act synergistically to kill C. *albicans*. First, the dose-dependent impact of each individual stress upon *C. albicans* viability was determined by propidium iodide staining and FACS analysis. Using mathematical modeling, we then normalized the potency of each stress (e.g., D_NaCl_) relative to the dose required to achieve 100% killing [(D_X_)_NaCl_]. The impacts of different combinations of cationic plus oxidative stress were then experimentally determined, and the data were plotted in this normalized isobologram. If the two stresses acted in an additive fashion, the data points would lie on the diagonal between 0.1 and 1.0 (dotted line). The points lie well below this line, indicating formally that cationic and oxidative stresses act synergistically to kill *C. albicans*.

The exquisite sensitivity of *C. albicans* to combinatorial cationic plus oxidative stress suggested that these stresses might act synergistically. To test this formally, we examined the dose dependence of the individual and combinatorial stresses. Briefly, the dose-dependent effects of each individual stress upon *C. albicans* viability were quantified, and the doses required to achieve 100% killing [e.g., (D_X_)_H2O2_] were estimated mathematically (see Materials and Methods; also, see Fig. S2 in the supplemental material). The doses of the individual stresses (e.g., D_H2O2_) were normalized against these theoretical maximum doses [e.g., D_H2O2_/(D_X_)_H2O2_]. The impacts of different combinations of cationic plus oxidative stress upon *C. albicans* viability were determined experimentally, and these data were plotted in a normalized isobologram (Fig. 2). In principle, if oxidative and cationic stresses exerted additive effects upon *C. albicans* viability, the data points would lie on the dotted diagonal line (Fig. 2). However, the data points were well below this line, confirming that these stresses indeed act synergistically and not additively in achieving the killing of *C. albicans*.

### The normal cationic and oxidative stress transcriptomes are not induced in response to combinatorial stress.

We explored the molecular basis for the sensitivity of *C. albicans* cells to combinatorial cationic plus oxidative stress by genome-wide expression profiling. Wild-type *C. albicans* cells (CA372; see Table S1 in the supplemental material) were exposed to 1 M NaCl, 5 mM H_2_O_2_, or 1 M NaCl plus 5 mM H_2_O_2_ for 10 min, harvested, and subjected to microarray analysis (see Materials and Methods). Three independent replicates were analyzed for each condition, and the microarray output was validated by targeted analysis of specific transcripts (described below). The complete data set is presented in Table S2 in the supplemental material.

**FIG 3:**
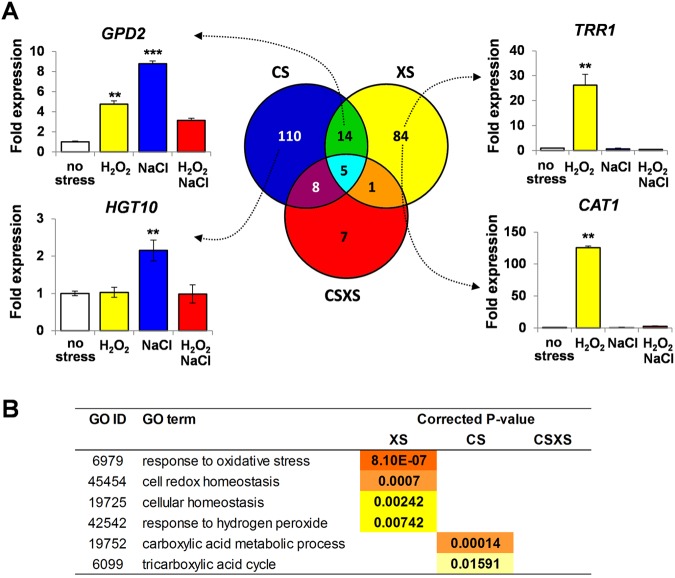
Transcript profiling of C. *albicans* following exposure to individual and combinatorial cationic and oxidative stresses. *C. albicans* (CA372) cells exposed to 1 M NaCl, 5 mM H_2_O_2_, or a combination of these stresses were subjected to microarray analysis. (A) Venn diagram presenting the numbers of genes that were consistently upregulated by ≥2.5-fold in three independent experiments. The microarray findings were validated by qRT-PCR analysis of individual transcripts (HGT10, GPD2, TRR1, and CAT1); their levels were quantified relative to the internal ACT1 mRNA control. Data are means standard deviations (*n* = 3). *, *P* ≤ 0.05; **, *P* ≤ 0.01; ***, *P* ≤ 0.001. (B) GO terms that were significantly enriched in the subsets of genes upregulated by the individual and combinatorial stresses. The microarray data set is presented in Table S2 in the supplemental material and are also available at ArrayExpress (http://www.ebi.ac.uk/arrayexpress/experiments/E-MEXP-3003/) under accession number E-MEXP-3003.

We focused on those *C. albicans* genes that were upregulated in response to the individual and combinatorial stresses, because the downregulated genes were not particularly informative. Indeed, no functional categories (GO terms) were significantly enriched in the subset of *C. albicans* genes that were downregulated in response to the combinatorial cationic plus oxidative stress (see Table S2 in the supplemental material). Of the 75 genes that were downregulated specifically in response to this stress, 40 have an unknown function. However, as expected (26), different subsets of *C. albicans* genes were upregulated in response to the individual cationic and oxidative stresses (Fig. 3A). Genes in the functional categories of response to oxidative stress, cell redox homeostasis, cellular homeostasis, and response to hydrogen peroxide were significantly enriched in the subset of genes that was upregulated in response to the oxidative stress (Fig. 3B). In contrast, genes involved in carboxylic acid metabolism and the tricarboxylic acid cycle were enriched in the osmotic stress-induced genes, which is consistent with previous work (26). Interestingly, the functional category “response to osmotic stress” did not show statistically significant enrichment, although some key osmotic stress genes were induced in response to 1 M NaCl. For example, *HGT10* (which encodes a permease involved in glycerol uptake) and *GPD2* (glycerol 3-P dehydrogenase) were activated, and this was confirmed by quantitative reverse transcription-PCR (qRT-PCR) (Fig. 3A). The transcriptional outputs of Hog1 are known to be environmentally contingent (26), and therefore the different conditions employed here compared to those in our previous study (26) may explain differences in the transcriptional response to NaCl. For example, here we used growth medium buffered at pH 7 (to permit direct comparisons with nitrosative stress responses, as part of a larger study [37]) and employed a higher dose of NaCl (a medium dose of 1.0 M [37], versus the low dose of 0.3 M used in our previous microarray study [26]). For example, *RHR2* (glycerol 3-phosphatase) was not significantly induced in the current study (see Table S2 in the supplemental material), and this was confirmed by qRT-PCR (data not shown). In contrast, *RHR2* is reproducibly induced by 0.3 M NaCl in a Hog1-dependent fashion in cells grown in unbuffered medium (~pH 6) (26).

Our comparison of the immediate transcriptional responses to combinatorial and individual stresses revealed dramatic differences between sets of upregulated *C. albicans* genes (Fig. 3A). Significantly, the cationic stress-induced transcriptome was largely abolished when cationic stress was combined with oxidative stress. Only 13 of the 137 cationic stress-induced genes were upregulated by the combinatorial stress (Fig. 3A). A similar trend was observed for the oxidative stress-induced transcriptome. Only a small fraction of the *C. albicans* genes that were induced by H_2_O_2_ alone were also activated when this oxidative stress was combined with cationic stress (6 of 104 genes) (Fig. 3A). The validity of this conclusion was confirmed by qRT-PCR and by Northern blotting of selected transcripts (Fig. 3A; see also Fig. S2 in the supplemental material). We conclude that when *C. albicans* cells were exposed to the combinatorial stress, they failed to activate the normally rapid transcriptional responses to individual oxidative or cationic stresses.

### Combinatorial cationic and oxidative stress affects Hog1 functionality.

**FIG 4:**
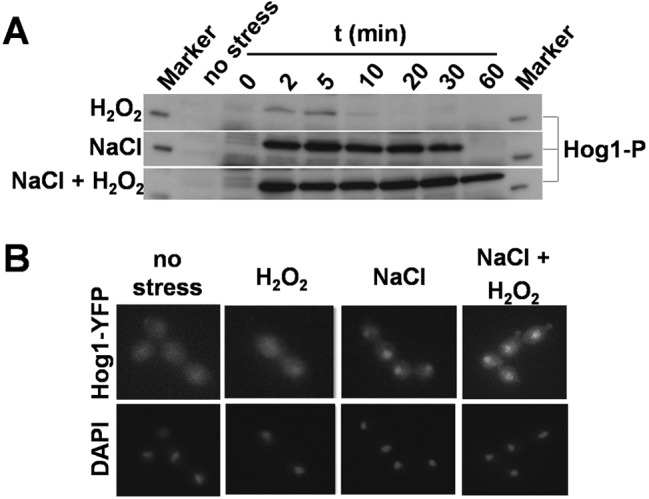
Impact of combinatorial cationic plus oxidative stress upon Hog1. *C*. *albicans* cells were exposed to 5 mM H_2_O_2_, 1 M NaCl, or 5 mM H_2_O_2_ plus 1 M NaCl. (A) Hog1 phosphorylation status in *C. albicans* (ML258) cells during exposure to stress, as revealed by Western blotting with a phospho-specific antibody. (B) Cellular localization of Hog1-YFP in *C. albicans* (JC63) cells determined by fluorescence microscopy before (no stress) and after 10 min of stress. The positions of nuclei were determined by DAPI staining.

Normally, Hog1 is activated in response to cationic stress via phosphorylation at its conserved TGY motif. Hog1 then contributes to the induction of many cationic stress-induced genes in *C. albicans*, leading to the induction of glycerol biosynthetic functions and ultimately to osmo-adaptation (19, 26–28). However, the expression of the Hog1 target genes *HGT10* and*GPD2* remained low during combinatorial cationic plus oxidative stress (Fig. 3A). Therefore, we examined the activation status of Hog1. Hog1 was strongly phosphorylated in *C. albicans* cells exposed to combinatorial stress (Fig. 4A), and Hog1-YFP accumulated in the nucleus (Fig. 4B). Hence, although Hog1 was activated under these conditions, some of its cationic stress targets were not. This reinforces the view (26) that the transcriptional outputs of Hog1 are influenced by the nature of the input signal.

Interestingly, Hog1 remained phosphorylated for an extended period in combinatorial stress-treated cells compared to cells treated with the cationic stress alone (Fig. 4A). This correlated with changes in the expression of Ptp2, a phosphatase that downregulates Hog1 (39). *PTP2* was induced about 4-fold in response to cationic stress, but no significant induction was observed following the combinatorial stress (see Table S2 in the supplemental material). Therefore, the lack of activation of *PTP2* might have contributed to the protracted Hog1 phosphorylation observed following combinatorial stress (Fig. 4A). Clearly, combinatorial stress influences Hog1 functionality.

### Cap1 activation is inhibited by combinatorial cationic and oxidative stress.

**FIG 5:**
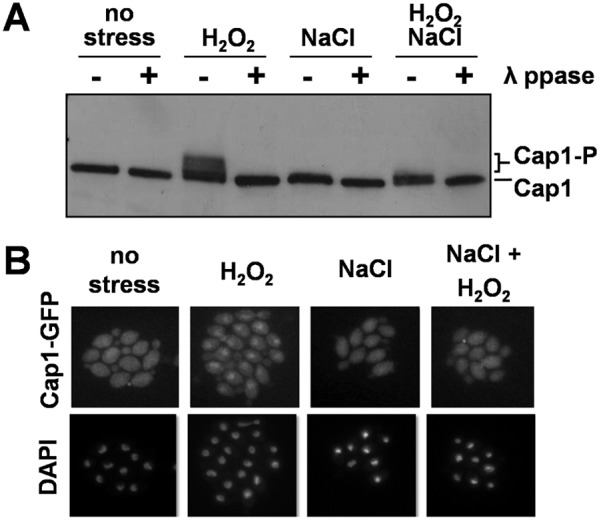
Impact of combinatorial cationic plus oxidative stress upon Cap1. *C*. *albicans* cells were exposed to 5 mM H_2_O_2_, 1 M NaCl, or 5 mM H_2_O_2_ plus 1 M NaCl. (A) Western blotting with an anti-Cap1 antibody to examine the gel shifts associated with Cap1 phosphorylation in *C. albicans* (JC948) cells after 10 min of stress. (Phosphatase controls are shown in Fig. S3 in the supplemental material.) (B) Localization of Cap1-GFP in *C. albicans* (JC1060) cells via fluorescence microscopy after 10 min of stress. DAPI staining revealed the positions of nuclei.

Cap1 is critical for oxidative stress gene activation in *C. albicans* (24–26). Cap1 becomes oxidized following exposure to oxidative stress (18, 20), which is thought to trigger a conformational change within the transcription factor that masks the accessibility of a nuclear export sequence, resulting in its nuclear accumulation and leading to Cap1-dependent gene expression (18–21, 25). Strikingly, Cap1-dependent genes such as *CAT1* (catalase) and *TRR1* (thioredoxin reductase), which were strongly induced in response to H_2_O_2_ alone, were not upregulated in *C. albicans* cells treated with combinatorial oxidative plus cationic stress (Fig. 3A). Therefore, we examined Cap1 nuclear accumulation following oxidative and combinatorial oxidative plus cationic stress treatments. Consistent with the lack of Cap1-dependent gene expression, Cap1-GFP failed to accumulate in the nucleus of *C. albicans* JC1060 cells following a 10-min combinatorial H_2_O_2_ plus NaCl stress treatment (Fig. 5A; see also Table S1 in the supplemental data). In contrast, cells exposed to H_2_O_2_ alone displayed the expected accumulation of Cap1-GFP in the nucleus. Cap1 phosphorylation, which correlates with its nuclear accumulation (21), was consistent with these nuclear accumulation profiles (Fig. 5A). Cap1 was phosphorylated after *C. albicans* JC948 cells were treated with H_2_O_2_ alone (Fig. 5B; see also Table S1 and Fig. S3 in the supplemental material), but not following exposure to the combinatorial stress treatment. We conclude that the Cap1 activation normally observed following oxidative stress does not occur when this same stress is combined with cationic stress (Fig. 5). As a result, the Cap1 regulon (including *CAT1* and *TRR1*) is not activated (Fig. 3).

### Catalase drives peroxide detoxification and is inhibited by cations, leading to intracellular ROS accumulation.

**FIG 6:**
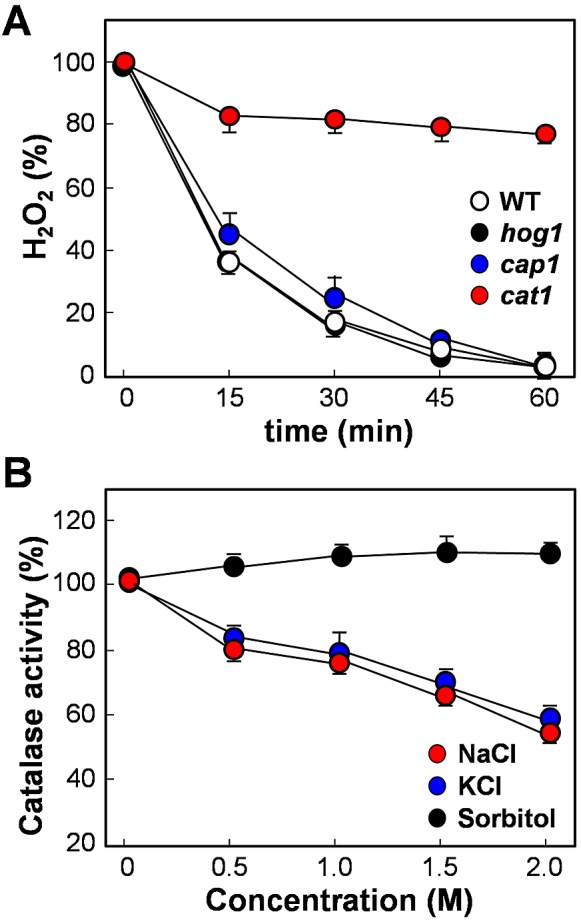
Catalase drives H_2_O_2_ detoxification and is inhibited by cations. (A) The detoxification of extracellular H_2_O_2_ by *C. albicans* cells is dependent on catalase. Data are means standard deviations (SD) for wild type (WT, CA372; *n* = 5); *cat1* (*n* = 4), *cap1* (*n* = 4), and *hog*1 (*n* = 3) (see Table S1 in the supplemental material). The starting concentration was 5 mM H_2_O_2_ (100%). (B) NaCl and KCl (but not sorbitol) inhibited catalase activity in wild-type *C. albicans* cells (CA372). Data are means SD (*n* = 3). Catalase activity is expressed relative to levels in the untreated controls.

To further investigate the mechanisms that underlie the exquisite combinatorial stress sensitivity of *C. albicans*, we examined the impact of key mutations upon peroxide detoxification. Wild-type *C. albicans* cells rapidly detoxified the exogenously added peroxide, clearing the 5 mM H_2_O_2_ within an hour (Fig. 6A). Interestingly, these high rates of H_2_O_2_ clearance were not impaired in *C. albicans hog1* or *cap1*null mutants, indicating that Hog1- and Cap1-mediated activation mechanisms are not required for rapid peroxide detoxification. This suggests that basal levels of key oxidative stress functions are sufficient to mediate this detoxification.

Catalase catalyzes the conversion of hydrogen peroxide to water and oxygen, and therefore we tested the impact of inactivating catalase (*CAT1*) on H_2_O_2_ clearance (Fig. 6A). H_2_O_2_ clearance was significantly inhibited in *C. albicans cat1* cells, indicating that detoxification is dependent on catalase. Significantly, *C. albicans cap1* and *hog1* mutant cells retained high basal levels of catalase in the absence of stress (see Fig. S4A in the supplemental material). Furthermore, genetic perturbation of the thioredoxin or glutaredoxin systems did not affect H_2_O_2_ detoxification rates (see Fig. S4B). We conclude that catalase is the main driver of H_2_O_2_ clearance under these conditions.

We then examined the effects of cations on basal catalase enzyme activity. First, protein extracts were prepared from untreated wild-type *C. albicans* cells (CA372; see Table S1 in the supplemental material). Then, we determined the effects of different concentrations of exogenously added NaCl, KCl, or sorbitol upon catalase activity (Fig. 6B). The sodium and potassium salts inhibited catalase, whereas sorbitol addition did not. In addition, cations reduced catalase enzyme levels in the longer term by inhibiting the Cap1-mediated induction of the *CAT1* gene during combinatorial cationic plus oxidative stress (Fig. 3). This suggests that the inhibition of catalase enzyme activity and levels, and hence peroxide detoxification, by cations might provide a mechanistic basis for the effects of this combinatorial stress.

### Ectopic catalase expression reduces intracellular ROS accumulation and suppresses the synergistic killing by combinatorial stress.

**FIG 7:**
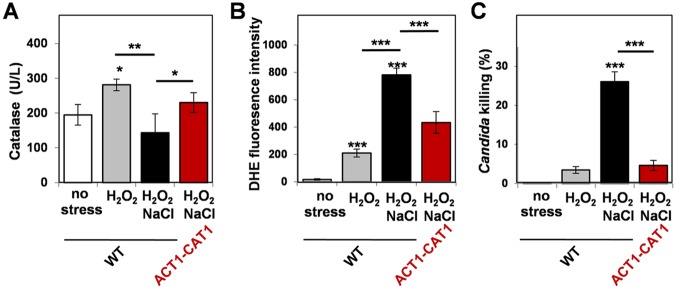
Ectopic catalase expression suppresses the elevated intracellular ROS levels and synergistic killing caused by combinatorial cationic plus oxidative stress. (A) Catalase activity was reduced in *C. albicans* wild-type (RM1000) cells following 60 min of combinatorial stress but was partially restored by ectopic catalase expression via ACT1-CAT1. Data are means standard deviations (*n* = 4) (see Table S1 in the supplemental material). (D) Intracellular ROS accumulation, assayed via dihydroethidium (DHE) fluorescence, was increased in *C. albicans* wild-type (RM1000) cells exposed to combinatorial stress for 60 min but was reduced in ACT1-CAT1 cells (*n* = 5). (E) Ectopic catalase expression suppressed the synergistic killing of *C. albicans* cells by combinatorial oxidative plus cationic stress. Cell death was quantified in wild-type (RM1000) and ACT1-CAT1 cells by propidium iodide staining and FACS analysis (*n* = 3). *P* values between 0.05 and 0.001 were considered: *, *P* ≤ 0.05; **, *P* ≤ 0.01; ***, *P* ≤ 0.001.

We examined the impact of combinatorial stress upon catalase levels. To achieve this, wild-type *C. albicans* cells (CA372; see Table S1 in the supplemental material) were treated with different stresses for 1 h, protein extracts were prepared, and catalase activities were measured (Fig. 7A). Significant levels of catalase were observed in untreated *C. albicans* cells and these levels were elevated in cells exposed to H_2_O_2_. Interestingly, catalase levels were not induced in cells exposed to H_2_O_2_ plus NaCl (Fig. 4B), which was consistent with the lack of Cap1-mediated activation of the catalase gene (*CAT1*) under these conditions (Fig. 3A).

We then tested whether these changes in catalase levels were reflected in altered intracellular accumulation of ROS (Fig. 7B). As expected, intracellular ROS levels increased following H_2_O_2_ treatment. Significantly, there was a dramatic elevation in intracellular ROS levels following exposure to the combinatorial oxidative plus cationic stress (Fig. 7B) which correlated with the reduction in catalase levels (Fig. 7A) and the correspondingly high levels of *C. albicans* killing (Fig. 7C).

These observations suggested that the inhibition of catalase might be a primary cause of the elevated ROS levels and the synergistic killing of *C. albicans* cells during combinatorial stress. To test this, we constructed an *ACT1-CAT1* fusion in *C. albicans* to allow ectopic expression of catalase independently of Cap1 function. The *C. albicans ACT1* promoter was inserted upstream of the *CAT1* coding region at the *CAT1* locus (see Materials and Methods). This ectopic catalase expression partially restored catalase levels during combinatorial stress (Fig. 7A) and led to a significant reduction in intracellular ROS levels (Fig. 7B). Significantly, the *ACT1-CAT1* fusion also suppressed the synergistic killing of *C. albicans* cells by combinatorial oxidative plus cationic stress (Fig. 7C). This indicates that the inhibition of catalase drives the elevated ROS levels and synergistic killing observed during combinatorial stress.

### Neutrophils exploit combinatorial cationic and oxidative stress to kill *C*. *albicans* cells efficiently.

**FIG 8:**
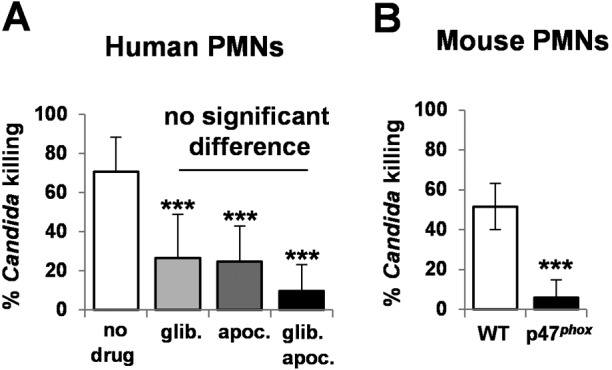
Phagocytic killing of C. *albicans* cells is dependent on cationic fluxes as well as the oxidative burst. (A) Killing of *C*. *albicans* (CA372) by human neutrophils was significantly decreased by drugs that inhibited the oxidative burst (apocynin) and cationic fluxes (glibenclamide). Data are means standard deviations (*n* = 8). Neutrophils were isolated from human blood and treated with 200 µM glibenclamide and/or apocynin. *C. albicans* cells were grown to exponential phase and incubated with neutrophils for 2 h at a ratio of 1:10 (*C. albicans*:neutrophils). (B) Neutrophils from p47^phox−/−^ mice did not kill *C. albicans* (CA372) efficiently (*n* = 4). Neutrophils were isolated from the bone marrow of wild-type and p47^phox−/−^ knockout mice, which lack NADPH oxidase activity. Exponentially growing *C. albicans* cells were incubated with these neutrophils for 2 h at a ratio of 1:10. *P* values between 0.05 and 0.001 were considered: *, *P* ≤ 0.05; **, *P* ≤ 0.01; ***, *P* ≤ 0.001.

We reasoned that the potency of neutrophils in killing *C. albicans* might be mediated by the combinatorial impact of the oxidative and cationic stresses they impose, rather than the effects of the individual stresses. To test this, we examined the effects of inhibiting the cationic fluxes and/or oxidative burst in human neutrophils upon their ability to kill *C. albicans* cells. We isolated neutrophils from healthy volunteers and then inhibited the oxidative burst in these neutrophils by using apocynin. Apocynin impairs the translocation of the p47^*phox*^ subunit of the NADPH oxidase, thereby preventing the formation of a functional phox (phagocyte oxidase) complex and, hence, H_2_O_2_ production (40). Meanwhile, cationic fluxes were blocked by using the drug glibenclamide, which inhibits potassium (K^+^) channels (41). Treating human neutrophils with a combination of apocynin plus glibenclamide impaired their ability to kill *C. albicans* cells (Fig. 8A). Significantly, *C. albicans* killing was impaired to similar extents by treating the neutrophils with either apocynin or glibenclamide alone (Fig. 8A). We confirmed that the effects of apocynin and glibenclamide treatments upon fungal killing were not due to any effects of these drugs on phagocytosis, which was not affected (see Fig. S4 in the supplemental material). If the effects of cationic and oxidative stress in the neutrophil were additive, the pharmacological attenuation of cationic fluxes would be expected to exert limited effects upon neutrophil killing, given the resistance of *C. albicans* cells to cations *in vitro* (28, 36, 37). However, the data clearly indicated that cationic fluxes, like the oxidative burst, contribute significantly to the potency of human neutrophils, because the inhibition of either the oxidative burst or the cationic flux was sufficient to attenuate their fungicidal activity by about 3-fold (Fig. 8A). This suggests that the killing of *C. albicans* by neutrophils is driven mainly by the combinatorial effects of the oxidative burst and cationic flux, rather than by the effects of each individual stress.

If combinatorial stress drives the potent killing of *C. albicans* cells by neutrophils, then the protective effects of ectopic catalase expression observed *in vitro* (Fig. 7C) should also be observed *ex vivo* in the protection of the fungus against neutrophils. As predicted, ectopic catalase expression reduced the killing of *C. albicans* cells by mouse neutrophils by 31.8% (*P* ≤ 0.039). As a further test of the model, we tested the prediction that genetic mutations which inactivate the oxidative burst should attenuate the ability of neutrophils to kill *C. albicans*. As predicted, bone marrow-derived neutrophils from p47 ^*phox*−/−^ mice, which display defective phagocytic NADPH oxidase activity and ROS production, displayed significantly reduced killing compared to wild-type neutrophils (Fig. 8B). This is consistent with the observation that patients with chronic granulomatous disease (CGD), caused by an inherited NADPH oxidase mutation (22), often succumb to microbial infection (23).

## DISCUSSION

**FIG 9:**
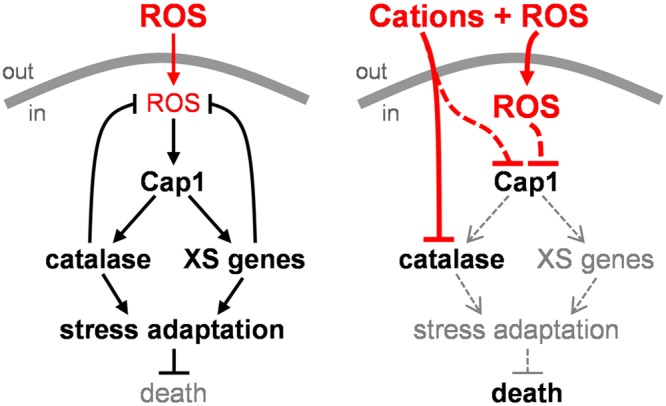
Impact of combinatorial stress on oxidative stress adaptation. When *C. albicans* cells are exposed to an oxidative stress, Cap1 mediates the activation of catalase and other oxidative stress genes, leading to ROS detoxification, stress adaptation, and survival (left panel). However, when *C. albicans* cells are exposed to combinatorial stress, for example, during phagocytosis by neutrophils, the cations inhibit catalase, thereby reducing H_2_O_2_ detoxification and leading to intracellular ROS accumulation. This prevents the activation of Cap1, leading to a precipitous collapse in oxidative stress adaptation and cell death. Cations may also affect Cap1 activity by other ROS-independent mechanisms (see the text).

In this study, we demonstrated that *C. albicans* cells are killed synergistically by combinatorial oxidative plus cationic stress (Fig. 2), and we investigated the mechanistic basis for the nonadditive effects of this combinatorial stress (Fig. 9). The exquisite sensitivity of *C. albicans* cells is caused by stress pathway interference, which is largely mediated by the inhibitory effects of cations upon catalase (Fig. 5B). This leads to the hyperaccumulation of intracellular ROS (Fig. 7B). Importantly, the Cap1 activation that is normally seen in response to oxidative stress (25, 26) was not observed when cells are exposed to combinatorial cationic plus oxidative stress (Fig. 5). Cations might also interfere with Cap1 activation in other ways, for example, indirectly via the inhibition of Cap1-activating proteins like Gpx3 or Ybp1 (Fig. 9). These mechanisms are not mutually exclusive. As a consequence of Cap1 inhibition, *C. albicans* cells did not activate their normal transcriptional response to oxidative stress in the presence of cations (Fig. 3). Combinatorial oxidative plus cationic stress also affected Hog1 functionality (Fig. 4) and the normal transcriptional response to cationic stress (Fig. 3). However, the extreme sensitivity of *C. albicans* cells to this combinatorial stress appears to be manifested by an impaired oxidative stress adaptation, because ectopic expression of catalase reduces the hyperaccumulation of ROS and suppresses the hypersensitivity to the combinatorial stress (Fig. 7). These mechanisms may be conserved in pathogenic and nonpathogenic yeasts (Fig. 1), as *S. cerevisiae*, *S.* pombe, and *C. glabrata* all possess AP-1-like transcription factors that contribute to their transcriptional responses to oxidative stress (ScYap1, SpPap1, and CgYap1, respectively), and all express catalase as part of these adaptation mechanisms (ScCta1, SpCtt1, and CgCta1). These mechanisms may also be conserved in the plant kingdom, where salt treatment can lead to ROS accumulation in roots (42, 43).

We note that although combinatorial stress results in synergistic killing in *C. albicans*, *S.* cerevisiae, *C. glabrata*, and *S. pombe*, the species-specific responses to such stresses may be influenced by structural and dose-dependent differences in their stress regulatory networks. These yeasts display differential sensitivities to oxidative stress (34, 36). They also display significant differences in some oxidative and cationic stress signaling components (44–46), as well as divergence in their core transcriptional responses to stress (19, 47). *S. cerevisiae*, *C. glabrata*, and *S. pombe* activate large sets of core stress genes (>100) in response to diverse environmental stresses (48–51), whereas *C. albicans* displays a limited core transcriptional response to stress that comprises about 20 genes (26, 52). Metabolic differences, such as different rates of respiration and energy production and NADPH, glycerol, trehalose, and glutathione synthesis (53–56), may also affect combinatorial stress adaptation in these yeasts.

The sensitivity of *C. albicans* to combinatorial oxidative plus cationic stress is not simply a general consequence of exposing the fungus to any combination of stressors, because other types of combinatorial stress do not exert synergistic effects on *C. albicans*. For example, *C. albicans* cells are sensitive to nitrosative stress as well as to oxidative stress, but they are not especially sensitive to combinatorial nitrosative plus oxidative stress (37). Therefore, different combinations of stressors exert different effects on *C. albicans* fitness and viability, with only some combinations leading to nonadditive, synergistic effects.

Our data have significant implications for the interpretation of genomic data sets generated from heterogeneous populations of cells derived from complex *in vivo* systems. For example, the lack of activation of *C. albicans* oxidative stress genes in some host niches (38) may now be interpreted in at least two ways. This observation could imply that the fungal cells are not exposed to an oxidative stress in a particular host niche. However, an alternative and plausible explanation based on our observations (Fig. 3) would be that the cells may have been exposed to a combinatorial oxidative plus cationic stress that prevented the normal activation of oxidative stress genes. We suggest that the activation of oxidative stress genes by *C. albicans* cells during phagocytic interactions (15, 16) is due to their exposure to oxidative stress prior to engulfment (57) rather than the combinatorial stress they experience after phagocytosis (Fig. 3 and 8).

We have provided evidence that stress pathway interference accounts for the potency of neutrophils and their prevention of systemic fungal infection (Fig. 8). Our results indicate that the extreme sensitivity of *C. albicans* cells to combinatorial cationic plus oxidative stress is exploited by neutrophils as a potent antimicrobial weapon. Inhibition of either the oxidative burst or cationic fluxes reduces the potency of human neutrophils to similar extents (Fig. 8). Furthermore, the ectopic expression of catalase, which reduces the potency of combinatorial cationic plus oxidative stress *in vitro* (Fig. 7C), also decreases the potency of neutrophils*.* Therefore, the inhibition of *C. albicans* catalase by cations appears to lie at the heart of this fungicidal mechanism (Fig. 9). Consequently, pharmacological inhibitors that block fungal catalases might provide an effective means of enhancing immune defenses and complementing antifungal therapies.

Host niches are complex and dynamic and provide numerous environmental inputs for an invading microbial pathogen. In this study, we have addressed the impact of combinatorial oxidative plus cationic stress. However, we predict that additional environmental cues, such as variations in temperature or nutrient availability (58, 59), might also influence combinatorial stress outputs in *C. albicans*. This study provides a platform for future studies that will address the combinatorial impacts of various environmental conditions and additional stresses upon the adaptive responses of *C. albicans* and other pathogenic microbes, including the challenges imposed by the innate immune system.

## MATERIALS AND METHODS

### Strains, growth conditions, and stress induction.

The strains used in this study are listed in Table S1 in the supplemental material. *Candida* cells were grown in Tris-buffered yeast extract-peptone-dextrose medium (YPDT; pH 7.4) as described previously (37). Osmotic stress was applied with 1 M NaCl and oxidative stress was applied with 5 mM hydrogen peroxide (H_2_O_2_) unless stated otherwise.

### Viability assays.

Cell viability was determined by propidium iodide staining and FACS analysis (FACSCalibur; Becton, Dickinson, CA) as described previously (37).

### Modeling of synergistic interactions.

To test for potential synergy between oxidative and osmotic stresses in a quantitative framework, we first quantified the dose-dependent impacts of individual stresses upon *C. albicans* CA372 viability (60). Using mathematical modeling, we then normalized the potency of the individual cationic and oxidative stress doses relative to the dose predicted to achieve 100% killing (*D_x_*) (see Fig. S2 in the supplemental material). We then experimentally determined the impacts of different combinations of oxidative and osmotic stresses and plotted these data in a normalized isobologram in which the additive effects of these stresses were represented by the diagonal between 0.1 and 1.0 (60).

### Microarrays.

*C. albicans* CA372 cells were grown to exponential phase in YPDT at 30°C, 200 rpm, and exposed to 1 M NaCl, 5 mM H_2_O_2_, or 5 mM H_2_O_2_ plus 1 M NaCl for 10 min. RNA was extracted and cDNA was prepared, labeled, and hybridized to *C. albicans* microarrays as described previously (26). Slides were scanned using a proScanArray HT system (PerkinElmer Life Sciences, Beaconsﬁeld, United Kingdom) and quantiﬁed using ScanArray Express (version 4). Data were normalized and analyzed using GeneSpringGX (version 11; Agilent Technologies Inc., CA), and significant changes (>2.5-fold) in gene expression levels were visualized in a Volcano plot (*t* test, *P* < 0.05). Data from three independent biological replicates were used for each analysis, and the gene expression ratios were determined by comparing the stressed cells to the unstressed control (26).

### Transcript levels.

*C. albicans* CA372 cells were grown to exponential phase in YPDT at 30°C, 200 rpm, and exposed to 1 M NaCl, 5 mM H_2_O_2_, or 5 mM H_2_O_2_ plus 1 M NaCl for 10 min. RNA was extracted using standard procedures (26), cDNA was generated by using the SuperScript II reverse transcriptase (Invitrogen), and qRT-PCR was performed using a Light Cycler (Roche). Transcript levels were measured relative to the internal *ACT1* mRNA control (26, 28). The qRT-PCR primers and probes are described in Table S3 in the supplemental material.

### Western blotting.

*C. albicans* was grown to exponential phase in YPDT at 30°C, 200 rpm, and exposed to 5 mM H_2_O_2_, 1 M NaCl, or 5 mM H_2_O_2_ plus 1 M NaCl. Cells were harvested before (no stress) and after stress was imposed, at the indicated times, protein extracts were prepared, and Western blotting was performed as described previously (28). Hog1 phosphorylation was detected by using an anti-phospho-p38 mitogen-activated protein kinase (Thr180/Tyr182) XP rabbit antibody (New England Biolabs, United Kingdom) (26, 28). Blots were then probed with a secondary anti-rabbit IgG, horseradish peroxidase-linked antibody (New England Biolabs, United Kingdom). Blots were washed with Tween-PBS and developed using LumiGlo (New England Biolabs, United Kingdom) according to the manufacturer’s instructions.

To detect Cap1 phosphorylation, *C. albicans* cells expressing Cap1 tagged with a 2myc-6His epitope at the C terminus (JC948; see Table S1 in the supplemental material) were grown to exponential phase in YPDT, and samples were collected after 10 min of stress treatment, as described above. Proteins were extracted and enriched on Ni-nitrilotriacetic acid–agarose before analysis by SDS-PAGE and Western blotting with anti-Myc antibodies (18).

### Protein localization.

*C. albicans* cells expressing a Hog1-yellow fluorescent protein (Hog1-YFP) fusion (JC63; see Table S1 in the supplemental material) or a Cap1-green fluorescent protein (Cap1-GFP) fusion (JC1060) were grown to exponential phase in YPDT, and samples were collected after 10 min of stress treatment as described above. Hog1-YFP and Cap1-GFP localization was determined by fluorescence microscopy (26, 28). The positions of the nuclei were determined via 4′,6-diamidino-2-phenylindole (DAPI) staining.

### H_2_O_2_ detoxification assay.

*C. albicans* CA372 and *cat1*, *cap1*, and *hog1* mutant cells (see Table S1 in the supplemental material) were grown to exponential phase in YPDT and exposed to 5 mM H_2_O_2_. The concentration of H_2_O_2_ in the medium was measured at the time points indicated in a colorimetric peroxide assay (QuantiChrom peroxide assay kit; BioAssay Systems) following the manufacturer’s instructions.

### Catalase activity.

*C. albicans* CA372 cells were grown to exponential phase in YPDT and exposed to 1 M NaCl, 5 mM H_2_O_2_, or 1 M NaCl plus 5 mM H_2_O_2_. Cells were harvested before (no stress) and after 60 min of stress, and catalase activity was measured in a colorimetric catalase assay (EnzyChrom catalase assay kit; BioAssay Systems) according to the manufacturer’s instructions.

### PMN assays.

Polymorphonuclear granulocytes (PMNs) were isolated from human blood by using Histopaque-1119 and Histopaque-1077 buffers (Sigma-Aldrich) following the manufacturer’s instructions. PMNs were isolated from the bone marrow of wild-type (C57BL/6) and p47 ^*phox−/−*^ knockout mice as described previously (61). For some experiments PMNs were treated with 200 µM glibenclamide or apocynin for 2 h before exposure to *C. albicans*. *C. albicans* CA372 cells were grown in YPDT at 30°C, 200 rpm, to exponential phase, washed in PBS, and incubated with neutrophils for 2 h at a ratio of 1:10 (*Candida*: neutrophils) in RPMI medium at 37°C, 5% CO_2_, and then cell viability measured.

### Phagocytosis assay.

*C. albicans* cells were stained with fluorescein isothiocyante (FITC) and coincubated with PMNs for 60 min at 37°C, 5% CO_2_. Calcofluor white (CFW) was added to the mixture immediately before FACS processing with a BD LSR II apparatus. CFW stained the *C. albicans* cells outside the neutrophils. The percentage of phagocytosed *C. albicans* cells (FITC positive, CFW negative) was calculated.

### Statistical analyses.

SPSS version 19 for Windows was used for the statistical analyses. The values of the treated samples were compared against those for the control (untreated sample) by a one-way analysis of variance and Dunnett’s two-sided *t* tests. Associations within treated samples were determined by an independent samples *t* test. The *P* values were determined and are indicated as follows: *, *P* ≤ 0.05; **, *P* ≤ 0.01; ***, *P* ≤ 0.001.

### Microarray data accession number.

The complete data set is available in a MIAME-compliant format at ArrayExpress (accession number E-MEXP-3003) and also in Table S2 in the supplemental material.

## SUPPLEMENTAL MATERIAL

Figure S1 Mathematical modeling reveals synergistic killing by cationic and oxidative stresses. Download Figure S1, PDF file, 0.1 MB

Figure S2 Cap1-dependent gene expression is inhibited by combinatorial cationic plus oxidative stress. Download Figure S2, PDF file, 0.1 MB

Figure S3 Basal catalase activities and rate of H_2_O_2_ detoxification in *C. albicans* cells. Download Figure S3, PDF file, 0.1 MB

Figure S4 The phagocytosis of *C. albicans* cells by human neutrophils is not significantly affected by their treatment with 200 µM glibenclamide or apocynin. Download Figure S4, PDF file, 0.1 MB

Table S1 *C. albicans* strains used in this study. Table S1, PDF file, 0.2 MB.

Table S2 Microarray data set. Table S2, PDF file, 0.5 MB.

Table S3 qRT-PCR primers and probes. Table S3, PDF file, 0.1 MB.
