# Supplementary material for: Mechanisms Underlying the Exquisite Sensitivity of Candida albicans to Combinatorial Cationic and Oxidative Stress That Enhances the Potent Fungicidal Activity of Phagocytes
Source: mBio. 2014 Jul 15;5(4):e01334-14. doi: 10.1128/mBio.01334-14 (PMC4161263; doi:10.1128/mBio.01334-14)
Supplement: Figure S1 — Mathematical modeling reveals synergistic killing by cationic and oxidative stresses. Download [file mbo004141905sf01.pdf]

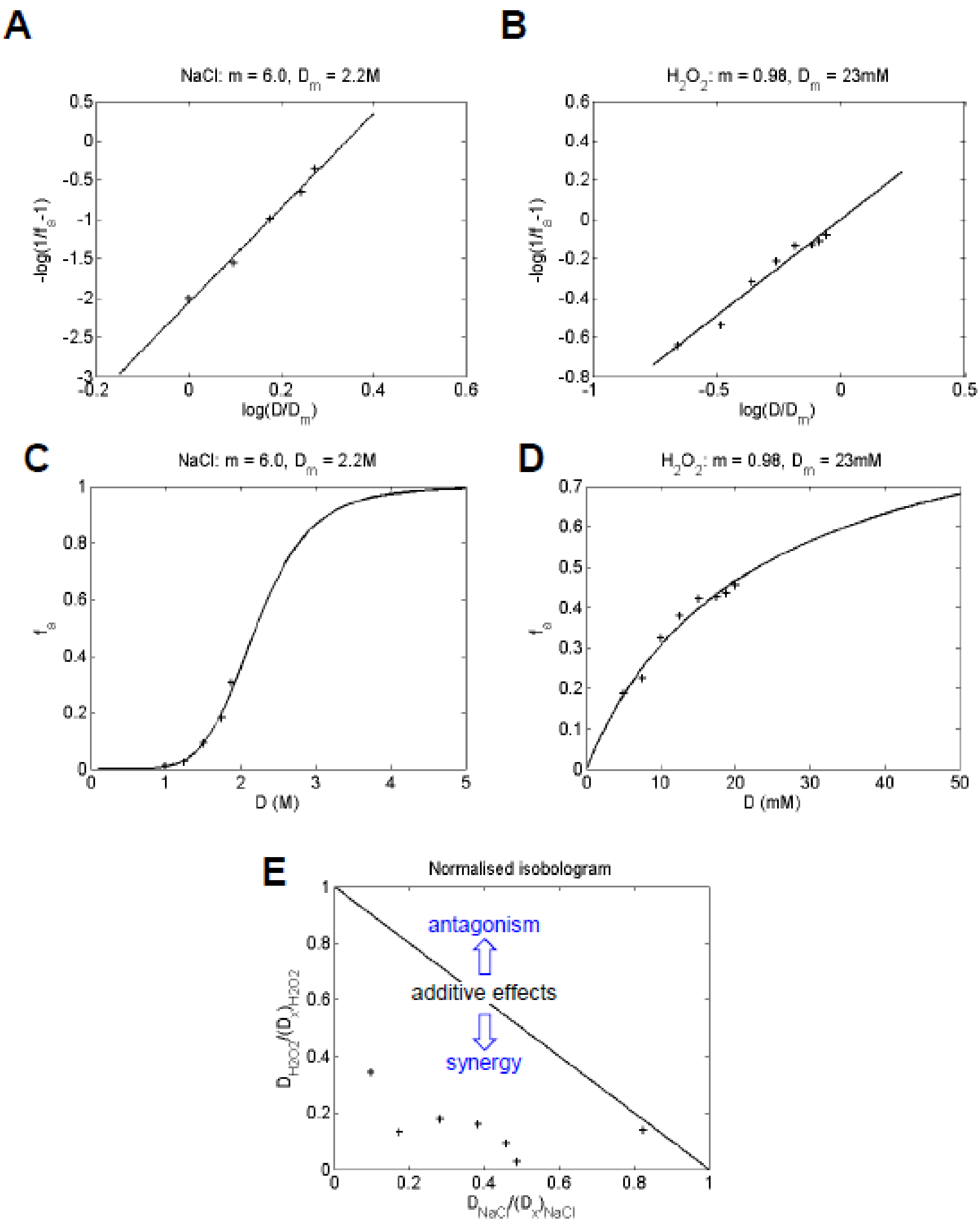

Figure S1. Mathematical modelling reveals synergistic killing by cationic and oxidative stresses.

To identify synergism/antagonism between different stresses in a quantitative framework, we first investigated how individual stress affects viability of a *C. albicans* population across a range of doses. The median-effect equation [1] relates viability assay results (fraction of dead cells: f_a, fraction of live cells: f_u, $f_a + f_u = 1$) with potency of the stress (median effect dose: the dose required at 50% inhibition D_m) and a Hill-type coefficient m (Equation 1):

$$f_a / f_u = (D/D_m)^m . \quad (1)$$

This equation can be rearranged into a different form (Equation 2), which can be used to fit median effect dose D_m and Hill type coefficients to the experimental data through linear regression.

$$\log(f_a / f_u) = m \cdot [-\log(D_m) + \log(D)] . \quad (2)$$

(**A, B**) The viability assay results are plotted in log scale (n=3). The high linearities of the median effect plots (R^2 for the two conditions: NaCl: 0.99; H_2O_2: 0.97) guarantees the legitimacy of median-effect equation to the experimental data. The median effect dose D_m (NaCl: 2.2 M; H_2O_2: 23 mM) and Hill-type coefficients m (NaCl: 6.0; H_2O_2: 0.98) are obtained by linearity regression. (**C, D**) The dose-effect curves for experimental data and prediction results in natural scale illustrate sigmoidicity and hyperbolicity of the two stresses, respectively.

C. albicans cells exhibit different responses to cationic and oxidative stress. Cell death due to NaCl is highly sigmoidal (m=6, Figure 1C). The inhibitory effect of NaCl becomes pronounced beyond 1 M NaCl, and starts to saturate at about 3 M. In contrast, the effects of H_2O_2 are purely hyperbolic and demonstrate a more linear relationship with dosage (m=0.98, Figure 1D).

We next studied the nature of the combinatorial conditions. For each dose in a particular combination, we computed D_x, the dose of a stress that is required to generate the inhibition under the combinatorial condition by applying this type of stress alone, using Equation 3 which follows directly from Equation 1:

$$D_x = D_m \cdot \left(f_a^{combi} / f_u^{combi} \right)^{\frac{1}{m}} . \quad (3)$$

Next, we normalised each dose in a combinatorial condition with respect to its corresponding D_x. This way, we may graph different combinations on the same diagram, known as a normalised isobologram (**E**). A point on the isobol (the straight line crossing (0,1) and (1,0)) demonstrates additive effect–each normalised dose sums to 1, and the combined condition inhibits just as applying each condition alone at their respective D_x levels. Any point beyond the isobol indicates antagonism, while any point below the isobol demonstrates synergism. The cationic and oxidative stresses exhibit strong synergism, as the combinatorial conditions are generally far off the isobol.
