# Supplementary material for: Mechanisms Underlying the Exquisite Sensitivity of Candida albicans to Combinatorial Cationic and Oxidative Stress That Enhances the Potent Fungicidal Activity of Phagocytes
Source: mBio. 2014 Jul 15;5(4):e01334-14. doi: 10.1128/mBio.01334-14 (PMC4161263; doi:10.1128/mBio.01334-14)
Supplement: Figure S2 — Cap1-dependent gene expression is inhibited by combinatorial cationic plus oxidative stress. Download [file mbo004141905sf02.pdf]

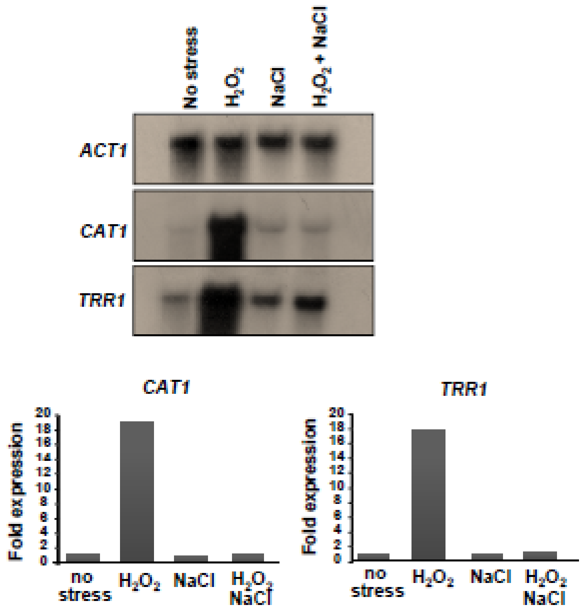

Figure S2. Cap1 dependent gene expression is inhibited by combinatorial cationic plus oxidative stress.

Northern analysis of RNA isolated from wild type *C. albicans* cells (JC747) grown in YPDT pH7.4 at 30°C and exposed to stress for 10 min: no stress; 5 mM H_2O_2; 1 M NaCl; or 5 mM H_2O_2 plus 1 M NaCl. Northern blots were probed for the Cap1-dependent genes *CAT1* and *TRR1,* as well as the *ACT1* mRNA internal loading control. The levels of the *CAT1* and *TRR1* RNAs were quantified relative to the *ACT1* control.
