# Supplementary material for: Mechanisms Underlying the Exquisite Sensitivity of Candida albicans to Combinatorial Cationic and Oxidative Stress That Enhances the Potent Fungicidal Activity of Phagocytes
Source: mBio. 2014 Jul 15;5(4):e01334-14. doi: 10.1128/mBio.01334-14 (PMC4161263; doi:10.1128/mBio.01334-14)
Supplement: Figure S3 — Basal catalase activities and rate of H2O2 detoxification in C. albicans cells. Download [file mbo004141905sf03.pdf]

A

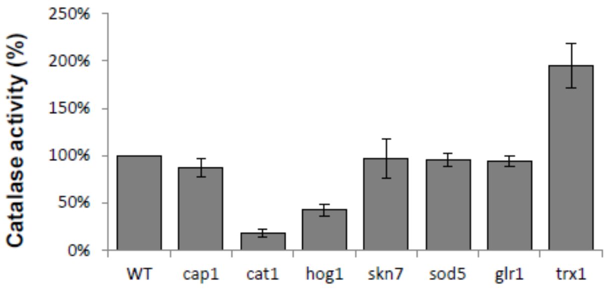

B

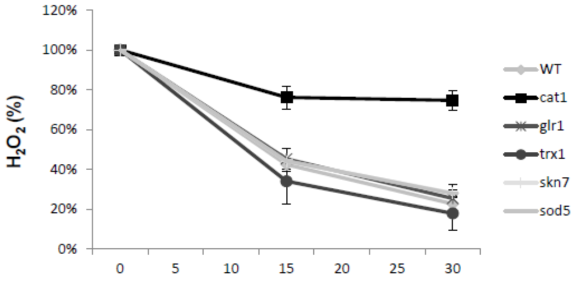

Figure S3. Basal catalase activities and rate of H_2O_2 detoxification in *C. albicans* cells.

(**A**) The basal catalase activity was measured in wild type (CA372) (n=6), *cap1* (n=3), *cat1* (n=3), *hog1* (n=6), *skn7* (n=3), *sod5* (n=3), *glr1* (n=3) and *trx1* (n=6) strains (Table S1) before exposure to stress (mean ± SD).

(**B**) The rate of detoxification of extracellular H_2O_2 by *C. albicans* cells is not affected by disrupting the thioredoxin or glutaredoxin systems.The rate of extracellular detoxification of H_2O_2 was measure in wild type (CA372) (n=5), *cat1* (n=4), *glr1* (n=4), *trx1* (n=4), *skn7* (n=4) and *sod5* (n=3), strains (Table S1) (mean ± SD).
