# Supplementary material for: Mechanisms Underlying the Exquisite Sensitivity of Candida albicans to Combinatorial Cationic and Oxidative Stress That Enhances the Potent Fungicidal Activity of Phagocytes
Source: mBio. 2014 Jul 15;5(4):e01334-14. doi: 10.1128/mBio.01334-14 (PMC4161263; doi:10.1128/mBio.01334-14)
Supplement: Figure S4 — The phagocytosis of C. albicans cells by human neutrophils is not significantly affected by their treatment with 200 µM glibenclamide or apocynin. Download [file mbo004141905sf04.pdf]

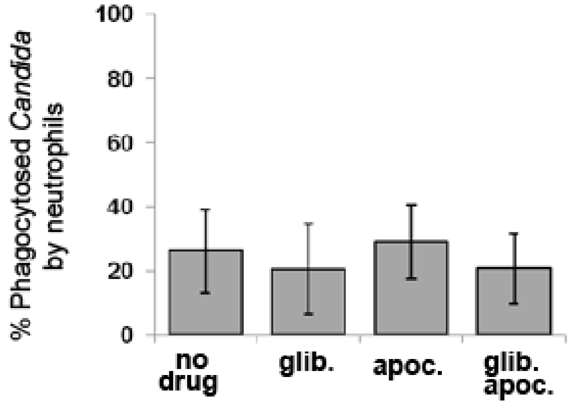

Figure S4. The phagocytosis of *C. albicans* cells by human neutrophils is not significantly affected by their treatment with 200 μM glibenclamide or apocynin.

C. albicans cells were labeled with FITC, co-incubated with blood-derived human neutrophils for 60 min, and the rate of phagocytosis determined by FACS analysis (mean ± SD, n=3).
