# Supplementary material for: Mechanisms Underlying the Exquisite Sensitivity of Candida albicans to Combinatorial Cationic and Oxidative Stress That Enhances the Potent Fungicidal Activity of Phagocytes
Source: mBio. 2014 Jul 15;5(4):e01334-14. doi: 10.1128/mBio.01334-14 (PMC4161263; doi:10.1128/mBio.01334-14)
Supplement: Table S1 — C. albicans strains used in this study. [file mbo004141905st1.pdf]

Table S1

Table S1: Strains used in this study

Strain	Genotype	Source
Candida albicans		
SC5314	Clinical isolate	[2]
CAI4	*ura3::λimm434/ura3::λimm434* (SC5314 lineage)	[3]
RM1000	*ura3::λimm434/ura3::λimm434, his1::hisG/his1::hisG*	[4]
BWP17	*ura3::λimm434/ura3::λimm434, his1::hisG/his1::hisG, arg4::hisG/arg4::hisG*	[4]
CA372	*ura3::λimm434/ura3::λimm434, RPS1-CIp10(URA3)*	[5]
CA674	*ura3::λimm434/ura3::λimm434, his1::hisG/his1::hisG, RPS1-CIp20(URA3,HIS1)*	[6]
CA1206	*ura3::λimm434/ura3::λimm434, his1::hisG/his1::hisG, arg4::hisG/arg4::hisG, RPS1-CIp30(URA3,HIS1,ARG4)*	[6]
ML258	*ura3::λimm434/ura3::λimm434, RPS1-pACT1-FLAG-GFP*	[7]
SN148	*arg4/arg4, leu2/leu2, his1/his1, ura3::λimm434/ura3::λimm434, iro1::λimm434/iro1::λimm434*	[8]
JC45	*ura3::λimm434/ura3::λimm434, his1::hisG/his1::hisG, hog1::loxP-ura3-loxP/hog1::loxP-HIS1-loxP*	[9]
JC63	*ura3::λimm434/ura3::λimm434, his1hisG/his1hisG, HOG1-YFP-URA3/HOG1-YFP-HIS1*	[10]
JC677	*trx1::loxP-ARG4-loxP/trx1::loxP- HIS1-loxP, RPS1-CIp10 (URA3)*	[11]
JC747	*arg4/arg4, leu2/leu2, his1/his1, ura3::λimm434/ura3::λimm434, iro1::λimm434/iro1::λimm434, RPS1-CIp30*(*URA3 HIS1 ARG4*)	[11]
JC842	*ura3::λimm434/ura3::λimm434, his1::hisG/his1::hisG, arg4::hisG/arg4::hisG, cap1::loxP-ARG4-loxP/cap1::loxP-HIS1-loxP, RPS1-CIp10(URA3)*	This study
JC948	*arg4/arg4, leu2/leu2, his1/his1, ura3::λimm434/ura3::λimm434, iro1::λimm434/iro1::λimm434, CAP1-MH–URA3*	[11]
JC1060	*arg4/arg4, leu2/leu2, his1/his1, ura3::λimm434/ura3::λimm434, iro1::λimm434/iro1::λimm434, CAP1-GFP–URA3*	[11]
CA1864	*ura3::λimm434/ura3::λimm434, his1::hisG/his1::hisG, cta1::loxP-URA3-loxP/cta1::HIS1*	This study
ACT1-CAT1	*ura3::λimm434/ura3::λimm434*, *his1::hisG/his1::hisG, CTA1/URA3-prACT1-CTA1*	This study
*cta4*Δ	*SN152, cta4Δ* ::*CmLEU2/cta4Δ::CdARG4*, *arg4Δ::dpl200/arg4Δ::dpl200, leu2Δ::dpl200/leu2Δ::dpl200*	[12]
*skn7*Δ	*ura3::λimm434/ura3::λimm434, skn7::hisG/skn7::hisG-URA3-hisG*	[13]
*sod5*Δ	*arg4/arg4, leu2/leu2, his1/his1, URA3/ura3::λimm434, IRO1/iro1::λimm434*, *sod5::cmLEU2/sod5::CdHIS1*	[14]
*glr1*Δ	*glr1/glr1, ura3::λimm434/ura3::URA3, his1::hisG/his1::hisG*, *arg4::hisG/arg4::hisG*	From Ben Distel
Candida glabrata		
CBS138	*Clinical isolate* (ATCC 2001)	[15]
Saccharomyces cerevisiae		
BY4741	*MATa, his3, leu2, met15, ura3*	Euroscarf
Schizosaccharomyces pombe		
972	Environmental isolate	[16]

Supplemental References

1. **Chou, T. C. and Talalay, P.** 1984. Quantitative analysis of dose-effect relationships: the combined effects of multiple drugs or enzyme inhibitors. Adv. Enzyme. Regul. **22**: 27-55.
2. **Gillum, A. M., Tsay, E. Y. H. and Kirsch, D. R.** 1984. Isolation of the *Candida albicans* gene for orotidine-5'-phosphate decarboxylase by complementation of *S. cerevisiae ura3* and *Escherichia coli Pyrf* mutations. Molec. General Genet. **198:** 179-182 (1984).
3. **Fonzi, W. A. and Irwin, M. Y.** 1993. Isogenic Strain Construction and Gene-Mapping in *Candida albicans*. Genetics **134:** 717-728.
4. **Wilson, R. B., Davis, D. and Mitchell, A. P.** 1999. Rapid hypothesis testing with *Candida albicans* through gene disruption with short homology regions. J. Bacteriol. **181**: 1868-1874.
5. **Murad, A. M. A., Lee, P. R., Broadbent, I. D., Barelle, C. J. and Brown, A. J. P.** 2000. CIp10, an efficient and convenient integrating vector for *Candida albicans*. Yeast **16**, 325-327.
6. **Dennison, P. M. J., Ramsdale, M., Manson, C. L. and Brown, A. J. P.** 2005. Gene disruption in *Candida albicans* using a synthetic, codon-optimised Cre-loxP system. Fungal Genet. Biol. **42:** 737-748.
7. **Noble, S. M. and Johnson, A. D.** 2005. Strains and strategies for large-scale gene deletion studies of the diploid human fungal pathogen *Candida albicans*. *Eu*karyotic Cell **4:** 298-309.
8. **Chiranand, W., Zhou, H., Lynn, J.J., Vega, L.A., Myers, H., Yates III, J.R., Lorenz, M.C. and Gustin, M.C.** 2008. CTA4 transcription factor mediates induction of nitrosative stress response in *Candida albicans*. Eukaryotic Cell. **7:** 268-278.
9. **Singh, P., Chauhan, N., Ghosh, A., Dixon, F., and Calderone, R.** 2004. SKN7 of *Candida* albicans: Mutant construction and phenotype analysis. Infect. Immun. **72**, 2390-2394 (2004).
10. **Frohner, I. E., Bourgeois, C., Yatsyk, K., Majer, O. and Kuchler, K.** 2009. *Candida albicans* cell surface superoxide dismutases degrade host-derived reactive oxygen species to escape innate immune surveillance. Molec. Microbiol. **71:**, 240-252.
11. **Dujon, B. *et al.*** 2004. Genome evolution in yeasts. Nature **430**: 35-44.
12. **Wood, V. *et al.*** 2002. The genome sequence of *Schizosaccharomyces pombe*. Nature **415:** 871-880.
