# Supplementary material for: Mechanisms Underlying the Exquisite Sensitivity of Candida albicans to Combinatorial Cationic and Oxidative Stress That Enhances the Potent Fungicidal Activity of Phagocytes
Source: mBio. 2014 Jul 15;5(4):e01334-14. doi: 10.1128/mBio.01334-14 (PMC4161263; doi:10.1128/mBio.01334-14)
Supplement: Table S2 — Microarray data set. [file mbo004141905st2.pdf]

Table S2. Microarray dataset

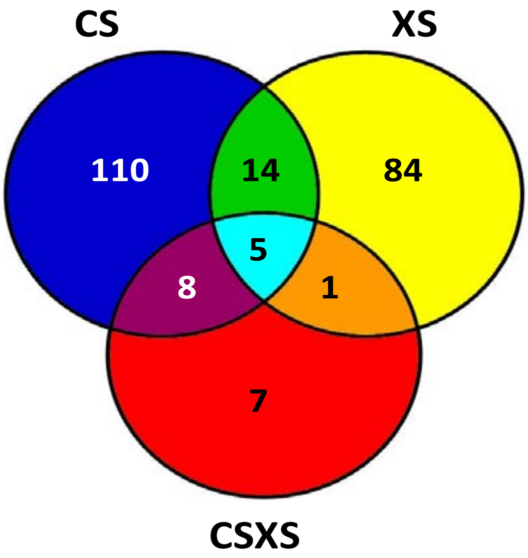

Numbers of UP-regulated *C. albicans* genes following CS (1M NaCl), XS (5 mM H_2O_2), or CSXS (1M NaCl + 5 mM H_2O_2) for 10 min. Genes upregulated >2.5 fold (p<0.05) are colourcoded according to this Venn diagram in the gene lists

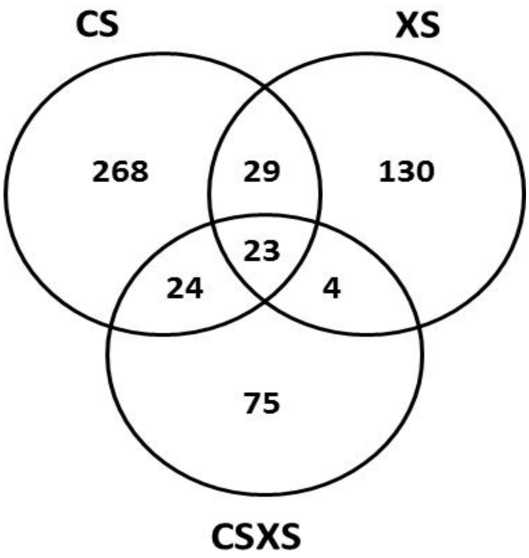

Numbers of DOWN-regulated *C. albicans* genes following CS (1M NaCl), XS (5 mM H_2O_2), or CSXS (1M NaCl + 5 mM H_2O_2) for 10 min. Genes upregulated >2.5 fold (p<0.05)

Gene Regulation (microarray dataset)

			Fold change	
orf 19	Gene	CS	XS	CSXS
orf19.5239		6.17	4.98	2.83
orf19.7003		37.69	0.93	14.09
orf19.6957.3		22.07	1.47	7.27
orf19.4155.12		19.70	1.42	6.71
orf19.6724	FUM12	6.32	1.75	4.50
orf19.5806	ALD5	7.64	0.68	3.74
orf19.3549	CDC21	2.70	1.84	2.68
orf19.7598		6.09	12.52	3.33
orf19.2896	SOU1	5.64	3.96	2.74
orf19.2959.1		3.55	3.28	2.31
orf19.5640	PEX5	4.05	2.69	2.20
orf19.1078	HBR2	5.21	6.20	2.10

orf19.3483		6.92	3.68	2.08
orf19.1375	LEU42	4.02	10.44	2.01
orf19.2244		3.73	5.21	1.92
orf19.359		2.81	7.31	1.90
orf19.449		2.81	4.02	1.83
orf19.3340	SOD2	2.69	3.93	1.75
orf19.2067		2.72	7.86	1.38
orf19.691	GPD2	3.17	2.63	1.28
orf19.6484		77.57	8.35	6.78
orf19.2756		5.60	2.78	3.33
orf19.4370		8.40	15.99	2.77
orf19.1743	ACS1	3.99	0.89	2.71
orf19.3218		3.16	2.62	3.29
orf19.346		5.27	1.48	2.73
orf19.6528		2.29	2.32	3.13
orf19.6630		2.79	1.40	3.35
orf19.6990		3.31	7.50	12.83
orf19.7284	ASR2	6.71	3.51	4.09
orf19.6548	ISU1	5.67	26.65	6.88
orf19.5521	ISA1	18.74	20.21	7.65
orf19.7296		36.48	6.71	26.10
orf19.2531	CSP37	3.51	3.34	3.38
orf19.5291		3.43	3.17	2.84
orf19.3355	ISN1	2.25	3.54	2.98
orf19.1125		1.11	61.36	1.12
orf19.3131	OYE32	1.49	56.42	1.47
orf19.6229	CAT1	4.08	38.02	1.79
orf19.4290	TRR1	1.31	18.57	1.26
orf19.5517		2.04	13.39	1.20
orf19.1162		1.54	13.04	1.15
orf19.2825		1.74	12.79	1.50
orf19.238	CCP1	2.41	11.72	1.88
orf19.1623	CAP1	1.94	11.53	1.13
orf19.2396	IFR2	3.07	10.93	2.77
orf19.861		7.85	10.87	8.67
orf19.3395		0.45	10.70	0.94
orf19.6059	TTR1	1.32	10.33	1.49
orf19.4147	GLR1	1.46	9.65	1.15
orf19.2461	PRN4	1.67	8.96	1.05
orf19.1861		1.51	7.56	1.35
orf19.7611	TRX1	1.44	7.08	1.58
orf19.251		1.28	7.00	1.12
orf19.4737	TPO3	0.62	6.53	0.81
orf19.4449		2.11	6.47	1.31
orf19.86		1.66	5.79	1.13
orf19.7085		3.27	5.72	1.99
orf19.3139		1.45	5.63	0.84
orf19.847	YIM1	0.84	5.35	0.64
orf19.1756	GPD1	0.30	5.27	0.50
orf19.473	TPO4	0.45	5.22	0.65
orf19.4907		0.93	5.08	0.94
orf19.6509		1.55	4.85	1.60
orf19.2073		1.22	4.83	0.94
orf19.7398.1	TSA1B	1.26	4.67	1.24
orf19.6811		1.88	4.61	1.61
orf19.2337	ALP1	1.05	4.30	0.83
orf19.7417	TSA1	1.21	4.29	1.20
orf19.411		0.80	4.27	0.84
orf19.2006.1		2.30	4.24	1.24
orf19.1189		0.62	4.23	0.53
orf19.5286	YCP4	1.52	4.15	1.48
orf19.388	CAF16	2.39	4.09	1.45
orf19.4754	ZWF1	1.38	3.97	0.87
orf19.4784	CRP1	0.77	3.97	1.09
orf19.2028	MXR1	2.49	3.92	1.73
orf19.3130		0.87	3.85	0.87
orf19.4309	GRP2	1.84	3.80	1.50

orf19.6608		1.78	3.78	2.27
orf19.6527		2.43	3.69	0.86
orf19.3026	MAS1	2.05	3.67	1.28
orf19.4371	TAL1	2.40	3.50	1.53
orf19.3644		2.24	3.44	1.15
orf19.2737		7.22	3.43	2.55
orf19.7558	YTA6	1.75	3.39	1.34
orf19.1290	XKS1	2.20	3.30	1.42
orf19.6063	UBP6	2.23	3.17	1.78
orf19.7570		1.89	3.16	1.35
orf19.4430		2.34	3.01	1.78
orf19.1153	GAD1	2.06	2.98	1.29
orf19.3448		1.05	2.97	1.36
orf19.749		1.07	2.97	1.30
orf19.868	ADAEC	2.76	2.93	2.27
orf19.6463		1.29	2.92	0.98
orf19.451	SOK1	0.93	2.92	0.74
orf19.7583		0.83	2.91	0.98
orf19.3054	RPN3	2.06	2.86	1.73
orf19.3195	HIP1	1.74	2.82	0.94
orf19.79		0.53	2.82	0.50
orf19.99	HAL21	1.48	2.78	1.67
orf19.2618	MET2	1.93	2.77	1.09
orf19.7438	UBA1	2.20	2.77	1.82
orf19.4127		2.33	2.77	1.90
orf19.6770		0.53	2.75	0.89
orf19.226		1.27	2.73	1.39
orf19.4246		2.70	2.71	1.88
orf19.3392	DOG1	2.11	2.70	1.97
orf19.7531		1.29	2.69	1.14
orf19.1816	ALS3	0.55	2.62	0.41
orf19.5193		1.47	2.62	1.95
orf19.4871	ERO1	1.10	2.61	1.00
orf19.5378		2.03	2.58	2.04
orf19.909	STP4	1.29	2.58	1.33
orf19.3099	TRP4	1.79	2.56	1.40
orf19.884		3.41	2.52	1.51
orf19.2333		1.70	2.52	1.74
orf19.4513		1.19	2.51	1.03
orf19.1517	ARO3	1.96	2.51	1.68
orf19.2344	ASR1	20.69	7.14	4.75
orf19.5984		8.72	1.11	5.88
orf19.7625	PGA1	6.78	1.10	2.73
orf19.5753	HGT10	6.21	2.35	1.74
orf19.7481	MDH1	5.84	0.89	2.43
orf19.5257		5.23	2.31	2.00
orf19.3184		4.96	3.15	2.99
orf19.5118	SDS24	4.93	1.66	2.18
orf19.7313	SSU1	4.87	3.11	3.04
orf19.348		4.66	0.86	1.56
orf19.5469		4.63	1.30	1.13
CaalfMr16	RRNL	4.61	1.78	2.13
orf19.5842		4.49	2.03	2.20
orf19.4393	CIT1	4.38	1.34	1.70
orf19.5474		4.25	2.49	1.61
orf19.5045		4.22	8.14	1.94
orf19.4985	GUP1	4.22	2.14	2.22
orf19.7350		4.20	1.41	2.04
orf19.1359		4.19	2.44	1.60
orf19.6475		4.18	1.59	2.26
orf19.3073		4.10	1.87	1.52
orf19.6661		4.05	1.40	3.30
orf19.1632		3.88	2.16	2.28
orf19.3508		3.86	1.36	1.79
orf19.1168		3.82	0.78	1.25
orf19.2270	SMF12	3.73	1.60	1.76
orf19.6385	ACO1	3.69	2.47	2.25

orf19.4316		3.66	1.33	1.68
orf19.2003	HNM1	3.62	0.79	1.28
orf19.2593	BIO2	3.61	1.20	1.73
orf19.6981		3.46	1.00	1.68
orf19.7323	CBP1	3.43	1.37	2.97
orf19.7229		3.34	1.90	1.86
orf19.5804	HYU1	3.34	1.88	2.37
CaalfMp07	ATP8	3.32	1.35	1.71
orf19.2787	PRY1	3.27	1.36	2.28
orf19.2844		3.26	1.29	1.86
orf19.4531		3.19	2.02	1.29
orf19.6086	LEU4	3.16	1.80	1.66
orf19.6126	KGD2	3.10	1.78	1.90
orf19.6460		3.06	1.41	2.24
CaalfMt37	TRND	3.06	1.81	3.06
orf19.620		3.04	1.70	3.16
orf19.448		3.01	2.13	1.78
orf19.1652	POX1-3	2.99	0.86	1.23
orf19.7247	RIM101	2.97	0.92	1.27
orf19.7286	RPN7	2.93	2.73	1.97
orf19.2172	ARA1	2.93	2.45	2.15
orf19.3135		2.92	1.71	2.14
orf19.637	SDH2	2.92	1.38	2.04
orf19.1670	BRO1	2.91	1.77	1.66
orf19.4535	PTR3	2.90	2.05	1.62
orf19.697		2.90	1.23	1.13
orf19.7589		2.90	2.24	2.33
orf19.3437		2.88	1.46	2.50
orf19.4540	UBC8	2.87	2.13	2.06
orf19.1012		2.84	1.32	2.14
orf19.3689		2.84	0.89	1.26
orf19.3898		2.82	1.08	2.16
orf19.1805	PEX14	2.80	1.89	2.01
orf19.1709		2.78	1.15	2.08
orf19.88	ILV5	2.78	0.77	2.38
orf19.4763		2.77	0.92	1.66
orf19.6306		2.77	1.41	2.41
orf19.2245		2.76	2.40	2.99
orf19.121	ARC18	2.75	1.91	4.38
orf19.7001	YCK2	2.75	1.16	1.56
orf19.3507	MCR1	2.74	1.31	1.91
orf19.1625		2.74	1.07	1.47
orf19.2933		2.74	1.63	1.97
orf19.7251	WSC4	2.73	1.07	1.47
orf19.3859		2.70	1.04	1.89
orf19.6756		2.69	1.81	2.03
orf19.298		2.68	1.02	1.71
orf19.2730		2.68	1.54	1.71
orf19.2568	IFU5	2.68	2.60	2.17
orf19.4102	RPN10	2.67	1.94	1.67
orf19.6180		2.67	1.30	2.17
orf19.6287	AAT21	2.67	0.90	1.07
orf19.5749	SBA1	2.66	1.90	2.05
orf19.6834.10	TAR1	2.65	1.08	2.18
orf19.3928		2.64	1.33	1.67
orf19.4953		2.64	1.54	1.34
orf19.6077		2.63	1.00	1.41
orf19.7258	DDI1	2.63	1.85	1.72
orf19.4579		2.63	0.84	2.36
orf19.3843		2.63	1.92	1.81
orf19.1338		2.62	1.66	1.72
orf19.5438		2.61	2.34	2.30
orf19.1159		2.61	1.06	1.69
orf19.1493	RAD7	2.61	2.24	1.67
orf19.7224		2.61	1.17	1.35
orf19.5419	ATP5	2.60	1.58	2.00
orf19.711		2.60	2.02	1.59

orf19.7498	LEU1	2.58	2.15	1.78
orf19.4059		2.58	1.67	1.50
orf19.7140		2.57	1.13	1.62
orf19.2792	IST2	2.57	1.26	1.55
orf19.6255		2.57	2.18	1.92
orf19.2181		2.56	1.06	1.76
orf19.1246		2.55	1.10	1.67
orf19.3782.2		2.55	1.40	1.98
orf19.2599		2.54	0.46	2.08
orf19.3401	CTA1	2.54	2.30	1.53
orf19.7343		2.53	1.67	1.54
orf19.4054	CTA24	2.53	1.79	2.19
orf19.6440		2.52	2.44	1.74
orf19.5757		2.51	2.57	1.79
orf19.3846	LYS4	2.50	2.67	1.28
orf19.20	RTS1	2.50	1.76	2.32
CaalfMp02	NAD6	2.23	1.19	1.63
CaalfMp04	COX3A	1.64	0.75	0.98
CaalfMt19	TRNN	1.50	0.91	1.88
CaalfMt39	TRNS2	1.79	0.94	1.83
orf19.1026		1.35	1.23	1.24
orf19.1042	POR1	1.57	1.10	1.31
orf19.1051	HTA2	0.92	0.09	0.93
orf19.1052		1.32	0.32	1.29
orf19.1054		2.05	0.54	1.50
orf19.1057		1.82	1.74	2.34
orf19.1059	HHF1	1.18	0.11	1.35
orf19.1061	HHT21	1.11	0.16	1.25
orf19.1064	ACS2	0.95	0.50	0.78
orf19.1082.1		1.53	1.02	1.63
orf19.1084	CDC39	1.51	1.29	1.19
orf19.1085		1.69	1.42	1.39
orf19.1086		1.55	1.15	1.24
orf19.1098		0.58	0.86	0.55
orf19.1099		1.97	1.16	2.29
orf19.1105.2	PGA56	1.51	0.34	1.14
orf19.1115	GUK1	1.07	0.66	1.04
orf19.1119	MTR10	1.46	1.23	1.06
orf19.1121		1.22	2.13	1.49
orf19.1140		2.31	1.29	1.58
orf19.1172		0.36	0.07	0.86
orf19.1179		1.56	1.15	1.35
orf19.118	FAD2	0.44	0.34	0.57
orf19.1187	CPH2	1.61	1.31	1.23
orf19.1191		2.07	1.58	1.91
orf19.1203.1		1.91	1.11	1.55
orf19.1210		1.55	1.45	1.73
orf19.1212		1.99	1.04	1.52
orf19.1232	VRG4	1.66	0.56	1.27
orf19.1240		2.00	1.67	1.35
orf19.1244	GYP2	1.96	0.94	1.71
orf19.1254	SEC23	1.81	1.02	1.54
orf19.1267		1.54	1.68	1.14
orf19.1273		1.37	0.87	1.51
orf19.1279	CDS1	1.75	1.10	1.84
orf19.1282		1.33	0.57	1.45
orf19.1299	RPN6	1.73	1.64	1.69
orf19.1352		2.22	1.04	1.60
orf19.1357	FCY21	0.48	0.87	0.47
orf19.1376		1.74	1.17	1.54
orf19.1389		1.58	1.08	1.12
orf19.139	TRA1	1.68	1.17	1.04
orf19.1390	PMI1	1.76	0.89	1.59
orf19.1394		1.88	1.56	1.15
orf19.1404		0.56	0.87	0.40
orf19.1467	COX13	1.73	0.91	1.48
orf19.1468		2.38	1.75	2.13

orf19.1471	COX4	2.07	1.03	1.69
orf19.1474	SLA1	1.92	1.30	1.44
orf19.1478		1.70	0.79	1.62
orf19.1486		2.11	1.54	1.85
orf19.1514		1.51	1.02	1.42
orf19.1531		1.79	0.97	1.33
orf19.1544		1.61	1.47	1.88
orf19.1552	CPR3	1.62	1.35	1.41
orf19.1553	ENT3	1.85	1.20	1.51
orf19.1559	HOM2	1.70	2.16	1.30
orf19.1560	POB3	1.61	1.40	1.24
orf19.1564		1.99	0.83	1.81
orf19.1570	ERG7	2.28	1.40	1.47
orf19.1595		1.48	1.15	0.96
orf19.1597	ABG1	2.44	0.61	1.66
orf19.1618	GFA1	1.89	0.91	1.25
orf19.1628	LAP41	2.33	1.38	1.63
orf19.1630		1.35	0.76	0.90
orf19.1631	ERG6	1.86	0.47	1.37
orf19.1642		0.94	0.75	0.39
orf19.1643		1.70	1.12	1.04
orf19.1649		1.57	1.55	1.77
orf19.1653		0.68	1.76	0.87
orf19.1654		1.16	2.24	1.35
orf19.1655.3		1.58	0.99	1.43
orf19.1658		2.45	1.48	1.44
orf19.1663	MNT2	0.98	0.45	0.98
orf19.1665	MNT1	1.84	0.58	1.55
orf19.1674		1.29	1.25	1.10
orf19.1680		2.12	0.82	1.56
orf19.1682		2.22	1.44	1.87
orf19.1683	PPH21	2.01	1.35	1.91
orf19.1690	TOS1	1.34	0.20	0.99
orf19.1700	RPS7A	1.15	0.67	1.05
orf19.1710		1.31	1.02	1.19
orf19.1721	NCE103	0.33	0.73	0.29
orf19.1727	PMC1	1.89	0.65	1.10
orf19.1738	UGP1	2.24	0.73	2.11
orf19.1750		1.27	1.18	1.20
orf19.1759	PHO23	2.37	1.42	1.52
orf19.1761		2.21	1.47	1.81
orf19.1779	MP65	1.20	0.22	0.86
orf19.1789.1	LYS1	1.83	1.32	1.21
orf19.1790		2.47	1.67	1.66
orf19.1792		2.04	1.16	1.41
orf19.1795	PUF3	1.11	1.08	1.61
orf19.1796		1.67	1.76	1.19
orf19.18	IMH3	0.97	0.54	0.78
orf19.1801		1.72	0.80	1.33
orf19.1814		1.74	1.54	1.48
orf19.1840		1.72	0.99	1.32
orf19.1852		1.86	1.34	1.62
orf19.1865		1.43	0.92	1.32
orf19.1866		1.77	1.48	1.78
orf19.1868	RNR22	0.37	2.02	0.96
orf19.1872		1.96	1.07	1.58
orf19.1873		1.74	1.57	2.11
orf19.1881		1.45	0.98	1.51
orf19.1885	TAF4	1.74	1.83	2.20
orf19.1886	RCL1	0.82	0.94	0.57
orf19.1891	Apr-01	2.04	0.91	1.40
orf19.1897		1.24	0.73	0.87
orf19.19		1.16	0.52	0.76
orf19.1902	NOC4	0.67	1.03	0.57
orf19.1903		2.12	1.36	1.09
orf19.1905		2.05	1.61	1.81
orf19.1917		2.40	1.34	1.59

orf19.1936	SNF1	2.00	1.33	1.31
orf19.1940		1.92	1.21	1.79
orf19.1946		1.39	1.24	1.42
orf19.1949	VPS1	2.02	1.27	1.63
orf19.1964		0.34	0.24	0.47
orf19.1978	GIT2	1.61	0.36	1.23
orf19.1986	ARO2	1.47	1.61	1.30
orf19.1989	DCW1	1.71	0.73	1.20
orf19.1991		1.84	1.05	1.32
orf19.1993		1.73	1.87	1.68
orf19.1996	CHA1	0.81	0.20	0.41
orf19.1997		0.82	0.31	0.72
orf19.200		2.00	2.08	1.74
orf19.2008		2.25	1.53	1.70
orf19.2013	KAR2	2.37	1.05	1.65
orf19.2029		2.47	1.62	1.71
orf19.2057		1.69	1.38	1.54
orf19.2070		1.87	1.05	1.35
orf19.2093	RFA1	1.64	0.94	1.80
orf19.2107	MUQ1	1.49	2.12	1.24
orf19.2113		2.26	0.68	1.58
orf19.2117		2.44	1.17	2.01
orf19.2119	NDT80	1.22	0.97	0.74
orf19.2121		0.53	0.63	0.42
orf19.213		2.23	2.42	2.25
orf19.2131		2.12	1.72	1.89
orf19.2132		1.27	1.82	1.46
orf19.2150		1.54	0.95	1.35
orf19.2156	NAG1	1.97	1.49	1.11
orf19.2163		1.69	0.92	1.36
orf19.2170	PHM7	1.75	0.48	1.13
orf19.2179	SIT1	0.12	0.19	0.15
orf19.2180		1.86	0.94	1.28
orf19.2182	BLM3	1.73	1.91	1.38
orf19.2184		2.40	1.28	1.99
orf19.2186		2.08	1.15	1.45
orf19.2193	PRS5	1.05	1.30	0.72
orf19.2233	PRE2	1.91	1.57	1.50
orf19.2241	PST1	2.29	1.74	1.55
orf19.2251	AAH1	0.14	1.22	0.18
orf19.2263		2.36	1.85	2.29
orf19.2267	RFA2	2.28	1.23	1.95
orf19.2276		1.78	0.91	1.67
orf19.2277	TPK2	1.84	1.14	1.76
orf19.2289	ARP3	1.85	1.68	1.34
orf19.229		2.02	1.84	1.54
orf19.2295		2.10	1.68	2.15
orf19.23	RTA3	1.90	1.16	1.24
orf19.2307		1.59	1.82	1.65
orf19.2308		2.35	2.34	1.20
orf19.231	APL2	1.86	1.03	1.14
orf19.2314		0.60	0.55	0.56
orf19.2322.3		1.84	1.32	2.02
orf19.2341		2.08	1.57	1.62
orf19.2353		2.14	0.82	1.10
orf19.236	RPL9B	1.09	0.56	0.95
orf19.2385	KTI12	0.83	0.78	0.53
orf19.239		1.33	0.99	1.18
orf19.2402	SSU72	2.06	1.75	1.29
orf19.242.2		2.11	1.25	1.82
orf19.2439.1		1.90	1.28	1.76
orf19.2444	CHS7	1.64	0.40	1.19
orf19.2459		1.40	1.04	1.35
orf19.2518		1.67	1.40	1.66
orf19.252		1.73	1.16	1.27
orf19.253		1.42	0.50	1.13
orf19.2533.1		1.62	0.44	1.40

orf19.2534	PIN4	1.81	1.22	1.29
orf19.2537		2.05	1.20	1.52
orf19.2546	TRP2	1.74	1.43	1.52
orf19.2571	SEC4	1.34	0.83	1.17
orf19.2582		1.70	1.46	1.13
orf19.2644	QCR2	2.41	1.25	1.63
orf19.2668	RHD2	1.38	1.69	1.58
orf19.2673		1.93	1.35	1.44
orf19.2685	PGA54	1.17	0.36	1.15
orf19.2688		0.55	0.94	0.44
orf19.2699	ABP1	2.24	1.69	1.53
orf19.2706	CRH11	1.72	0.73	1.36
orf19.2707.1	QCR9	1.46	0.88	1.43
orf19.2731		1.74	1.53	1.63
orf19.2746		1.87	0.46	1.33
orf19.2747	RGT1	1.41	0.97	0.93
orf19.2770.1	SOD1	2.47	1.91	1.94
orf19.2777		1.39	0.25	1.06
orf19.2778		1.73	1.37	1.22
orf19.2782		2.38	1.70	1.65
orf19.279		2.31	2.28	1.85
orf19.2821		1.95	1.35	1.58
orf19.2831	RPC31	1.44	1.35	1.17
orf19.284		1.50	0.91	1.36
orf19.2849	AQY1	0.28	0.99	0.26
orf19.285		2.34	2.03	1.72
orf19.287		1.34	0.86	1.33
orf19.2871	SDH12	2.15	0.61	1.52
orf19.2877	PDC11	0.76	0.65	0.76
orf19.2884	CDC68	2.23	1.43	1.28
orf19.2888		1.59	1.30	1.20
orf19.290	KRE5	1.75	1.17	1.54
orf19.2904		1.49	1.03	1.10
orf19.2909	ERG26	1.62	1.38	1.09
orf19.291		1.46	1.02	1.56
orf19.2937	PMM1	1.49	0.87	1.39
orf19.2942	DIP5	2.26	0.19	0.72
orf19.2951	HOM6	1.55	1.03	1.32
orf19.2964		1.96	0.70	1.24
orf19.2972	PDE2	0.90	1.85	0.50
orf19.2974	YKT6	1.82	1.64	1.80
orf19.2977		1.10	2.16	0.97
orf19.2985		2.46	2.19	2.45
orf19.2998	TSR2	0.96	0.74	0.69
orf19.3003		1.76	0.91	1.24
orf19.3006		1.99	1.64	1.34
orf19.3010.1	ECM33	1.61	0.42	1.31
orf19.3013	CDC12	1.70	1.19	1.22
orf19.3047		2.03	1.28	1.86
orf19.3052	YPT1	1.88	1.07	1.44
orf19.3060		1.91	0.90	1.69
orf19.3066	ENG1	0.97	0.30	1.13
orf19.3074	TLO10	1.92	1.30	1.32
orf19.3077	VID21	2.04	1.31	1.75
orf19.3089		1.40	0.77	0.74
orf19.3093	MSH2	2.25	1.07	1.51
orf19.3116	EXM2	2.00	1.16	1.37
orf19.3123	RPT5	1.94	1.72	1.33
orf19.3128		2.28	1.71	1.56
orf19.3136		2.09	1.12	1.67
orf19.3160	HSP12	2.19	1.60	1.78
orf19.3161		1.24	1.86	0.76
orf19.3168	RPN8	1.86	2.48	1.72
orf19.3169		1.59	1.58	1.17
orf19.3183		2.18	2.08	1.67
orf19.3191		1.50	0.82	0.82
orf19.3197	PAP1	2.27	1.18	1.43

orf19.3198	OBPA	1.88	1.12	1.42
orf19.3206		2.09	1.34	1.38
orf19.3215		2.14	1.10	1.81
orf19.3220		0.81	0.88	0.48
orf19.3223	ATP3	2.28	1.33	1.94
orf19.3224		2.42	1.42	1.75
orf19.3225		1.56	0.75	1.14
orf19.3237		2.12	1.02	1.60
orf19.3243	SRP54	2.02	1.52	1.39
orf19.3259		2.31	1.15	1.74
orf19.3260		1.97	2.32	1.38
orf19.3288	NMA111	0.70	0.69	0.62
orf19.3301		1.56	2.22	0.91
orf19.3307		2.23	2.50	2.15
orf19.3309		1.84	1.66	1.10
orf19.3319		1.61	1.14	1.41
orf19.3321		1.79	1.23	1.14
orf19.3322	DUT1	2.23	0.41	1.89
orf19.3335		2.33	0.93	1.31
orf19.337		1.83	1.03	1.40
orf19.3396	HCH1	1.55	1.79	1.54
orf19.3426	ANB1	1.38	0.98	1.21
orf19.3428		1.96	0.75	1.18
orf19.3441	FRP6	1.72	0.72	1.40
orf19.3447		1.58	2.20	1.33
orf19.3449.2		2.28	2.34	1.68
orf19.3458		2.07	1.13	1.62
orf19.3465	RPL10A	0.98	0.58	0.80
orf19.3467		1.41	1.24	1.32
orf19.3475		2.24	0.66	1.98
orf19.3496		1.65	1.30	1.42
orf19.3504	RPL23A	1.05	0.63	0.97
orf19.351		1.63	0.92	1.04
orf19.3528		1.62	1.06	1.67
orf19.3535		2.03	1.09	1.40
orf19.3542	LEM3	2.44	1.11	1.47
orf19.3546		1.69	1.12	1.37
orf19.3554	AAT1	1.82	0.84	1.50
orf19.3563		1.50	0.95	1.07
orf19.3575	CDC19	0.47	0.55	0.66
orf19.3593	RPT6	1.83	1.86	1.49
orf19.3628	RSP5	1.76	2.13	1.39
orf19.364		1.83	0.97	1.56
orf19.3642	SUN41	1.22	0.13	1.09
orf19.3649		1.26	1.73	1.01
orf19.3651	PGK1	0.64	0.66	0.83
orf19.3653	FAT1	1.63	1.07	1.63
orf19.366		1.83	1.31	1.44
orf19.3676	ABP140	0.93	0.62	0.97
orf19.3680	Sep-07	1.96	1.10	1.23
orf19.3683		1.78	1.55	1.29
orf19.3686		2.37	1.40	1.48
orf19.3688		2.25	1.37	2.09
orf19.3691		2.21	1.93	1.57
orf19.3694		1.39	0.69	1.16
orf19.37		1.69	1.45	0.78
orf19.3703		1.71	1.76	2.21
orf19.3710	YHB5	0.65	0.63	1.47
orf19.376		2.18	1.59	2.08
orf19.3764	GSG1	0.61	0.64	0.63
orf19.3765	RAX2	1.93	0.99	1.60
orf19.3767		1.67	1.48	1.10
orf19.3829	PHR1	1.32	0.32	0.84
orf19.384		1.97	1.04	1.28
orf19.3844	MRP8	2.36	1.11	1.78
orf19.385	GCV2	1.00	0.23	0.73
orf19.3861	SIS1	2.47	1.48	2.15

orf19.3869		0.67	0.20	0.65
orf19.3872		1.81	0.56	1.26
orf19.3873	ARC40	1.64	0.88	1.53
orf19.3884	FGR50	1.40	1.59	1.21
orf19.3889		1.64	0.78	1.52
orf19.3890		1.15	1.09	1.28
orf19.390	CDC42	1.64	0.61	1.58
orf19.3920		1.59	0.81	1.54
orf19.3930	YUH2	1.72	1.64	1.47
orf19.3934	CAR1	2.17	1.09	1.48
orf19.394		1.60	1.33	1.22
orf19.3944	GRR1	1.92	1.71	1.23
orf19.3967	PFK1	0.61	1.58	0.77
orf19.3974	PUT2	1.64	0.80	1.19
orf19.3978		0.57	1.23	0.52
orf19.399		1.09	1.84	1.01
orf19.3994		2.18	0.94	1.58
orf19.3997	ADH1	1.14	0.77	0.91
orf19.4009	CNB1	1.40	1.02	1.44
orf19.4010	PAN3	1.62	1.49	1.64
orf19.4016		1.87	1.01	1.30
orf19.4021		1.18	0.86	1.14
orf19.4023	MRP2	1.49	0.84	1.28
orf19.4024	RIB5	2.05	1.08	1.77
orf19.4028		2.22	1.87	1.50
orf19.4034		2.46	2.08	1.59
orf19.4046		1.50	0.61	0.85
orf19.4055		2.03	1.31	1.35
orf19.4056	GAT2	0.21	0.56	0.22
orf19.4076	MET10	1.33	0.77	1.15
orf19.4088		1.90	1.76	1.29
orf19.410.3		1.86	2.28	1.31
orf19.4107		2.05	1.13	1.12
orf19.4118	CNT	0.37	0.62	0.41
orf19.4123		2.14	1.30	1.63
orf19.4131		1.75	2.20	1.10
orf19.4144		1.84	1.65	1.57
orf19.415		2.33	1.17	1.53
orf19.4152	CEF3	1.01	0.62	0.82
orf19.4157		1.75	0.78	1.07
orf19.4173		0.91	0.77	0.57
orf19.4180		1.92	1.34	1.50
orf19.4181	SPC2	1.35	1.11	1.45
orf19.4184		1.49	1.61	1.51
orf19.4191.1		1.61	1.44	1.40
orf19.4197	YHM2	1.94	1.07	1.35
orf19.42		2.14	2.33	1.79
orf19.4201	NHX1	2.24	1.13	1.57
orf19.4215	FET34	0.79	0.34	0.57
orf19.4220		1.78	1.45	1.61
orf19.4228		1.49	0.86	1.28
orf19.4230		1.50	1.59	1.41
orf19.4236	RET2	2.15	1.16	1.44
orf19.4248		1.28	0.83	1.34
orf19.426		1.31	0.69	0.95
orf19.4271		1.59	1.31	1.52
orf19.4275	RAD9	1.95	1.51	1.86
orf19.4278		1.00	1.67	1.28
orf19.428		1.75	0.92	1.38
orf19.4304	GAP1	0.55	2.18	0.55
orf19.4317	GRE3	2.30	2.21	2.09
orf19.4339	VPS4	2.44	1.90	1.20
orf19.4340.1		1.81	1.63	1.29
orf19.4363	SGD1	1.80	1.78	1.15
orf19.4395		1.77	1.32	1.28
orf19.4406		2.05	2.33	2.31
orf19.441	RPT1	1.70	2.06	1.21

orf19.4416	VPS13	2.27	1.77	1.26
orf19.4421		1.55	1.59	1.86
orf19.4423		1.92	1.94	1.64
orf19.4445		1.25	2.34	0.45
orf19.4447	YMC1	1.30	0.82	1.20
orf19.4448	SOG2	1.37	1.95	1.08
orf19.4468		2.39	0.93	1.74
orf19.4469		1.70	1.85	1.51
orf19.4488		1.87	1.11	1.22
orf19.4502		1.19	1.09	1.36
orf19.4503		2.08	1.45	1.63
orf19.4516		1.94	1.66	1.19
orf19.4529		1.54	1.83	2.06
orf19.4555	ALS4	0.54	0.68	0.46
orf19.4575		2.12	1.23	1.60
orf19.4585	TFG1	1.41	1.12	1.20
orf19.4594		1.90	1.85	1.85
orf19.4597		2.23	2.18	2.39
orf19.4599	PHO89	0.54	0.38	0.82
orf19.4600.1		1.85	0.99	1.67
orf19.4602	MDH1-1	2.25	1.15	1.70
orf19.4603	ARL1	1.72	2.27	1.58
orf19.4609		1.75	1.32	1.63
orf19.4616	POL30	1.75	0.96	1.68
orf19.4618	FBA1	0.56	0.64	0.63
orf19.4626		1.98	1.11	1.16
orf19.4631	ERG251	1.48	1.01	1.55
orf19.4645	BEM1	1.70	1.31	1.84
orf19.4650	ILV6	1.67	1.26	1.43
orf19.4651	PGA53	1.30	0.93	1.33
orf19.4669	AAT22	1.78	0.97	1.63
orf19.4688	DAG7	0.80	0.38	0.84
orf19.4698	PTC8	1.91	2.16	0.94
orf19.4720	CTR2	2.00	1.30	1.35
orf19.4722		1.64	1.02	2.11
orf19.4728		1.68	1.81	1.50
orf19.4729		1.30	1.20	0.89
orf19.4739		0.49	0.79	0.43
orf19.4758		2.21	0.98	1.22
orf19.4759	COX5	2.19	1.35	1.70
orf19.4765	PGA6	1.09	0.14	1.09
orf19.4785	PTC1	2.25	1.31	1.61
orf19.479.2		2.22	1.63	1.81
orf19.4798		2.10	1.04	1.64
orf19.4829	DOA1	2.03	1.90	2.13
orf19.4831		1.64	0.69	1.43
orf19.4841	SHY1	1.43	1.44	1.68
orf19.4843		2.20	1.07	1.79
orf19.4851	TFA1	1.56	1.29	1.68
orf19.4878		2.50	1.45	1.88
orf19.4892	TPK1	0.67	2.19	0.65
orf19.4905		1.62	1.67	1.66
orf19.4906		0.85	1.01	0.76
orf19.491		2.26	0.89	1.85
orf19.4910	FGR41	1.29	0.57	0.83
orf19.492	ADE17	1.60	0.91	1.05
orf19.4930	SPC3	2.25	1.24	2.31
orf19.4933	FAD3	0.56	0.28	0.52
orf19.4945	MSH6	2.01	0.88	2.08
orf19.4952		1.53	0.59	0.97
orf19.4954		2.49	0.62	1.77
orf19.4956	RPN1	1.68	1.93	1.28
orf19.496		2.23	0.96	1.96
orf19.4961	STP2	2.46	1.44	1.48
orf19.4967	COX19	1.49	0.77	0.80
orf19.4969	KEM1	1.89	2.10	1.90
orf19.4994		2.43	1.99	2.35

orf19.5006.1		1.57	1.33	1.56
orf19.5013	AGM1	1.96	1.10	1.30
orf19.5019		1.59	0.75	1.82
orf19.5032	SIM1	0.80	0.27	0.65
orf19.504		1.43	1.59	1.05
orf19.5073	DPM1	1.76	1.23	1.55
orf19.5074		1.67	1.63	2.07
orf19.5077		1.54	0.91	1.15
orf19.5078		1.36	1.43	1.51
orf19.508	QDR1	0.29	0.43	0.48
orf19.5087	BUD6	2.24	1.36	1.48
orf19.5095		2.05	1.72	1.41
orf19.5101	CCR4	1.19	1.10	1.06
orf19.511		2.12	0.64	1.74
orf19.5112	TKL1	1.84	1.75	1.29
orf19.5130	PDI1	1.93	1.58	1.53
orf19.5137		0.99	0.22	0.82
orf19.5139		2.13	1.40	1.82
orf19.5141		1.30	0.65	1.04
orf19.5156		2.32	0.56	1.70
orf19.5175		1.49	1.73	1.21
orf19.5177		1.37	1.48	1.60
orf19.5183		1.55	1.26	1.36
orf19.5185		1.65	1.34	1.55
orf19.5188	CHS1	1.22	0.42	0.98
orf19.5194.1		1.97	2.04	1.68
orf19.52		1.52	1.24	1.17
orf19.5207		0.63	0.60	0.58
orf19.522		1.69	2.11	1.37
orf19.5225.2	RPL27A	1.22	0.76	1.18
orf19.5231.2		2.04	0.73	1.74
orf19.5260	RPN2	1.92	1.71	1.40
orf19.5261		1.66	0.92	0.85
orf19.5280	MUP1	0.88	0.69	0.45
orf19.5285	PST3	1.54	2.09	1.08
orf19.5287		1.26	0.96	1.34
orf19.5303	PGA30	1.60	0.66	1.26
orf19.5305	RHD3	0.63	1.99	0.67
orf19.5325	KIN3	1.62	1.30	1.45
orf19.5333		1.29	0.58	0.85
orf19.5338	GAL4	1.66	1.83	1.26
orf19.5345		2.32	1.79	1.70
orf19.5370		1.98	1.70	1.73
orf19.5379	ERG4	1.74	0.81	1.45
orf19.5395		1.94	1.56	2.01
orf19.5440	RPT2	1.87	2.09	1.62
orf19.5443		1.65	1.37	1.71
orf19.5445	GLO3	1.97	1.00	1.60
orf19.5446		2.11	1.09	1.60
orf19.5450		2.24	1.13	1.61
orf19.5457		2.09	0.84	1.56
orf19.5467	TLO7	2.05	1.62	2.06
orf19.547		2.26	1.01	1.70
orf19.548	CDC10	1.59	0.81	1.44
orf19.5484		1.73	1.14	1.26
orf19.5491.1	ATP14	1.91	1.09	1.56
orf19.5507	ENP1	0.33	0.67	0.30
orf19.551		1.13	0.74	0.73
orf19.5522		1.92	1.03	1.29
orf19.5531	CDC37	1.92	1.88	1.41
orf19.5544	SAC6	2.09	1.74	1.50
orf19.5546		1.89	1.91	1.46
orf19.556		1.39	0.67	1.49
orf19.5602	BMT6	1.89	0.71	1.68
orf19.5629	QCR7	1.58	1.39	1.71
orf19.5636	RBT5	1.26	0.30	0.79
orf19.5641	CAR2	2.14	0.96	1.32

orf19.5648		1.61	1.01	1.38
orf19.5653	ATP2	2.27	1.36	1.71
orf19.5654		1.99	1.05	1.68
orf19.566		1.49	1.15	0.94
orf19.5660.1		2.06	0.88	1.91
orf19.5666		2.11	2.11	2.04
orf19.5671		1.83	1.47	1.79
orf19.5674	PGA10	0.28	0.51	0.41
orf19.5676		1.80	1.63	1.35
orf19.5683		2.21	2.41	1.70
orf19.5688		1.85	1.54	1.76
orf19.5689		1.51	1.20	1.47
orf19.5700	TLO11	2.15	1.54	1.71
orf19.5733		0.47	1.08	0.39
orf19.5741	ALS1	0.45	2.45	0.35
orf19.575	HYR3	1.95	1.33	1.33
orf19.5754		1.18	1.96	0.80
orf19.5758	SAL6	0.81	1.98	0.78
orf19.5769		1.90	2.19	1.57
orf19.577		1.95	1.20	1.46
orf19.5772		2.16	1.16	2.15
orf19.5776	TOM1	1.59	1.70	1.12
orf19.579	FOL1	2.28	2.10	1.85
orf19.5793	PR26	1.67	1.35	1.15
orf19.5811	MET1	2.44	1.76	1.73
orf19.5823	SGT2	1.96	1.20	1.52
orf19.5846		1.31	2.25	1.25
orf19.5849	CWT1	1.97	1.57	1.72
orf19.5884		1.18	0.43	0.67
orf19.5885		0.88	0.74	0.77
orf19.589		1.56	1.51	1.16
orf19.5890		1.62	2.15	1.63
orf19.59	REI1	0.34	0.97	0.26
orf19.590		1.92	2.06	2.33
orf19.5901	PKC1	1.91	1.23	1.83
orf19.5908	TEC1	0.63	0.53	0.41
orf19.5912	MAK21	0.79	0.70	0.55
orf19.592		2.40	1.27	2.14
orf19.5920		2.29	1.20	1.56
orf19.593	FGR32	1.72	1.29	1.53
orf19.5941		2.18	0.93	1.57
orf19.5949	FAS2	0.61	0.42	0.52
orf19.5951		0.60	0.36	0.45
orf19.5953		2.17	1.15	1.20
orf19.5960		1.81	0.64	1.27
orf19.5965		2.35	2.08	1.79
orf19.5968	RDI1	1.76	1.07	1.40
orf19.5989		1.23	1.36	0.94
orf19.5999	DYN1	1.77	1.17	1.15
orf19.6007		1.21	0.28	0.67
orf19.6010	CDC5	0.88	0.26	0.71
orf19.6026	ERG2	1.20	0.48	1.23
orf19.6028	HGC1	2.27	1.49	1.39
orf19.6034	TUB2	1.70	0.56	1.66
orf19.6040	SNF7	1.78	2.47	1.91
orf19.6045		1.73	1.26	2.00
orf19.6058	GLO1	1.75	2.49	1.56
orf19.6062		1.52	0.94	1.35
orf19.6066		2.25	0.71	1.25
orf19.6070	ENA2	1.73	0.55	1.09
orf19.6073	HMX1	0.93	0.30	0.49
orf19.6078.1		1.86	1.38	1.48
orf19.6082		1.76	1.38	1.44
orf19.6092	KEL1	1.56	1.01	1.59
orf19.6094		1.84	1.11	1.16
orf19.6109	TUP1	1.55	0.80	1.15
orf19.6132		2.02	1.19	2.14

orf19.6135.1	SMX4	1.19	1.31	1.47
orf19.6151	ARC15	1.60	1.14	1.36
orf19.6153		2.38	2.23	1.95
orf19.6155		2.00	1.51	2.05
orf19.6163	CSE4	2.42	0.86	1.48
orf19.6165	KGD1	2.09	1.37	1.19
orf19.6167	AYR1	2.33	0.90	1.83
orf19.6169.2		2.32	1.05	1.68
orf19.6176	SEC61	2.17	0.78	1.93
orf19.6197		1.83	1.55	1.50
orf19.6202	RBT4	0.76	0.13	0.70
orf19.6245		2.04	2.25	1.03
orf19.6250		1.85	1.24	1.43
orf19.6252		1.94	1.82	1.33
orf19.6263		1.20	1.19	1.34
orf19.6264.3		2.19	0.73	1.44
orf19.6268		1.99	1.21	1.22
orf19.6272		2.34	1.73	1.63
orf19.6305		2.00	1.25	1.41
orf19.6309		1.86	1.55	1.11
orf19.631		2.17	1.66	1.87
orf19.6321	PGA48	1.20	0.72	1.33
orf19.6324	VID27	2.40	1.17	1.88
orf19.6326		1.58	0.45	0.93
orf19.6365	PTP1	0.61	0.53	0.60
orf19.6373		1.62	1.43	1.17
orf19.6374		2.04	1.14	1.48
orf19.6377		1.56	1.20	1.51
orf19.6400		1.76	1.80	1.33
orf19.6402	CYS3	0.77	2.14	0.49
orf19.6415.1		0.97	0.51	0.86
orf19.6435		2.43	1.25	1.95
orf19.645.1		2.49	1.38	1.97
orf19.646	GLN1	1.01	0.19	0.85
orf19.6461		1.59	1.07	1.02
orf19.6470	AHP2	2.39	1.60	1.79
orf19.6472	CYP1	1.31	0.77	1.12
orf19.6478	YCF1	1.23	2.40	1.04
orf19.649		1.84	1.00	1.12
orf19.6494	WHI3	1.59	1.00	1.66
orf19.6534.2		1.95	1.41	1.59
orf19.6538	VMA11	2.31	0.68	1.70
orf19.6544	LPI9	1.84	1.94	1.58
orf19.655	PHO84	0.51	0.10	1.23
orf19.6552		1.99	0.97	1.93
orf19.6553		1.55	0.88	1.28
orf19.6561	LAT1	1.34	0.90	0.94
orf19.657	SAM2	0.96	0.51	0.80
orf19.6588		1.66	1.14	1.21
orf19.6596		1.10	1.67	0.92
orf19.6601.1	YKE2	1.82	0.87	1.16
orf19.6607		1.37	0.82	0.98
orf19.6634	VMA2	1.73	0.88	1.47
orf19.6642		2.16	1.14	1.40
orf19.6659	GAP6	1.20	2.50	0.50
orf19.6673	HEX1	1.21	0.67	0.98
orf19.6710		0.77	0.92	0.59
orf19.6720		1.63	1.17	1.21
orf19.6736		0.59	0.67	0.51
orf19.6745	TPI1	0.82	0.97	0.81
orf19.6747		2.15	2.13	1.51
orf19.6753		1.79	2.09	1.47
orf19.676		2.48	0.60	1.77
orf19.6787		1.34	0.29	1.27
orf19.6798	SSN6	2.12	1.26	1.14
orf19.6809		1.75	1.97	1.11
orf19.6810		2.03	1.25	1.52

orf19.6817	FCR1	1.31	2.33	1.24
orf19.6835		2.50	0.85	1.84
orf19.6837		1.70	2.43	1.74
orf19.6843		1.90	1.11	1.26
orf19.6863	VPH1	1.74	0.99	1.22
orf19.6867		1.82	0.99	1.61
orf19.6874		0.65	0.41	0.75
orf19.6880		2.00	1.42	1.24
orf19.6883		1.99	1.57	1.20
orf19.6887		1.67	0.81	1.43
orf19.6902		0.67	1.11	0.50
orf19.6906	ASC1	1.22	0.50	0.94
orf19.6915	MRE11	1.96	1.86	2.20
orf19.6924	HTA1	1.03	0.43	1.13
orf19.6927	PEP8	1.91	2.05	1.40
orf19.6928	SAP9	1.36	2.29	1.22
orf19.6933		1.84	2.07	1.96
orf19.6944	PHB1	1.83	0.87	1.12
orf19.6948	CCC1	2.22	2.29	1.61
orf19.6953	IRS4	1.71	1.93	1.54
orf19.6968		1.03	0.32	0.71
orf19.6979		1.99	1.22	1.41
orf19.6987	DNM1	2.01	1.57	1.45
orf19.6991	PRE3	2.21	1.92	2.02
orf19.7030	SSR1	1.64	1.04	1.40
orf19.7035		2.26	1.21	2.03
orf19.7048		1.32	0.87	1.34
orf19.7050	NOP15	0.81	0.68	0.66
orf19.7058		1.64	1.10	1.68
orf19.706	NMD3	0.65	1.41	0.48
orf19.7086		1.38	1.35	1.45
orf19.709	PUP2	1.85	1.50	1.25
orf19.7092		1.92	1.65	1.42
orf19.7095		2.25	1.25	1.73
orf19.71		2.02	1.50	1.66
orf19.7100		2.37	1.20	1.57
orf19.7109		1.29	0.88	1.38
orf19.7115	SAC7	1.90	0.80	1.35
orf19.7116		1.90	1.23	1.33
orf19.7118		1.80	1.78	1.92
orf19.7119		1.85	1.17	1.31
orf19.7124		1.99	1.17	1.35
orf19.7127	TLO16	1.95	1.80	2.14
orf19.7144		1.78	1.46	1.23
orf19.7161	SUI3	1.64	1.39	1.33
orf19.717	HSP60	1.54	0.55	0.95
orf19.7186	CLB4	1.78	0.94	2.14
orf19.7196		2.42	0.91	1.68
orf19.7201	SLA2	1.93	1.35	1.17
orf19.7214		2.21	1.12	1.46
orf19.7215		0.65	0.85	0.55
orf19.7215.3		1.47	0.79	1.37
orf19.7218	RBE1	0.76	0.15	1.12
orf19.7222		1.62	1.61	1.16
orf19.7234		1.62	0.95	1.32
orf19.7239		1.84	1.11	1.47
orf19.7261		2.27	1.20	1.82
orf19.7263		1.78	1.05	1.21
orf19.7265		1.79	1.42	1.48
orf19.7290		1.92	1.01	1.41
orf19.7292	ARP2	2.03	1.35	1.57
orf19.7307		1.00	0.38	0.78
orf19.7328		1.92	1.53	1.51
orf19.7362	SKN1	0.91	0.34	0.73
orf19.7382	CAM1	1.06	0.57	0.94
orf19.7398		0.71	0.84	0.54
orf19.7402		1.26	0.63	1.68

orf19.7424	NSA2	0.60	0.85	0.58
orf19.7428	APN1	1.59	1.48	1.74
orf19.745	VAC8	1.64	1.28	1.75
orf19.7458		1.50	1.83	1.56
orf19.7459		0.51	0.83	1.01
orf19.7499		1.70	1.01	1.16
orf19.7501		1.59	1.96	1.13
orf19.7510	KIN2	1.69	1.34	1.14
orf19.7522		2.10	0.96	1.39
orf19.7529	EPL1	2.07	1.81	1.34
orf19.758		2.29	0.89	1.66
orf19.759		1.85	1.24	1.53
orf19.7597	PGA12	1.63	1.24	1.53
orf19.7600	FDH3	0.98	2.19	0.77
orf19.7603		1.35	1.52	1.44
orf19.7605	PUP1	1.75	2.13	1.48
orf19.7610	PTP3	1.14	2.28	0.88
orf19.7612	CTM1	1.07	2.36	1.07
orf19.7635	DRS1	0.87	0.87	0.61
orf19.7644		2.17	1.45	2.07
orf19.7645		2.08	0.59	1.49
orf19.7654	CPR6	2.20	1.34	1.90
orf19.7667	IAH1	1.97	1.25	1.37
orf19.767	ERG3	0.53	0.37	0.65
orf19.784		1.54	0.97	1.78
orf19.792		1.54	1.26	1.07
orf19.835		1.73	2.05	1.36
orf19.836.1		1.23	1.05	1.46
orf19.837	GNA1	1.42	1.20	1.37
orf19.841		2.26	1.07	1.73
orf19.843		1.61	1.22	1.72
orf19.875		1.27	1.13	1.21
orf19.903	GPM1	0.59	0.75	0.70
orf19.905	AVT7	2.09	1.00	1.90
orf19.911		1.40	1.71	1.06
orf19.916		2.08	1.03	1.38
orf19.921		2.36	1.56	1.45
orf19.925		2.48	0.87	1.51
orf19.929		1.50	0.88	1.07
orf19.94		1.99	1.19	1.57
orf19.942	KRE62	2.02	0.98	1.63
orf19.945	VPS2	2.41	1.75	1.27
orf19.954		1.42	1.05	1.19
orf19.979	FAS1	0.48	0.38	0.40
orf19.986	GLY1	0.52	2.24	0.52
orf19.997		1.97	1.28	1.56
CaalfMp01	COX2	1.47	1.08	1.61
CaalfMp03	NAD1	2.14	0.91	1.71
CaalfMp05	ATP9	1.11	0.67	1.06
CaalfMp06	ATP6	1.81	0.90	1.42
CaalfMp09	NAD2	1.20	0.86	1.02
CaalfMp10	NAD3	1.32	0.84	1.15
CaalfMp12	NAD4L	1.79	0.84	1.27
CaalfMp13	NAD5	1.99	1.23	1.87
CaalfMp14	NAD4	1.34	0.76	1.64
CaalfMr17	RRNS	1.76	1.03	1.70
CaalfMt18	TRNA	1.31	0.78	1.50
CaalfMt22	TRNY1	2.39	0.65	1.57
CaalfMt23	TRNH1	1.56	0.54	1.22
CaalfMt24	TRNT1	6.62	0.85	4.64
CaalfMt32	TRNW	3.26	1.72	3.41
CaalfMt33	TRNF	1.84	1.12	1.59
CaalfMt35	TRNM1	1.48	0.94	1.43
CaalfMt38	TRNS1	1.80	0.84	1.57
CaalfMt40	TRNL2	1.67	0.90	1.42
CaalfMt44	TRNH2	4.17	1.59	3.72

CaalfMt47	TRNK2	5.08	1.40	2.92
orf19.1016		1.00	0.90	0.90
orf19.1018		1.71	1.02	1.30
orf19.1030		1.28	0.97	0.94
orf19.1031	HMG1	1.15	0.48	0.97
orf19.1033	STR2	1.45	1.50	1.01
orf19.1034		0.77	1.86	1.13
orf19.1047	ERB1	0.96	1.05	0.95
orf19.1048	IFD6	2.68	1.50	1.79
orf19.105	HAL22	2.13	2.04	1.68
orf19.1053		1.44	0.72	1.06
orf19.1055	CDC3	1.79	0.76	1.64
orf19.1058		2.23	2.65	1.62
orf19.1063		1.32	1.35	1.75
orf19.1067	GPM2	0.98	3.17	1.06
orf19.107		0.72	0.91	0.77
orf19.109		2.02	1.30	1.83
orf19.1091		0.55	1.12	0.59
orf19.1093	FLO8	1.22	0.48	1.21
orf19.1095		1.43	0.98	0.93
orf19.1097	ALS2	0.62	0.94	0.56
orf19.1101		1.69	1.41	1.31
orf19.1107		2.16	1.29	1.75
orf19.1108	HAM1	1.21	1.38	0.81
orf19.1109		1.12	0.76	0.93
orf19.1112	BUD7	1.65	0.93	1.16
orf19.1114		1.79	0.87	1.17
orf19.1124.2		1.09	0.73	0.79
orf19.1133	MSB1	1.46	0.69	1.12
orf19.1137		2.46	2.75	1.18
orf19.1139		1.12	0.84	0.72
orf19.1144		1.58	0.88	1.20
orf19.1150		2.17	1.55	2.24
orf19.1151		1.34	1.01	1.28
orf19.1152		1.48	0.87	1.21
orf19.1154	EGD1	1.39	0.83	1.25
orf19.1160		1.89	1.13	2.05
orf19.1161	PLD1	1.44	0.96	0.90
orf19.1164	GAR1	1.12	0.92	0.90
orf19.1166	CTA3	1.60	1.12	1.37
orf19.117		2.64	2.80	1.17
orf19.1170	ARO7	1.72	0.64	1.23
orf19.1174		0.69	0.80	0.55
orf19.1177		1.70	0.99	1.59
orf19.1183		2.12	1.18	1.23
orf19.1185		1.48	0.97	1.18
orf19.1193	GNP1	0.96	1.10	0.68
orf19.1195		1.50	0.85	1.17
orf19.1199	NOP5	0.85	0.74	0.67
orf19.1200		1.76	1.73	1.87
orf19.1204		2.05	0.96	1.36
orf19.1206	FET35	0.68	0.43	0.62
orf19.1214		1.15	1.38	0.77
orf19.1217		1.16	0.85	0.81
orf19.1219		1.51	1.41	1.61
orf19.122	CDC20	1.40	0.60	1.01
orf19.1220	RVS167	1.63	1.14	1.17
orf19.1222		1.25	0.76	1.28
orf19.1226		1.20	1.34	1.04
orf19.1230		1.01	0.86	0.62
orf19.1231		1.13	1.31	1.01
orf19.1233	ADE4	1.04	0.66	0.61
orf19.1235	HOM3	0.80	3.96	1.04
orf19.1248		1.70	1.09	1.47
orf19.1249		2.41	1.42	1.49
orf19.1250		1.09	1.07	1.09
orf19.1252	YME1	1.43	2.19	1.09

orf19.1257		1.25	1.19	0.96
orf19.1259		1.45	1.33	1.19
orf19.1267.1		1.70	2.26	1.33
orf19.1281		1.41	0.55	1.28
orf19.1283	MEC1	1.68	1.07	1.69
orf19.1287		0.91	0.78	0.99
orf19.1289	SCT1	1.19	0.95	1.28
orf19.1295	VAS1	1.47	1.10	1.16
orf19.13		1.01	1.27	1.14
orf19.1306		0.93	0.53	1.01
orf19.131.2		1.20	0.54	1.61
orf19.132		1.81	4.05	1.51
orf19.1321	HWP1	1.81	1.12	1.14
orf19.1333	SNG3	1.36	1.23	1.06
orf19.1335		0.74	0.58	0.61
orf19.1336	PUP3	1.43	1.46	1.39
orf19.1339	CPY1	1.68	1.11	1.49
orf19.1342	SHM1	1.20	0.69	0.89
orf19.1343		1.62	0.75	1.21
orf19.1353		0.08	1.48	0.23
orf19.1354	UCF1	0.06	2.28	0.14
orf19.1355		1.49	0.96	1.26
orf19.1358	GCN4	0.99	1.42	0.88
orf19.1361		1.14	0.84	0.97
orf19.1372		1.52	0.72	1.48
orf19.1378	SUP35	1.02	0.89	0.85
orf19.1386		1.59	1.06	1.32
orf19.1387		2.03	2.00	1.87
orf19.1388		0.50	0.91	0.58
orf19.1395		1.51	0.94	1.38
orf19.14		1.26	1.10	1.27
orf19.1402		1.59	1.08	1.28
orf19.1403		1.26	0.93	0.81
orf19.1407		0.89	1.71	1.41
orf19.1413	YFH1	1.88	0.81	1.52
orf19.1414		1.67	0.96	1.34
orf19.1435	TEF1	1.07	0.72	1.17
orf19.1444		2.62	1.81	1.48
orf19.1446	CLB2	1.70	0.63	1.48
orf19.1448	APT1	1.06	0.59	0.98
orf19.146		1.57	1.91	1.87
orf19.1460		1.33	1.63	1.13
orf19.1466		1.15	1.02	1.76
orf19.1470	RPS26A	1.07	0.74	1.08
orf19.1479		1.46	1.64	1.32
orf19.1480		1.81	0.84	1.37
orf19.1490	MSB2	1.40	0.69	1.17
orf19.1494	RAD23	1.26	0.94	1.01
orf19.150		0.90	0.95	0.77
orf19.1500		2.98	1.33	1.88
orf19.1505		4.40	1.33	1.78
orf19.1509	ROD1	1.32	1.70	0.90
orf19.1526	SNF2	1.33	0.87	1.23
orf19.1528		0.80	1.10	0.84
orf19.153		1.63	1.36	1.14
orf19.1532		1.18	1.38	1.41
orf19.1533		1.25	0.82	1.51
orf19.1536		1.79	0.82	1.32
orf19.1537		1.22	0.65	0.93
orf19.154		0.59	1.36	0.58
orf19.1545		0.87	1.10	1.08
orf19.1546		1.05	0.74	0.86
orf19.1549		1.89	1.28	1.72
orf19.155	URE2	1.57	1.25	0.90
orf19.1563	ECM3	1.61	1.31	1.51
orf19.1565		1.06	0.71	0.63
orf19.1566		0.76	0.64	0.56

orf19.1569	UTP22	0.83	0.72	0.54
orf19.157		1.49	0.96	1.17
orf19.1573		1.48	1.00	1.68
orf19.1574		1.15	0.87	0.85
orf19.1575	PRS1	0.68	0.78	0.63
orf19.1578		0.78	0.80	0.51
orf19.158		0.72	0.82	0.65
orf19.1585	ZRT2	1.54	0.55	2.84
orf19.1586	FGR22	1.97	2.75	2.00
orf19.1588		2.57	2.08	1.68
orf19.1591	ERG10	1.18	0.61	0.80
orf19.1592		3.27	2.14	1.85
orf19.1598	ERG24	1.63	0.63	1.27
orf19.1601	RPL3	1.09	0.67	0.93
orf19.1604		0.93	0.85	0.81
orf19.1607	ALR1	0.54	1.03	0.55
orf19.1613	ILV2	1.03	1.58	0.85
orf19.1624		2.01	1.34	1.38
orf19.1626		0.99	1.13	0.91
orf19.163		1.02	0.96	1.09
orf19.1633		0.78	0.75	0.54
orf19.1635	RPL12	1.44	0.74	1.12
orf19.1637		2.48	1.10	3.14
orf19.1646		0.82	0.97	0.68
orf19.1650		1.06	0.69	1.16
orf19.1661		1.52	1.00	1.26
orf19.1662		1.25	1.27	1.04
orf19.1669	AFG3	2.11	1.36	1.39
orf19.1671	UTR2	1.72	0.78	1.28
orf19.1672		1.80	1.31	1.36
orf19.1673	PPT1	0.99	0.60	0.81
orf19.1675		2.01	0.69	1.19
orf19.1677		1.31	0.87	1.02
orf19.1678		1.11	0.74	0.97
orf19.168		0.78	1.10	0.90
orf19.1687		0.56	1.07	0.61
orf19.1691		0.26	0.13	0.72
orf19.1693	CAS4	2.20	0.81	1.58
orf19.1694		1.84	0.90	1.64
orf19.1697		1.17	1.26	1.04
orf19.170		0.51	0.69	0.46
orf19.1706	MET18	1.60	1.11	1.19
orf19.1707		1.17	0.95	0.89
orf19.1708		1.18	1.30	1.19
orf19.171	DBP2	0.53	0.76	0.43
orf19.1730		2.01	1.98	2.17
orf19.1732		0.69	1.06	0.79
orf19.1733		1.68	0.89	1.37
orf19.1734		1.61	2.53	0.99
orf19.1738.1		1.30	0.90	1.50
orf19.1744	HEM4	1.79	1.39	2.33
orf19.1751	SPT23	1.06	1.14	0.91
orf19.1753	PUS7	0.80	1.09	0.60
orf19.1760	RAS1	1.61	1.36	1.43
orf19.1770	CYC1	1.84	0.80	1.66
orf19.1772		0.58	0.93	0.80
orf19.1777		2.08	1.73	1.49
orf19.1783	YOR1	1.16	1.88	1.06
orf19.1785		2.98	2.74	2.40
orf19.1786		1.32	0.88	1.11
orf19.1787		1.21	1.11	1.10
orf19.1788		1.53	1.27	0.92
orf19.1791		0.70	1.12	0.58
orf19.1799		0.94	1.28	0.84
orf19.1802		0.72	1.43	0.80
orf19.1804		1.59	1.47	1.23
orf19.1806		1.44	0.63	1.44

orf19.1815		0.86	0.87	0.66
orf19.1832	FCY23	2.09	0.82	1.08
orf19.1833		1.11	0.95	0.81
orf19.1835		1.37	1.16	1.67
orf19.1837	TBP1	1.31	0.79	1.10
orf19.1847	ARO10	3.67	1.04	1.76
orf19.185		1.23	1.22	1.12
orf19.1857		1.45	0.98	1.16
orf19.1860	LSC2	2.04	1.08	1.73
orf19.1860.1		1.99	0.98	1.69
orf19.1862		2.10	6.12	1.68
orf19.1864		1.73	1.78	1.32
orf19.1871		1.50	1.38	1.33
orf19.1880	HEM15	1.35	2.53	1.31
orf19.1889		1.12	1.18	1.26
orf19.1896	SSC1	1.32	0.83	0.91
orf19.1901		1.07	0.80	1.05
orf19.1907	EMC9	1.56	0.86	0.91
orf19.1910		2.52	1.13	1.87
orf19.1911	PGA52	1.18	0.81	1.11
orf19.1913		1.33	0.96	1.22
orf19.1915	MPP10	0.91	0.94	0.86
orf19.1923	RRN3	0.73	0.92	0.82
orf19.1925	TLO5	1.79	1.20	1.44
orf19.1942	SGE1	4.89	3.08	2.30
orf19.1944	GPR1	0.99	1.06	1.09
orf19.1952		1.54	1.78	1.26
orf19.1953		1.23	1.22	1.67
orf19.1956		1.03	0.70	0.81
orf19.1957	CYC3	0.89	1.06	0.53
orf19.1959		1.47	1.09	1.03
orf19.1961		1.47	0.93	0.93
orf19.1963		1.36	0.94	1.01
orf19.1966	BUD23	0.87	0.69	0.69
orf19.1967		1.16	0.69	0.95
orf19.1970		2.04	1.05	1.62
orf19.1972		1.75	0.99	1.24
orf19.1974	TFS1	2.03	1.64	1.82
orf19.1977		1.27	0.84	1.23
orf19.198	ASN1	1.26	1.03	1.11
orf19.1981		2.54	1.28	2.96
orf19.1982		1.04	0.32	1.15
orf19.1988	CDC45	1.96	1.19	1.38
orf19.199		1.77	0.85	2.18
orf19.2002		1.34	1.08	0.88
orf19.2005	REG1	3.24	1.26	2.80
orf19.2007		2.72	1.13	1.42
orf19.2012	NOT3	1.77	1.24	1.54
orf19.2014	BCY1	2.37	1.68	1.65
orf19.2015		2.31	1.49	1.70
orf19.2016		1.34	0.69	1.07
orf19.2017		1.13	0.90	0.84
orf19.2019		1.20	1.02	1.10
orf19.202	CDC47	1.62	0.69	1.30
orf19.2020	HGT6	1.46	0.44	1.56
orf19.2021	HGT8	1.12	1.29	1.08
orf19.2022		1.21	1.27	1.06
orf19.2023	HGT7	1.25	0.85	1.01
orf19.2039		1.95	1.20	1.04
orf19.2040		1.46	0.99	1.17
orf19.2041		1.44	1.21	1.18
orf19.2043		1.06	1.63	1.13
orf19.2050		3.38	3.07	1.94
orf19.2055	NPL6	1.47	1.12	1.21
orf19.2063		1.94	1.34	1.66
orf19.2069	SMF3	1.20	1.38	1.72
orf19.2075	DFG5	1.75	1.20	1.12

orf19.2076		1.73	1.03	1.79
orf19.2078		3.04	2.03	1.93
orf19.2081		1.33	0.87	1.29
orf19.2088		1.91	1.36	1.16
orf19.2090		0.52	0.63	0.46
orf19.2092		2.36	2.98	1.52
orf19.2098	ARO8	1.63	2.99	1.66
orf19.2099		1.85	1.42	1.30
orf19.2101		1.21	1.10	1.51
orf19.2102	CKB1	1.26	1.27	1.00
orf19.2107.1	STF2	0.53	4.36	0.80
orf19.2110		2.89	1.04	1.17
orf19.2111.2	RPL38	0.88	0.66	1.16
orf19.2112		2.37	0.98	1.50
orf19.2115		1.18	0.60	0.81
orf19.2116	NAT2	1.51	1.09	1.08
orf19.2124		2.22	0.96	1.90
orf19.2128		1.46	0.97	0.99
orf19.2135	TSM1	1.56	1.12	1.39
orf19.2138	ILS1	1.02	0.96	0.86
orf19.2143		0.89	0.86	0.84
orf19.2146		2.41	1.10	5.50
orf19.2151	NAG6	2.12	1.30	1.65
orf19.2154	HXK1	1.40	1.34	1.27
orf19.2158	NAG3	2.70	1.01	2.07
orf19.2167		0.74	1.93	0.68
orf19.2168.3		1.81	0.54	1.34
orf19.2175		1.43	2.23	1.37
orf19.2176	IFM3	4.22	1.65	1.54
orf19.2179.2	RPS10	1.16	0.63	1.06
orf19.2183	KRE30	1.07	0.72	0.87
orf19.2192	GDH2	3.07	3.04	1.25
orf19.2197		2.73	1.69	2.65
orf19.220	PIR1	0.96	1.16	0.78
orf19.2200		1.84	0.79	1.67
orf19.2201		0.80	0.58	0.81
orf19.2208		1.16	1.57	0.94
orf19.2209	YVC1	1.61	0.66	1.09
orf19.2214		1.43	0.76	1.05
orf19.2215	GLE1	1.80	1.20	1.19
orf19.2216		2.00	1.71	2.21
orf19.2219	ORF298	1.33	2.61	1.12
orf19.2221		1.16	0.82	0.89
orf19.2232	RPL11	1.07	0.60	1.03
orf19.2236		0.75	0.53	0.61
orf19.2246		1.43	0.98	1.22
orf19.2248	ARE2	0.88	2.26	0.83
orf19.2250		1.17	0.89	0.91
orf19.2256		1.25	0.91	0.96
orf19.2257		1.94	1.07	1.57
orf19.2259		1.20	1.15	1.11
orf19.2260		0.89	1.52	1.61
orf19.2261		0.80	1.34	0.94
orf19.2264	OLE2	1.21	1.04	0.88
orf19.2265		1.32	1.17	1.36
orf19.2268	RCK2	1.53	2.03	0.89
orf19.2269		1.69	1.18	1.58
orf19.227	COX7	1.17	0.71	1.01
orf19.2275		0.84	0.54	0.86
orf19.2278		1.99	1.09	1.58
orf19.2283	DQD1	1.60	2.79	1.43
orf19.2286		0.90	1.02	0.85
orf19.2287	RPA12	0.81	0.96	0.76
orf19.2288		1.13	0.74	1.00
orf19.2294.1		1.97	1.34	1.28
orf19.2296		2.64	1.44	1.41
orf19.2297	ARL3	3.35	1.08	1.30

orf19.2298		1.56	0.84	1.37
orf19.2304		2.11	1.24	1.57
orf19.2309.2		1.50	0.92	1.15
orf19.2310.1	RPL29	0.92	0.48	0.84
orf19.2311	RPL82	0.99	1.10	0.92
orf19.2318.1		1.53	0.51	1.34
orf19.2319		0.73	0.79	0.52
orf19.2324	UBA4	0.76	1.89	0.72
orf19.2328		2.24	0.88	1.31
orf19.2329.1	RPS17B	1.04	0.64	0.95
orf19.233.1	HRT1	2.17	1.40	1.52
orf19.2330		1.45	0.90	1.18
orf19.2335		0.99	1.43	0.89
orf19.2340	CDC48	1.70	1.32	1.29
orf19.2347	MNN2	1.34	1.03	1.87
orf19.2351	NIT3	1.88	3.17	1.84
orf19.2355	ALS10	1.66	1.15	1.04
orf19.2358		1.30	1.85	1.06
orf19.2360	URA2	0.99	0.74	0.68
orf19.2363		1.17	0.94	0.96
orf19.2364	MIS11	1.36	0.77	0.91
orf19.2365	POL2	1.17	0.98	1.21
orf19.2368		1.10	1.35	1.20
orf19.2369.1	ATX1	1.38	5.23	1.72
orf19.2371		1.70	2.23	1.34
orf19.2372		2.13	3.62	1.63
orf19.2374		2.19	2.87	1.80
orf19.2376		1.46	1.18	1.27
orf19.2378		1.47	0.90	1.58
orf19.2379	NOT4	1.72	1.51	1.42
orf19.2382		1.63	0.88	1.54
orf19.2386		0.99	1.48	0.63
orf19.2387		0.96	0.54	0.91
orf19.2392		0.82	1.59	0.86
orf19.2398		2.14	1.55	1.62
orf19.240		1.19	0.88	1.07
orf19.2400		1.71	1.39	0.84
orf19.2404		1.13	1.03	0.74
orf19.2407	DPS1-1	1.38	1.02	1.07
orf19.241		1.44	1.25	1.32
orf19.2418		1.58	0.97	1.60
orf19.2419		1.27	1.19	1.08
orf19.2422	ARC1	1.47	1.34	1.09
orf19.2425.2		1.27	0.88	1.18
orf19.2426		2.55	0.90	1.34
orf19.2435	MSI3	1.49	1.08	0.98
orf19.2437	ARC35	1.99	1.12	1.60
orf19.2438		1.07	0.74	0.73
orf19.2439		2.13	1.12	1.37
orf19.2451	PGA45	0.75	0.73	1.12
orf19.2452		0.56	0.61	0.86
orf19.2454	PHO87	0.82	1.34	1.22
orf19.247		2.22	1.37	1.92
orf19.2471	GIM5	1.40	1.06	1.54
orf19.2473		1.15	0.86	1.13
orf19.2478.1		1.26	0.82	1.21
orf19.2483		1.25	0.68	1.17
orf19.2484		1.72	0.92	1.41
orf19.2485		1.04	0.73	0.70
orf19.2487		1.93	1.25	1.31
orf19.2488	FAL1	0.78	1.26	0.67
orf19.2489		1.16	0.74	0.79
orf19.2496		2.36	1.16	1.82
orf19.25		1.06	1.28	0.73
orf19.250	SLC1	1.12	0.69	0.95
orf19.2500		2.12	4.03	2.25
orf19.2501	FLC1	1.18	0.87	1.49

orf19.2504	BMS1	1.14	1.09	1.09
orf19.2507	ARP9	1.58	1.16	1.71
orf19.2511.1	MRPL33	1.53	0.80	1.21
orf19.2512		1.11	0.93	0.76
orf19.2514		1.54	1.39	1.19
orf19.2520		1.34	0.89	1.25
orf19.2524	MGE1	1.16	1.14	1.00
orf19.2525	LYS12	1.20	0.83	1.12
orf19.2527		0.76	0.62	0.76
orf19.2528		1.40	0.99	0.85
orf19.2538	PTC2	1.18	1.16	0.83
orf19.2544		1.51	1.04	1.38
orf19.2547		0.61	0.58	0.56
orf19.2550		1.79	2.15	1.77
orf19.2551	MET6	1.37	0.82	1.02
orf19.2555	URA5	1.13	1.16	1.28
orf19.2557	SEC65	1.46	1.36	1.42
orf19.2559	CDC4	1.12	0.98	1.02
orf19.2560	CDC60	1.32	1.22	1.16
orf19.2562	CDC60B	1.47	1.14	1.08
orf19.2563		1.17	0.94	1.60
orf19.2564		1.42	1.03	0.91
orf19.2570		1.82	1.42	1.66
orf19.2594		0.75	0.63	0.69
orf19.2598	VMA4	1.65	1.19	1.38
orf19.260	SLD1	0.86	0.88	1.08
orf19.2601	HEM1	0.45	1.73	0.81
orf19.2604		0.81	1.01	0.38
orf19.2606	HDA1	1.30	0.97	1.08
orf19.2608	ADH5	0.95	1.26	1.17
orf19.2611	MCM6	1.98	1.47	1.49
orf19.2613	ECM4	1.48	2.45	0.93
orf19.2622	YPT31	1.91	1.20	1.51
orf19.2631		1.10	1.14	0.79
orf19.2632		0.64	0.94	0.60
orf19.2634		1.51	1.21	1.12
orf19.2639		0.82	0.86	0.75
orf19.2639.1		1.84	1.24	1.65
orf19.2640	FUR1	1.19	0.93	0.96
orf19.2643		1.04	0.86	0.87
orf19.2650		1.48	1.36	0.80
orf19.2650.1		1.14	0.63	1.02
orf19.2651	CAM1-1	1.55	0.85	1.08
orf19.2658		1.86	1.17	1.60
orf19.2661	TLO34	1.84	1.13	1.20
orf19.2662		1.45	2.06	1.73
orf19.2664		2.09	1.14	1.09
orf19.2665	MSN5	1.61	2.01	1.50
orf19.2666		1.48	1.64	1.35
orf19.2667	RPF1	0.74	1.11	0.80
orf19.2669		1.57	1.50	1.15
orf19.267		1.40	1.03	1.46
orf19.2672	NCP1	1.37	0.95	1.15
orf19.2676		0.86	0.73	0.99
orf19.2677		2.38	0.80	1.47
orf19.2680		1.72	0.82	1.46
orf19.2682		1.91	1.77	1.68
orf19.2687.1		1.10	1.11	0.97
orf19.269	SES1	1.45	0.97	1.05
orf19.2690		1.15	1.26	1.23
orf19.2694	TYS1	1.08	0.76	0.88
orf19.2695	UBR1	1.64	1.68	1.30
orf19.2697		1.78	1.10	0.86
orf19.2709	ZUO1	1.29	1.22	1.07
orf19.2710		1.70	2.08	1.87
orf19.2711		0.80	0.65	0.58
orf19.2712	HCA4	0.87	1.08	0.48

orf19.2717	SAS10	0.92	1.21	0.80
orf19.2720		1.50	1.15	1.14
orf19.2721		1.83	5.63	2.31
orf19.2727	GRX3	1.74	1.02	1.47
orf19.273		1.30	0.78	1.06
orf19.2735	SEN2	0.61	0.52	0.68
orf19.2736		1.06	0.36	0.72
orf19.2749		2.22	2.57	1.19
orf19.2755		1.56	1.07	1.29
orf19.2757		1.52	1.85	1.26
orf19.2758	PGA38	0.66	0.67	0.87
orf19.2759		0.98	0.93	1.05
orf19.2760		1.29	1.48	0.99
orf19.2761		3.84	1.10	1.72
orf19.2762	AHP1	0.85	1.29	1.00
orf19.2765	PGA62	1.21	0.66	1.26
orf19.2767	PGA59	1.15	0.71	1.01
orf19.2769		2.15	2.83	1.80
orf19.2772	HOS3	2.10	3.07	2.80
orf19.2774		0.97	0.70	0.81
orf19.2775	IDI1	1.55	0.63	1.24
orf19.2785	ATP7	1.68	1.15	1.52
orf19.2786		2.70	1.10	2.25
orf19.2790		1.57	0.77	1.26
orf19.2794		1.10	0.88	2.34
orf19.2795		1.18	0.96	0.79
orf19.280		2.39	1.13	1.67
orf19.2801		2.03	3.12	1.84
orf19.2803	HEM13	0.40	1.09	0.87
orf19.2815		1.17	1.10	1.20
orf19.2820		1.59	1.72	1.29
orf19.2823	RFG1	1.50	0.96	0.95
orf19.2824		1.28	0.91	1.07
orf19.2826		1.59	1.09	1.41
orf19.2830	RRP9	0.55	0.99	0.41
orf19.2834	RPD3	1.25	1.55	1.13
orf19.2835		1.44	1.78	1.32
orf19.2841	PGM2	1.56	1.70	1.37
orf19.2843	RHO1	1.45	0.55	1.31
orf19.2847		1.14	0.95	0.67
orf19.2850		2.10	1.30	1.31
orf19.2857		1.88	1.21	1.15
orf19.2859	SRP40	0.88	0.64	0.56
orf19.2862	RIB1	2.30	5.94	1.39
orf19.2866		1.20	1.62	1.55
orf19.2873	TOP2	1.81	0.81	1.33
orf19.2881	MNN4	0.94	1.10	0.81
orf19.2889		1.42	1.33	1.34
orf19.2901	NUP60	1.33	0.96	1.18
orf19.2907.1		1.73	1.21	1.25
orf19.2915		3.25	0.86	2.01
orf19.2917		0.81	0.95	0.64
orf19.2920		1.22	1.19	1.25
orf19.2924		1.85	0.98	1.17
orf19.2926		2.09	1.84	1.99
orf19.2927	MNN11	0.90	0.79	0.77
orf19.2928		1.68	0.57	1.74
orf19.2929	GSC1	1.34	0.57	0.96
orf19.2930		1.30	0.78	1.08
orf19.2934		1.01	0.73	0.66
orf19.2935	RPL10	1.14	0.86	0.98
orf19.2938		1.58	1.10	1.60
orf19.2943		3.33	0.54	1.53
orf19.2947	SNZ1	2.21	1.06	1.36
orf19.2953		0.92	0.84	0.97
orf19.2954		2.95	1.39	2.10
orf19.2956	MGM101	1.97	1.02	1.48

orf19.2960	FRS2	1.39	1.09	1.09
orf19.2965		1.32	0.90	1.07
orf19.2966		1.55	1.19	1.66
orf19.2967	TIF34	1.36	0.96	1.09
orf19.2969	RAD16	0.72	2.17	0.85
orf19.2970	LYS2	2.07	3.01	1.53
orf19.2980		1.35	1.03	1.59
orf19.2983	CDC73	1.67	1.36	1.45
orf19.2987		1.32	0.89	1.01
orf19.299	ECM14	1.51	1.00	1.10
orf19.2990	XOG1	0.93	0.22	0.87
orf19.2992	RPP1A	1.29	0.80	1.25
orf19.2994	RPL13	1.30	0.93	1.12
orf19.2994.1	RPS16A	1.20	0.70	1.17
orf19.30		1.49	0.61	1.23
orf19.300	AIP2	1.71	0.78	1.40
orf19.3002	RPS1	1.21	0.83	1.14
orf19.3003.1	RPL6	1.16	0.90	1.24
orf19.3004		1.47	0.89	1.17
orf19.3008	COQ4	1.99	1.38	1.71
orf19.3015	ARX1	0.70	0.89	0.63
orf19.3016		1.45	1.21	1.13
orf19.3022		1.57	0.73	1.05
orf19.3034	RLI1	0.80	0.73	0.71
orf19.3035		1.39	1.15	1.30
orf19.3037		1.13	1.09	0.93
orf19.3038	TPS2	1.64	2.67	1.88
orf19.304		1.21	0.61	0.90
orf19.3040	EHT1	1.50	1.26	0.85
orf19.3045		1.84	1.16	1.61
orf19.3051		1.28	0.59	1.57
orf19.3053		1.03	2.35	1.23
orf19.3057		1.92	0.93	1.35
orf19.3058		1.58	1.14	1.38
orf19.3059	SUA71	0.97	1.72	0.68
orf19.3063		4.25	1.26	1.49
orf19.3064	MRPL27	1.08	0.74	1.19
orf19.308		1.41	0.62	0.91
orf19.3084		4.36	1.38	2.35
orf19.3086		2.50	0.90	1.63
orf19.3087	UBI3	1.03	0.64	1.08
orf19.3088		0.88	0.55	0.88
orf19.3091		1.03	0.90	0.95
orf19.3097	PDA1	1.22	0.68	0.90
orf19.3098		0.99	1.05	1.05
orf19.3102	CTA6	2.42	1.14	2.03
orf19.3103		1.12	1.06	0.73
orf19.3104	YDC1	1.95	3.25	1.58
orf19.3106	MET16	1.51	0.82	1.21
orf19.3120		1.35	1.31	2.56
orf19.3122.2		1.43	1.31	1.21
orf19.3124		0.91	1.29	0.69
orf19.3125		0.78	0.82	1.08
orf19.3126	CCT6	1.25	0.86	0.89
orf19.3127	CZF1	1.43	1.20	0.48
orf19.3129		1.26	1.12	1.03
orf19.3133	GUT2	0.74	1.76	0.57
orf19.3138	NOP1	0.73	0.53	0.49
orf19.3140.1		1.82	1.92	1.69
orf19.3141		0.84	1.00	1.11
orf19.3144		3.01	1.07	2.37
orf19.3145.4		0.82	0.76	0.95
orf19.3149	LSP1	1.37	1.01	1.09
orf19.3158		1.95	1.93	1.49
orf19.3159	UTP20	0.66	0.84	0.50
orf19.316	SEC13	2.38	1.98	2.01
orf19.3166		1.50	1.12	1.08

orf19.3167		1.09	0.99	0.92
orf19.317		1.56	1.32	1.38
orf19.3170		1.14	1.76	0.83
orf19.3171	ACH1	2.73	0.71	1.32
orf19.3174	CDC24	1.53	1.19	1.71
orf19.3175		1.56	1.84	1.03
orf19.3177		1.49	0.80	0.96
orf19.3178		1.44	1.72	1.67
orf19.3182	GIS2	1.39	0.66	1.09
orf19.3185		1.41	0.59	0.75
orf19.3192	STI1	1.94	1.08	1.55
orf19.3205		1.19	0.62	0.98
orf19.3207	CCN1	1.67	1.26	1.16
orf19.3211	RCF3	2.73	1.52	1.31
orf19.3222		1.95	0.83	2.23
orf19.3227	FTH2	1.49	0.95	0.95
orf19.3228		1.73	1.32	1.28
orf19.3230	BOI2	1.30	1.20	1.32
orf19.3235		1.66	1.55	1.53
orf19.3240	ERG27	1.76	0.66	1.25
orf19.3242		2.20	1.79	2.02
orf19.3244		1.63	0.87	1.49
orf19.3247		1.56	1.20	1.74
orf19.3249	LAG1	2.55	0.80	1.39
orf19.3251	ARC19	1.73	1.42	1.47
orf19.3256	SLN1	1.34	1.92	1.22
orf19.326		1.84	2.13	2.70
orf19.3262		1.42	0.62	0.86
orf19.3265	TRM1	1.61	1.13	0.99
orf19.3268		1.24	0.50	1.16
orf19.327	HTA3	1.05	0.46	0.93
orf19.3275		1.03	0.79	1.05
orf19.3276	PWP2	0.68	0.73	0.52
orf19.3278	GSY1	0.95	1.01	1.04
orf19.328	NPR2	1.27	0.96	1.23
orf19.329		1.40	1.37	1.59
orf19.3290		2.30	1.50	1.75
orf19.3291	HMT1	0.89	0.68	0.69
orf19.3293		2.08	0.65	1.65
orf19.3294	MBF1	1.60	1.37	1.40
orf19.3297		1.06	0.88	1.07
orf19.3298	CCH1	1.48	1.22	1.21
orf19.33		0.98	1.09	1.09
orf19.330.1		1.25	0.80	1.04
orf19.3300	ZPR1	1.06	1.24	0.86
orf19.3303		0.59	1.06	0.59
orf19.3304		1.05	1.39	0.76
orf19.331		1.62	1.08	1.34
orf19.3310		2.34	0.96	1.74
orf19.3312		2.19	1.31	1.48
orf19.3324	TIF	1.44	0.88	1.06
orf19.3325		1.42	1.15	1.34
orf19.3325.3	RPS21B	1.06	0.66	1.12
orf19.3327	TRM2	0.74	0.38	0.38
orf19.333	FCY2	0.76	1.22	1.06
orf19.3331	ABC1	1.07	1.48	0.77
orf19.3333		1.38	1.04	0.80
orf19.3334	RPS21	1.13	0.57	0.98
orf19.334		1.90	2.59	2.14
orf19.3341		1.23	1.17	0.99
orf19.3344	VPS17	2.49	4.18	5.50
orf19.3345	SIZ1	1.07	0.82	0.87
orf19.3348		1.26	0.99	1.24
orf19.3349		1.37	0.90	0.93
orf19.3350	MRP20	1.02	0.83	0.97
orf19.3354		1.05	0.60	0.82
orf19.3357		1.18	1.06	1.04

orf19.3358	LSC1	1.72	0.75	1.17
orf19.3359	ARP8	2.18	2.85	1.67
orf19.336	YAH1	1.11	2.32	0.99
orf19.3363	VTC4	0.82	0.38	0.96
orf19.3365	DAO2	1.24	1.39	1.30
orf19.3366	CSH3	1.83	0.97	1.44
orf19.3366.1		1.41	0.93	1.07
orf19.3367		1.10	0.92	1.06
orf19.3370	DOT4	0.64	0.83	0.70
orf19.338		1.58	0.70	0.86
orf19.3385		1.46	1.30	1.19
orf19.3386		2.08	1.32	1.69
orf19.339	NDE1	0.76	1.11	0.68
orf19.3390		1.59	1.72	0.99
orf19.3391	ADK1	1.04	0.89	0.91
orf19.3393		0.76	1.15	0.68
orf19.3394		2.00	1.37	1.49
orf19.3402		2.11	2.09	1.34
orf19.3409	SEC12	2.24	1.04	1.37
orf19.3415	PTK2	1.91	1.13	1.89
orf19.3415.1	RPL32	1.08	0.67	1.13
orf19.3419	MAE1	2.70	1.17	2.14
orf19.3422	FMP27	1.74	1.22	1.48
orf19.3423	TIF3	1.25	0.99	1.02
orf19.3429	FGR47	1.10	1.16	0.86
orf19.3431		1.52	1.05	1.04
orf19.3438		2.04	1.00	1.43
orf19.3442		0.84	1.39	1.14
orf19.3445	HOC1	1.50	0.95	1.02
orf19.3450.1		2.66	1.51	1.66
orf19.3451		1.68	1.08	1.25
orf19.3457		1.45	0.88	1.40
orf19.3462	SAR1	1.34	0.92	1.44
orf19.3463		0.81	1.72	0.64
orf19.3470		1.28	0.70	1.01
orf19.3473		1.86	1.42	2.26
orf19.3476		1.65	1.36	1.29
orf19.3477		0.71	0.79	0.42
orf19.3479		1.23	1.38	0.93
orf19.3480		1.01	0.68	0.75
orf19.3482		1.74	3.18	1.56
orf19.349		1.37	0.90	1.13
orf19.3498		2.48	1.26	1.56
orf19.350	PRE9	1.39	1.15	1.13
orf19.3505		3.80	1.12	1.61
orf19.3521	ARH2	5.19	2.19	1.24
orf19.3523	CRK1	1.47	1.28	1.41
orf19.3526	ITR1	3.12	1.33	2.16
orf19.3527	CYT1	1.45	0.87	1.27
orf19.353	ULP1	0.81	1.18	0.88
orf19.3530	CKA2	1.01	0.99	0.69
orf19.3532	MRPL10	1.43	0.98	1.26
orf19.3533		1.23	1.00	2.29
orf19.3534	RHO3	1.56	0.84	1.34
orf19.3539		1.13	1.06	1.21
orf19.354		0.80	1.34	1.29
orf19.3540	MAK5	0.63	0.90	0.48
orf19.3541	ERF1	1.05	0.64	0.78
orf19.3547		0.81	0.74	0.56
orf19.3548		6.08	1.15	1.37
orf19.3548.1	WH11	4.28	1.90	1.88
orf19.3552		1.71	1.00	1.38
orf19.3553	RPF2	0.81	0.88	0.83
orf19.3556		1.10	0.70	0.83
orf19.3558		1.71	0.57	1.78
orf19.3560		1.22	0.96	0.77
orf19.3564		0.66	0.69	0.49

orf19.3569		1.46	1.01	1.53
orf19.3572.3		0.99	0.61	0.87
orf19.3577	COQ5	1.16	0.85	1.01
orf19.3579	ATP4	1.54	0.95	1.14
orf19.3581		1.38	0.83	1.09
orf19.3582		1.21	1.02	1.16
orf19.3583		1.49	1.90	1.35
orf19.3585		1.13	0.71	0.81
orf19.3590	IPP1	1.28	0.88	1.20
orf19.3591	APE3	1.90	0.94	1.25
orf19.3599	TIF4631	1.46	1.04	0.92
orf19.3603		0.79	0.87	0.86
orf19.3608		0.84	0.86	1.14
orf19.3609	UTP15	0.90	0.73	0.82
orf19.3611		1.81	0.93	1.28
orf19.3616	ERG9	1.30	0.88	1.05
orf19.3617		2.90	1.10	1.49
orf19.3618	YWP1	1.35	0.28	1.25
orf19.362	TLO9	1.71	1.01	1.25
orf19.3622	ANP1	2.07	0.85	1.18
orf19.3623	SMC2	1.49	1.16	1.46
orf19.3624		0.69	0.59	0.83
orf19.3627		2.76	1.47	2.81
orf19.3629	DSE1	1.70	1.18	1.20
orf19.3630	RRP8	0.68	1.02	0.65
orf19.3641		1.01	1.27	1.29
orf19.3646	CTR1	1.07	1.24	0.98
orf19.3656	COX15	0.64	0.93	0.93
orf19.3658		0.92	0.68	0.86
orf19.3663	PHO91	1.38	1.35	0.98
orf19.3669	SHA3	1.45	0.86	1.31
orf19.367	CNH1	1.50	0.86	1.52
orf19.3681		1.27	0.80	1.07
orf19.3682	CWH8	1.14	0.32	0.97
orf19.3690		6.92	0.55	1.59
orf19.3690.2		1.08	0.55	0.90
orf19.3695		1.15	0.31	1.01
orf19.3696	TOM22	1.23	0.83	1.23
orf19.3697		2.93	1.80	2.24
orf19.3700	TOM70	1.18	0.99	0.98
orf19.3702		1.25	0.82	0.99
orf19.3704.1		1.07	0.82	1.18
orf19.3706		1.51	1.46	0.85
orf19.3707	YHB1	1.16	1.46	1.85
orf19.3712		0.77	0.69	0.43
orf19.3713		0.81	0.88	0.80
orf19.3722		1.28	1.23	1.13
orf19.3732	ERG25	2.44	0.35	1.21
orf19.3737		3.07	0.98	3.24
orf19.374		1.81	0.84	1.17
orf19.3751		0.83	0.18	0.86
orf19.3755		0.82	0.67	0.79
orf19.3756	CHR1	0.66	0.97	0.73
orf19.3757	ATP20	1.26	0.59	0.98
orf19.3772		1.77	1.47	1.38
orf19.3775	SSK2	1.41	0.79	0.79
orf19.3776		1.38	0.84	1.05
orf19.3777		0.78	0.39	0.76
orf19.3778		0.66	0.80	0.77
orf19.3779		1.05	0.69	0.62
orf19.3787		1.55	1.14	1.24
orf19.3788.1	RPL30	1.03	0.66	1.00
orf19.3789	RPL24A	1.05	0.48	0.73
orf19.379		1.14	1.30	0.51
orf19.3791	FGR10	1.98	1.01	1.35
orf19.3792		1.62	1.21	1.25
orf19.3796		0.93	1.29	0.96

orf19.3797		0.93	0.69	0.86
orf19.3798		0.96	1.05	0.93
orf19.3799		1.48	0.37	1.22
orf19.3802	PMT6	1.92	0.88	2.52
orf19.3803	MNN22	0.96	0.78	1.44
orf19.3804		1.70	0.60	1.30
orf19.3809		1.87	1.03	1.17
orf19.381		2.64	0.65	1.27
orf19.3810		0.67	0.40	0.49
orf19.3812	SSZ1	1.33	0.85	0.88
orf19.3818		2.43	1.19	1.51
orf19.3820		0.80	1.04	0.82
orf19.3822	SCS7	1.05	0.80	1.09
orf19.3823	ZDS1	1.25	0.86	1.37
orf19.3825	RCE1	1.25	1.06	1.25
orf19.3826		1.56	2.19	1.72
orf19.3833		1.46	1.36	1.33
orf19.3835		1.45	1.01	1.22
orf19.3836		0.99	0.67	1.03
orf19.3838	EFB1	1.43	0.73	1.09
orf19.3840		1.04	0.95	1.20
orf19.3845	FGR3	1.94	1.63	1.65
orf19.3856	CDC28	1.61	1.08	1.85
orf19.3858		4.07	2.07	1.62
orf19.3862		6.16	1.98	2.09
orf19.3867	RPL7	0.84	1.22	0.67
orf19.387	GCR3	1.47	1.27	0.98
orf19.3870	ADE13	1.14	0.66	0.80
orf19.3881		2.00	2.20	1.17
orf19.3887		0.64	1.87	0.83
orf19.3888	PGI1	0.82	0.69	0.74
orf19.3893	SCW11	0.73	0.33	0.90
orf19.3894		1.48	0.94	1.37
orf19.3895	CHT2	2.27	0.59	2.01
orf19.3900		5.56	1.50	1.50
orf19.3904		1.69	0.88	1.30
orf19.391	UPC2	0.58	1.09	1.08
orf19.3911	SAH1	1.13	0.80	0.84
orf19.3914		0.67	0.72	0.56
orf19.3915		1.34	0.94	1.06
orf19.3919		1.84	0.94	1.58
orf19.392		1.16	0.96	1.11
orf19.3921		1.14	0.92	1.01
orf19.3922		1.76	3.15	1.36
orf19.393		1.35	1.42	1.22
orf19.3938		1.24	0.99	1.03
orf19.3940.1	CUP1	1.03	0.87	1.24
orf19.3941	URA7	1.03	0.77	0.77
orf19.3942		1.97	1.24	1.37
orf19.3942.1	RPL43A	1.31	0.92	1.32
orf19.3945		1.22	1.28	1.12
orf19.3947		1.56	1.04	1.46
orf19.3949		1.53	1.47	1.07
orf19.395	ENO1	0.77	0.71	0.74
orf19.3950	MSM1	0.57	0.42	0.55
orf19.3951	TIP1	2.14	0.88	1.42
orf19.3952		1.83	1.38	1.18
orf19.3954		1.42	1.06	1.13
orf19.3955	MES1	1.25	0.85	0.86
orf19.3956		1.51	1.37	1.23
orf19.3957		1.35	1.23	1.15
orf19.3959	SSD1	1.03	0.87	1.01
orf19.396	EAF6	1.81	1.92	2.25
orf19.3960		1.92	1.26	1.51
orf19.3962	HAS1	1.00	1.00	0.72
orf19.3963		1.58	0.92	1.27
orf19.3964	ASH2	0.97	0.54	0.74

orf19.3965		1.60	2.68	1.06
orf19.3968		1.15	0.86	0.69
orf19.397		1.15	0.75	0.89
orf19.3977		1.26	1.02	1.00
orf19.3982		1.14	0.88	1.30
orf19.3983		1.49	0.98	0.89
orf19.3990		0.78	0.71	0.96
orf19.3991		1.26	1.19	1.48
orf19.400	GCF1	1.29	1.04	1.09
orf19.4000		1.48	0.85	0.91
orf19.4002	DUN1	1.56	1.33	1.14
orf19.4004	CCT3	1.64	1.11	1.10
orf19.401	TCP1	1.23	0.69	0.84
orf19.4013		1.20	1.05	1.51
orf19.4014		1.76	1.43	1.33
orf19.4018		1.12	0.68	0.96
orf19.4022	SDH4	1.06	0.71	0.97
orf19.4025	PRE1	1.87	1.56	1.60
orf19.4026	HIS1	1.09	1.12	1.04
orf19.4029		1.05	1.46	0.77
orf19.4032	RPN5	2.39	2.21	1.91
orf19.4035	PGA4	1.59	0.25	1.26
orf19.4036	APM1	1.85	1.09	1.93
orf19.4040	ILV3	1.54	1.14	1.27
orf19.4043		1.40	0.93	1.39
orf19.4044	MUM2	2.00	1.08	1.67
orf19.4045	EST1	1.27	0.99	1.29
orf19.4048	DES1	1.10	1.23	1.43
orf19.405	VCX1	2.28	0.42	1.59
orf19.4051	HTS1	1.23	0.73	1.10
orf19.406	ERG1	0.55	0.85	0.81
orf19.4060	ARO4	1.10	1.05	0.87
orf19.407	GCD6	1.68	1.24	1.05
orf19.4072	IFF6	2.30	1.05	1.63
orf19.4078		1.30	1.45	1.27
orf19.4079	SLP2	1.25	1.12	1.14
orf19.408		1.15	0.80	0.85
orf19.4084	KIS1	1.73	1.50	1.37
orf19.4085		2.35	2.00	1.75
orf19.4089	SGT1	1.53	0.97	1.24
orf19.409		0.94	1.37	0.77
orf19.4090.1		1.45	0.88	1.55
orf19.4093	NOP7	1.06	0.93	0.73
orf19.4099	ECM17	1.66	0.80	0.84
orf19.410		1.91	2.33	1.39
orf19.4109	PMT4	1.56	0.86	1.32
orf19.4112		1.10	0.85	0.92
orf19.4117		1.46	1.08	1.42
orf19.412		1.29	0.86	1.33
orf19.4120	LAS1	0.74	0.73	0.62
orf19.4122		4.25	2.41	1.58
orf19.4125		0.76	0.84	0.75
orf19.413.1	RPS27A	2.33	1.57	1.46
orf19.4132		3.19	1.34	1.56
orf19.4138		1.61	2.09	1.29
orf19.414		0.69	0.62	0.47
orf19.4142		2.27	0.85	1.23
orf19.4143		1.31	0.96	1.03
orf19.4146	SMD3	1.80	1.04	1.16
orf19.4149.1		1.35	0.83	1.09
orf19.4154		1.39	1.10	0.97
orf19.4155.11		1.65	0.81	0.88
orf19.4159		1.69	1.14	1.29
orf19.4164		1.76	1.50	1.22
orf19.4166		1.16	0.94	0.94
orf19.417		1.14	0.87	1.07
orf19.4170		1.17	0.95	0.85

orf19.4172		1.85	1.37	4.53
orf19.4177	HIS5	1.68	1.04	1.47
orf19.4182		1.49	0.93	1.21
orf19.4186	PCT1	1.24	0.88	0.99
orf19.4188	NMD5	1.09	0.84	0.71
orf19.4190		1.12	0.88	0.98
orf19.4191	RLP24	0.84	0.87	0.64
orf19.4192	CDC14	1.50	0.31	0.91
orf19.4193		1.57	1.24	1.62
orf19.4193.1	RPS13	0.99	0.72	1.00
orf19.4194		1.79	1.24	2.13
orf19.4195.1	FCA1	1.33	1.26	1.21
orf19.4199		1.20	1.53	0.81
orf19.4203		1.44	0.83	1.73
orf19.4204		1.16	0.90	1.18
orf19.4205.1		1.49	1.12	1.50
orf19.4208	RAD52	1.62	1.34	1.33
orf19.4211	FET3	2.93	1.12	2.49
orf19.4219	RIX7	0.66	0.61	0.62
orf19.4221	ORC4	1.35	1.07	1.15
orf19.4223	GCD11	1.24	1.02	1.07
orf19.4225	LEU3	0.90	0.90	0.88
orf19.423		1.45	1.10	1.28
orf19.4233	THR4	1.15	1.16	0.99
orf19.4234		1.05	1.23	1.07
orf19.424	TRP99	1.49	1.03	1.48
orf19.4240		1.62	0.96	1.49
orf19.4241		0.90	0.47	0.88
orf19.4242	CST20	1.45	0.86	1.14
orf19.4247		2.51	1.13	2.26
orf19.4251		1.84	1.07	1.74
orf19.4252		1.47	0.96	1.00
orf19.4257	INT1	1.15	0.79	0.90
orf19.4258		1.35	0.97	1.34
orf19.4261	TIF5	1.07	0.81	1.00
orf19.4262		1.20	1.07	1.06
orf19.4265	UAP1	1.60	2.23	1.23
orf19.4268		0.67	1.04	0.67
orf19.4273		1.09	0.82	0.69
orf19.4281		0.76	0.73	0.87
orf19.4282		0.74	0.64	0.66
orf19.4283		1.35	0.94	1.24
orf19.4287		2.81	1.52	1.53
orf19.429		0.78	1.05	0.69
orf19.4293		1.15	1.24	1.29
orf19.4294		1.54	1.10	1.15
orf19.4297	CKB2	1.35	1.63	1.29
orf19.4299	MSW1	1.04	0.96	0.83
orf19.43		2.89	3.65	1.68
orf19.4305.1		2.03	2.17	1.70
orf19.4306		1.78	1.15	1.46
orf19.431		1.25	1.09	0.99
orf19.4311	YNK1	2.62	1.18	2.21
orf19.4318	MIG1	2.42	1.37	1.53
orf19.432		0.87	1.59	1.39
orf19.4322	DAP2	1.86	1.12	1.47
orf19.4326		1.79	1.23	1.07
orf19.433		1.89	1.58	1.57
orf19.4336	RPS5	1.16	0.95	1.18
orf19.4338		2.29	1.42	1.63
orf19.434	PRD1	1.82	1.59	2.01
orf19.4340		2.36	1.56	2.39
orf19.4346		1.97	1.62	1.77
orf19.4347		0.96	0.64	0.83
orf19.4349		2.02	1.05	1.54
orf19.4349.6		0.83	0.70	0.80
orf19.4351	PRP12	2.43	1.15	1.38

orf19.4354		2.62	0.77	1.30
orf19.4357		3.34	1.56	1.37
orf19.4358		1.85	1.32	1.57
orf19.4362		1.94	1.91	1.98
orf19.4368		7.30	1.72	1.68
orf19.437	GRS1	1.31	1.15	1.07
orf19.4373		2.25	1.98	1.77
orf19.4375		1.09	1.31	1.06
orf19.4375.1	RPS30	1.11	0.87	1.15
orf19.4381	VTC3	1.50	0.93	1.79
orf19.4382		1.55	1.35	1.84
orf19.439		1.37	1.36	1.18
orf19.4390		1.85	1.14	1.64
orf19.4399		0.69	1.27	0.76
orf19.440	SDH1	1.66	1.18	1.25
orf19.4413	CMD1	1.17	0.82	1.21
orf19.4427	SKP1	1.45	1.06	1.41
orf19.443		0.90	0.52	0.70
orf19.4432	KSP1	1.35	1.33	1.01
orf19.4435		1.21	0.57	0.93
orf19.4437		1.50	1.16	1.22
orf19.4440	SEC34	1.56	1.16	1.24
orf19.4442	ALG9	2.01	1.21	5.68
orf19.4443	YPD1	1.62	3.11	1.17
orf19.4444	PHO15	1.20	2.49	1.30
orf19.4446		2.13	1.58	1.54
orf19.445		1.27	0.77	1.04
orf19.4451	RIA1	1.05	0.85	0.62
orf19.4456	GAP4	1.72	0.59	0.96
orf19.4457	BNI4	1.70	1.48	1.60
orf19.4477	CSH1	1.77	0.94	1.24
orf19.4478		1.53	0.95	1.56
orf19.4479		0.70	1.05	0.67
orf19.4482	IFI3	1.65	1.26	1.40
orf19.4484		5.62	1.00	1.62
orf19.4490	RPL17B	1.24	0.92	1.11
orf19.4490.2	QCR8	1.60	1.00	1.44
orf19.4491	ERG20	1.24	0.69	0.88
orf19.4492		0.69	1.37	0.61
orf19.4494	KTR2	1.36	0.90	1.19
orf19.4495	NDH51	2.27	0.95	1.27
orf19.4496		1.43	0.94	1.34
orf19.4499	RIM2	0.46	0.79	0.50
orf19.4517		1.41	1.26	0.97
orf19.4519	SUV3	0.56	0.67	0.51
orf19.4521		1.23	0.87	0.97
orf19.4525		1.51	1.29	1.81
orf19.4532		1.09	0.96	0.84
orf19.4536	CYS4	1.20	0.74	0.79
orf19.4537		1.30	0.86	1.34
orf19.4538		1.63	1.34	1.09
orf19.454	SFL1	0.59	1.32	0.61
orf19.4542		1.60	1.59	1.12
orf19.4545	SWI4	1.51	1.12	1.34
orf19.4546	HOL4	0.97	1.18	0.82
orf19.4550		1.89	1.54	1.87
orf19.4552		1.42	2.28	1.30
orf19.4558		1.60	0.56	0.89
orf19.4560		1.16	0.93	1.10
orf19.4563		0.50	0.78	0.36
orf19.4565	BGL2	1.38	0.84	1.18
orf19.4577.3		1.44	1.07	1.44
orf19.458		1.31	0.98	0.93
orf19.4582		1.17	1.34	0.85
orf19.4587	HGH1	0.97	1.17	1.09
orf19.4605	TYR1	1.89	1.41	1.21
orf19.4606	ERG8	2.29	1.10	1.34

orf19.461		2.93	1.71	2.41
orf19.4611		1.02	1.07	0.85
orf19.4615		2.08	1.02	1.47
orf19.4617		0.64	2.03	1.21
orf19.462		3.46	1.38	2.15
orf19.4620		0.98	1.50	1.17
orf19.4621		1.05	1.72	1.37
orf19.4622		1.41	1.12	1.08
orf19.4623		1.33	0.96	0.74
orf19.4623.3	NHP6A	1.14	0.62	1.18
orf19.4625	TOA2	1.50	1.15	1.03
orf19.4627		1.50	1.79	1.03
orf19.4632	RPL20B	1.34	0.82	1.05
orf19.4633		1.72	1.27	1.20
orf19.4634		1.04	0.99	0.48
orf19.4635	NIP1	1.16	1.22	1.03
orf19.4639		0.94	0.72	0.82
orf19.4640	PWP1	0.92	0.89	0.80
orf19.4641	NMT1	1.62	0.99	1.17
orf19.4657		0.61	0.51	0.35
orf19.4658		1.45	1.51	1.90
orf19.4659		1.11	0.93	1.02
orf19.4660	RPS6A	1.09	0.75	0.98
orf19.4664		1.05	1.29	1.56
orf19.4665		1.42	1.06	1.35
orf19.4666		1.33	0.88	0.74
orf19.4676		2.24	0.79	1.54
orf19.4678		1.87	1.48	1.27
orf19.4679	AGP2	1.84	1.01	1.00
orf19.4681	RAT1	0.69	1.14	0.69
orf19.4683	MLP1	1.67	0.83	0.89
orf19.4684.2	RPL40B	1.13	0.70	1.12
orf19.4686		1.34	1.99	1.27
orf19.4689	PGA57	1.86	1.20	1.24
orf19.469	HST7	2.11	1.17	1.86
orf19.4697	MDN1	1.51	1.36	0.87
orf19.4704	ARO1	1.61	1.27	1.26
orf19.4705		1.09	0.80	0.78
orf19.4718	TRP5	1.49	1.74	0.93
orf19.4719	CWH41	1.90	1.14	1.38
orf19.4721		1.38	1.17	1.38
orf19.4726		2.82	2.57	2.84
orf19.4732	SEC24	2.46	1.28	1.76
orf19.4746	JIP5	0.51	0.75	0.37
orf19.4747	HEM14	1.17	3.21	1.28
orf19.4751		1.26	1.41	1.26
orf19.4753	PFK26	1.64	2.06	1.85
orf19.476		1.78	1.00	2.04
orf19.4760		0.77	0.99	0.54
orf19.4766		1.85	1.57	1.84
orf19.477		1.63	1.11	1.47
orf19.4771		2.91	1.77	1.40
orf19.4777	DAK2	1.09	2.59	1.62
orf19.4796		1.90	1.22	1.58
orf19.4808	NUP188	0.83	1.10	0.88
orf19.4809	ERG12	1.55	0.63	1.38
orf19.481	GCD1	1.43	0.97	1.10
orf19.4813	GUA1	0.93	0.45	0.73
orf19.4815	YTM1	0.80	0.88	1.07
orf19.4816		1.20	1.35	1.00
orf19.4819		1.62	1.16	1.64
orf19.482	RPT4	1.81	1.90	1.50
orf19.4820		1.09	1.74	1.10
orf19.4824		1.37	1.31	1.26
orf19.4826	IDH1	1.56	1.21	1.16
orf19.4827	ADE12	1.04	0.91	0.97
orf19.4830		2.34	1.08	2.09

orf19.4835		0.45	0.82	0.39
orf19.4836	URA1	0.77	0.99	0.93
orf19.4837		1.71	1.90	1.28
orf19.4839		1.78	0.86	1.65
orf19.484	MRPL40	0.98	0.52	0.76
orf19.4844		1.21	0.75	0.64
orf19.4845		1.39	0.96	1.52
orf19.4846		0.78	0.94	0.87
orf19.4848	SKI3	1.73	1.07	1.12
orf19.4859		9.01	2.99	2.22
orf19.4862		2.09	0.87	1.99
orf19.4862.2		1.78	2.51	1.72
orf19.4865		1.21	0.67	1.08
orf19.4870	DBP3	0.77	0.78	0.83
orf19.4874	MNN3	1.26	1.18	1.16
orf19.4879.2	NTF2	0.82	0.55	0.81
orf19.488	MEX67	1.51	1.42	1.11
orf19.4882		1.42	0.56	0.89
orf19.4885	MIR1	2.03	0.95	1.56
orf19.4888		1.69	2.09	3.41
orf19.4889		0.73	0.55	0.81
orf19.489	DAP1	0.71	2.51	1.24
orf19.4896		1.10	1.21	0.98
orf19.4897		1.86	1.31	1.45
orf19.4898		1.66	3.03	1.44
orf19.490		1.30	1.71	1.49
orf19.4903		2.23	1.11	2.30
orf19.4909	CBK1	1.93	1.07	1.12
orf19.4909.1	RPL42	1.05	0.65	0.91
orf19.4921		2.73	2.09	2.58
orf19.4922		1.41	1.05	1.68
orf19.4927	BNI1	1.56	1.05	1.33
orf19.4929		0.87	0.99	0.77
orf19.493	RPL15A	1.18	0.97	1.14
orf19.4931.1	RPL14	1.23	0.84	1.06
orf19.4932		1.27	0.70	0.97
orf19.4937	CHS3	1.52	1.19	1.22
orf19.4938		2.20	1.53	1.61
orf19.4939		1.98	1.09	2.08
orf19.4940		1.17	0.96	0.68
orf19.4941	TYE7	1.23	0.42	1.37
orf19.4947		1.50	0.99	1.37
orf19.4951		1.74	2.12	1.18
orf19.4952.1		1.59	2.07	1.39
orf19.4955		1.90	0.93	1.69
orf19.4959		1.37	0.84	1.05
orf19.4960		1.23	1.47	0.90
orf19.4963		0.76	0.62	0.58
orf19.4966		2.59	1.78	1.27
orf19.497	EAF7	1.49	1.18	1.07
orf19.498		1.08	0.67	0.78
orf19.4980	HSP70	4.18	1.13	1.50
orf19.4987	NUP49	1.64	1.52	1.42
orf19.4998		1.08	0.64	1.07
orf19.5		1.88	0.81	1.19
orf19.500		0.78	0.79	0.69
orf19.5006		1.24	0.71	1.10
orf19.5007	ACT1	1.29	0.83	1.14
orf19.5009		0.91	1.19	0.85
orf19.501		0.80	0.87	0.53
orf19.5010	DIM1	0.54	0.73	0.71
orf19.5015	MYO2	1.96	0.90	1.33
orf19.5016		1.35	0.93	1.05
orf19.5020		0.58	0.29	0.59
orf19.5021	PDX1	1.16	0.97	0.83
orf19.5023	DAL7	1.17	1.52	1.12
orf19.5024	GND1	1.16	1.50	1.03

orf19.5026		2.15	1.44	1.07
orf19.5027	LCB2	2.13	0.98	2.19
orf19.5030		2.29	1.63	2.47
orf19.5034		1.12	0.94	1.30
orf19.5036		1.99	1.17	1.92
orf19.5038		0.52	0.53	0.41
orf19.5040		1.33	1.49	1.10
orf19.505	SRV2	1.71	1.34	1.49
orf19.5054		1.58	1.02	1.36
orf19.5058	SMI1	2.17	1.64	1.44
orf19.5059	GCS1	0.69	2.05	0.61
orf19.506	YDJ1	1.29	1.05	0.93
orf19.5061	ADE5,7	1.15	0.80	0.82
orf19.5062		1.35	0.69	0.80
orf19.5063		0.92	0.50	0.81
orf19.5064	MRPL3	1.01	0.64	0.77
orf19.5065		1.81	1.25	1.44
orf19.5066		0.92	0.91	0.58
orf19.5068		1.14	1.46	1.51
orf19.5071	NRP1	1.07	0.81	0.90
orf19.5076	PFY1	1.40	1.11	1.38
orf19.5079	CDR4	1.94	5.39	1.57
orf19.5081	FUN12	1.11	0.75	0.77
orf19.5083	DRG1	1.25	1.02	1.01
orf19.5085		1.13	0.84	0.95
orf19.5093		2.10	1.07	1.19
orf19.51		1.52	1.52	1.04
orf19.5100	MLT1	1.15	0.71	0.97
orf19.5103		1.56	1.42	2.37
orf19.5104	LTP1	1.32	0.93	1.13
orf19.5105		0.78	0.88	0.84
orf19.5106	DIP2	0.66	1.13	0.64
orf19.5107	NOT5	1.66	1.85	1.05
orf19.5117	OLE1	0.69	0.31	0.62
orf19.512		0.93	0.68	0.55
orf19.5126		1.07	1.22	1.05
orf19.5128		1.20	1.23	1.00
orf19.5131		3.86	3.34	2.91
orf19.5133		1.22	2.29	1.84
orf19.5136		0.52	1.92	0.93
orf19.5137.1	HHO1	1.23	0.30	1.19
orf19.514	SNP3	1.09	0.53	0.96
orf19.5143		1.25	0.79	0.89
orf19.5148	CYR1	2.44	0.76	1.84
orf19.5155	CHS6	2.67	1.18	1.52
orf19.5158		2.10	2.19	2.01
orf19.516		2.14	0.97	1.15
orf19.5160		1.27	0.94	1.07
orf19.5161		1.20	0.82	0.96
orf19.5164	ECM39	1.48	0.74	1.31
orf19.5165		2.36	1.26	1.71
orf19.5168		1.44	0.91	0.95
orf19.5170	ENA21	0.66	0.62	0.63
orf19.5171	PMT1	1.42	0.61	1.19
orf19.5178	ERG5	1.01	0.41	0.81
orf19.518		0.90	0.76	0.68
orf19.5180		1.37	1.00	1.08
orf19.5184		2.18	1.30	1.48
orf19.519		0.97	0.73	0.87
orf19.5192		2.06	0.82	0.88
orf19.5195		1.12	0.98	1.16
orf19.5196	ESS1	1.69	1.93	1.39
orf19.5197	APE2	1.13	0.87	0.87
orf19.5198	NOP4	0.59	1.23	0.43
orf19.5200	SIT4	2.17	1.85	1.67
orf19.5201		1.31	0.90	1.17
orf19.5203		2.23	1.47	1.62

orf19.5206		0.61	0.99	0.54
orf19.5208		0.62	0.93	0.70
orf19.5211	IDP1	1.87	1.26	1.41
orf19.5213.1	COX8	1.33	0.88	1.27
orf19.5213.2	COX9	1.36	0.77	1.20
orf19.5216		1.14	0.93	1.38
orf19.5219	IRA2	1.11	1.61	0.92
orf19.5226	WRS1	1.66	0.97	1.38
orf19.5228	RIB3	0.88	1.85	0.93
orf19.5229		1.03	0.89	0.83
orf19.5230	MRPS9	1.17	1.23	1.08
orf19.5232	CSI2	0.74	0.83	0.64
orf19.5234	RBD1	1.52	1.12	1.25
orf19.5235		1.17	0.65	0.87
orf19.5241	SNT1	1.10	0.95	0.78
orf19.5242	CDC6	0.98	0.28	0.75
orf19.5243	TRP3	1.95	1.94	1.23
orf19.5244	MCD4	2.19	1.08	1.67
orf19.526	NHP2	1.17	0.97	0.88
orf19.5263	SER33	1.13	1.38	1.00
orf19.5267		2.05	0.76	1.48
orf19.5278		1.11	0.71	1.01
orf19.5279		1.09	0.87	1.03
orf19.528		1.41	1.13	1.33
orf19.5281		1.34	0.73	0.90
orf19.5288	IFE2	0.75	4.70	1.19
orf19.5293		1.64	1.21	1.35
orf19.5294	PDB1	1.06	0.69	0.75
orf19.5295		2.68	1.23	1.60
orf19.5299	ECM1	0.76	0.73	0.67
orf19.5321		1.73	1.19	1.40
orf19.5323	MDH1-3	1.98	1.17	1.85
orf19.5328	GCN1	1.08	0.54	0.66
orf19.5329		1.20	0.76	0.93
orf19.5334		0.79	0.64	0.86
orf19.5337	UBC15	1.87	1.10	1.63
orf19.5340		2.51	1.11	1.46
orf19.5341	RPS4A	1.14	0.52	0.92
orf19.5342.1	MTR2	1.04	0.98	1.19
orf19.5343	ASH1	0.94	0.38	0.88
orf19.5348	TPS3	1.11	1.28	1.15
orf19.5350		1.36	1.21	1.42
orf19.5351	TIF11	1.32	0.92	1.10
orf19.5356		0.61	1.37	0.48
orf19.536		1.93	1.17	1.37
orf19.5360	RPC11	1.27	1.01	1.12
orf19.5364		1.42	1.08	1.31
orf19.5365		1.20	0.88	0.88
orf19.5366		1.53	1.05	1.16
orf19.5369		1.51	0.90	1.51
orf19.5375		1.73	0.86	1.56
orf19.5377	HOS2	1.93	0.94	1.70
orf19.5383	PMA1	1.24	0.81	0.85
orf19.5384	CHS8	1.20	0.58	0.96
orf19.5390		1.31	2.03	1.39
orf19.5391		0.81	1.08	0.80
orf19.5392	NGT1	2.17	1.22	1.98
orf19.5393		1.01	1.45	0.93
orf19.5394.1		1.38	0.93	0.89
orf19.54	RHD1	1.13	0.74	1.82
orf19.5406		1.43	1.08	1.15
orf19.5407		0.63	0.76	0.51
orf19.5408		1.00	2.90	1.20
orf19.5411		2.14	1.27	2.01
orf19.5418		1.31	0.67	1.30
orf19.542	HXK2	1.11	1.54	1.17
orf19.542.2		1.49	1.50	1.69

orf19.5420		1.05	0.78	0.80
orf19.5425		0.72	0.81	0.67
orf19.543		2.35	1.40	1.62
orf19.5430	BUD21	0.93	1.76	0.96
orf19.5432	TPT1	1.22	0.99	1.16
orf19.5433		0.98	0.57	1.06
orf19.5436		1.20	0.87	0.84
orf19.5437	RHR2	0.77	1.17	0.63
orf19.544.1	PRE6	1.69	1.43	1.60
orf19.5444		1.19	0.92	1.19
orf19.5454	DAL1	3.08	1.22	2.31
orf19.5455		1.42	0.66	2.02
orf19.5459		1.56	0.68	1.20
orf19.5463		1.86	1.19	1.26
orf19.5466	RPS24	1.40	0.84	1.34
orf19.5475		2.06	1.77	1.21
orf19.5477		1.66	0.58	0.81
orf19.5480	ILV1	1.33	1.04	1.73
orf19.5483		1.69	1.03	1.60
orf19.5486		1.35	1.42	1.07
orf19.5486.1	SMD2	0.91	0.55	0.98
orf19.5487	CDC46	1.65	0.61	1.10
orf19.549		0.84	0.52	0.81
orf19.5493	GSP1	1.41	0.99	1.22
orf19.5495		1.39	0.77	1.19
orf19.55		0.65	1.00	1.10
orf19.550	PDX3	1.11	1.42	0.97
orf19.5500	MAK16	0.63	0.90	0.66
orf19.5501	YAF9	2.77	1.83	1.60
orf19.5505	HIS7	1.65	1.59	1.27
orf19.5510		1.03	0.83	1.01
orf19.5515		1.13	0.83	1.14
orf19.5516		1.45	1.10	1.12
orf19.5519	GCV1	1.99	0.84	1.36
orf19.552		3.42	1.34	2.62
orf19.5525		2.14	2.41	1.69
orf19.5530	NAB3	1.66	1.82	1.11
orf19.5534		1.21	1.43	1.33
orf19.5535		1.41	0.83	1.46
orf19.5541		0.81	0.35	0.45
orf19.5547		1.85	1.33	1.60
orf19.5550	MRT4	0.78	1.12	0.72
orf19.5553		1.30	1.44	1.13
orf19.5558	RBF1	1.58	0.69	1.02
orf19.5563	RNH1	1.62	0.99	1.80
orf19.5564		1.64	0.99	1.54
orf19.5569		1.27	1.74	1.28
orf19.5571		1.02	0.86	1.00
orf19.5572		1.02	0.77	0.77
orf19.5574		1.52	0.97	1.58
orf19.5576		0.61	0.32	0.67
orf19.558	GUT1	0.78	0.38	0.53
orf19.559	FGR14	0.80	1.16	0.94
orf19.5591	ADO1	1.30	1.01	1.09
orf19.5597	POL5	1.00	1.10	0.82
orf19.5599	MDL2	1.69	1.01	0.99
orf19.5600		1.27	1.04	0.97
orf19.5607		1.05	0.82	0.71
orf19.5608		0.82	0.85	0.76
orf19.5614		2.65	1.01	1.45
orf19.5615	AYR2	1.69	1.14	1.50
orf19.5618		2.24	3.86	2.10
orf19.5619		2.03	1.05	1.90
orf19.5622	GLC3	2.05	1.52	1.62
orf19.5623	ARP4	1.68	0.88	1.20
orf19.5624		1.48	0.67	1.28
orf19.5627		1.33	1.15	0.97

orf19.5628		0.87	0.96	0.80
orf19.563	RRP15	0.61	1.36	0.46
orf19.5630	APA2	1.21	1.82	1.34
orf19.5631		2.24	1.29	1.78
orf19.5639	HIS4	1.45	1.26	1.16
orf19.5642		1.53	0.62	1.50
orf19.5645	MET15	2.26	0.66	1.08
orf19.5647	SUB2	1.39	1.04	1.30
orf19.5650	PRO3	2.34	1.22	1.99
orf19.5657	SWI1	1.28	1.00	1.26
orf19.5658	MNN10	1.33	0.91	1.33
orf19.5661	PTC7	1.29	0.96	1.13
orf19.5665		1.51	0.99	1.46
orf19.5667	MNR2	1.51	1.97	1.27
orf19.5669		1.65	1.15	1.37
orf19.5675		0.92	1.17	0.95
orf19.5678		0.63	0.89	0.44
orf19.5679		1.40	1.10	0.93
orf19.568	SPE2	0.89	1.11	0.69
orf19.5680		1.87	1.74	2.26
orf19.5682		1.03	0.62	0.91
orf19.5684		0.97	0.86	1.19
orf19.5685	THS1	1.29	0.75	1.06
orf19.5686		1.34	1.10	1.13
orf19.5691	CDC11	2.14	1.18	1.27
orf19.5693		1.67	0.70	1.21
orf19.5694		1.75	1.55	2.04
orf19.5698		1.35	0.76	1.09
orf19.5702		1.17	1.40	1.10
orf19.5704		0.54	0.81	0.53
orf19.5705	NAM2	1.79	1.05	1.50
orf19.5710		1.69	0.98	1.34
orf19.5720		2.53	2.25	1.37
orf19.5722		1.34	0.59	1.19
orf19.5732	NOG2	0.56	1.03	0.30
orf19.5734		2.27	1.79	1.10
orf19.5742	ALS9	1.64	2.41	1.35
orf19.5746	ALA1	1.70	1.14	1.16
orf19.5747		1.18	1.02	0.85
orf19.5750	SHM2	1.11	0.52	0.79
orf19.5751	ORM1	1.72	1.09	1.07
orf19.5759	SNQ2	1.17	1.70	0.66
orf19.5764	SKI8	1.45	1.22	0.96
orf19.5765		1.71	1.66	1.45
orf19.5767		1.97	1.25	1.88
orf19.5771	PBP2	1.37	1.49	1.39
orf19.5773		2.79	0.94	1.81
orf19.5779	RNR1	1.06	1.06	0.90
orf19.5783		0.87	0.71	0.68
orf19.5788	EFT2	1.12	0.62	0.88
orf19.5789	ADE8	1.27	0.78	0.99
orf19.5791	IDH2	1.96	1.50	1.60
orf19.5799		1.24	0.90	0.91
orf19.58	RRP6	0.52	0.74	0.38
orf19.580		2.10	3.23	2.73
orf19.5801	RNR21	1.21	0.50	1.28
orf19.581		1.08	1.96	0.89
orf19.5812		0.79	1.17	0.80
orf19.5813		1.68	2.63	1.17
orf19.5817		1.74	1.96	1.30
orf19.5818	SUR2	0.46	2.05	0.89
orf19.5825		1.78	1.49	1.18
orf19.5825.1		2.42	0.81	1.49
orf19.5828		1.63	0.94	1.18
orf19.583		1.14	1.25	0.87
orf19.5832	HPT1	0.95	0.72	0.80
orf19.5833		1.45	1.56	1.31

orf19.5834		1.09	1.02	1.04
orf19.5838		1.29	1.00	1.09
orf19.5839	PDR17	1.41	0.76	1.59
orf19.5847		1.05	1.34	0.64
orf19.585		0.79	0.73	0.75
orf19.5850	NOC2	0.83	1.10	0.64
orf19.5854	SBP1	1.28	1.04	1.09
orf19.5858	EGD2	1.39	1.00	1.32
orf19.586	ERV46	1.61	1.08	1.58
orf19.5861	KRE9	1.47	0.94	1.43
orf19.5864	URK1	0.94	1.65	1.17
orf19.5870	CTP1	1.52	2.32	1.80
orf19.5872		1.43	0.56	1.69
orf19.5873	POL1	2.69	1.35	1.72
orf19.5881		3.60	1.39	1.61
orf19.5886	CUP5	2.07	0.49	1.77
orf19.5887	NUP85	1.65	0.92	1.01
orf19.5892		1.75	2.44	2.55
orf19.5893	RIP1	1.64	0.82	1.26
orf19.5897		2.33	1.35	2.39
orf19.5903	RAX1	0.84	0.71	0.87
orf19.5904	RPL19A	1.35	0.98	1.18
orf19.5905		1.43	0.41	0.90
orf19.5906	ADE2	1.28	0.74	0.67
orf19.5911	CMK1	1.30	1.50	1.21
orf19.5917.3		1.72	0.99	1.52
orf19.5927	RPS15	1.14	0.67	1.06
orf19.5928	RPP2B	0.95	0.53	0.81
orf19.5938	SEN1	2.91	1.49	1.86
orf19.5943.1		1.14	0.83	1.16
orf19.5947	SEC7	1.41	0.82	1.05
orf19.5958	CDR2	1.58	0.77	1.46
orf19.5959	NOP14	0.69	0.75	0.47
orf19.596.1	NOP10	0.78	0.49	0.78
orf19.5964	ARF2	1.59	1.06	1.43
orf19.5964.2	RPL35	1.17	0.79	1.03
orf19.5967	FGR44	1.30	0.66	0.86
orf19.597		1.37	1.62	1.17
orf19.5973		3.66	1.46	1.31
orf19.5974	ATG9	1.41	1.11	1.34
orf19.5982	RPL18	1.21	0.70	1.19
orf19.5985		1.64	1.11	1.26
orf19.5987		0.91	1.52	0.89
orf19.5991		0.47	0.59	0.38
orf19.5995	MCA1	1.83	0.88	1.17
orf19.5996.1	RPS19A	1.41	0.75	1.19
orf19.600	TRK1	0.97	0.59	1.17
orf19.6000	CDR1	1.43	0.92	0.86
orf19.6005	HGT5	3.57	1.04	1.70
orf19.6010.1	RPB11	1.13	0.80	0.80
orf19.6011	SIN3	1.60	1.33	1.23
orf19.6014	RRS1	0.49	0.74	0.48
orf19.6017		1.61	1.22	1.13
orf19.6018	LRO1	1.54	0.66	1.19
orf19.6019		2.13	0.72	1.64
orf19.602		1.02	0.68	0.87
orf19.6027		1.57	1.18	1.41
orf19.6029	ROT1	0.87	0.55	0.95
orf19.603	IMP4	0.66	1.02	0.55
orf19.6031		1.40	1.03	1.12
orf19.6032	SPE1	1.26	1.04	1.00
orf19.6033	CMP1	1.77	1.52	1.24
orf19.6035		1.42	0.73	1.19
orf19.6041	RPO41	0.75	0.84	0.65
orf19.6047	TUF1	1.14	0.57	0.97
orf19.6049		2.95	0.77	0.98
orf19.6052	CNS1	1.03	0.87	0.82

orf19.6056		1.31	1.85	2.08
orf19.6060	GCN20	1.71	1.10	1.31
orf19.6062.3		2.03	1.10	1.57
orf19.6065		1.92	1.82	1.95
orf19.6068	SVF1	2.07	1.16	1.12
orf19.6074	HBR1	1.01	1.08	0.87
orf19.6075		1.69	1.00	1.13
orf19.6081	PHR2	1.00	1.06	1.00
orf19.6085	RPL16A	1.36	0.87	1.19
orf19.6090		1.02	0.94	0.82
orf19.6096	TRP1	1.59	1.19	1.18
orf19.6099	CCT8	1.57	0.81	1.19
orf19.6100		1.69	0.86	1.27
orf19.6105	MVD	1.17	0.69	1.04
orf19.6114		3.11	2.37	1.56
orf19.6116	GLK4	1.01	1.75	1.01
orf19.6118		1.35	0.83	1.05
orf19.6127	LPD1	1.57	1.30	1.22
orf19.6129	MRPL8	1.30	0.82	0.99
orf19.6133	PIF1	1.10	1.25	0.97
orf19.6134		1.60	0.92	1.28
orf19.6136		1.34	1.09	1.04
orf19.6146	CLG1	1.15	0.81	0.80
orf19.6147		1.38	0.95	1.04
orf19.6154		1.07	0.82	0.95
orf19.6156		1.47	0.95	1.41
orf19.6160		1.69	0.78	0.77
orf19.6171		1.87	1.11	1.22
orf19.6173	STD1	1.46	0.49	0.96
orf19.6175		1.18	1.07	0.80
orf19.6177		1.32	0.95	1.42
orf19.6178	FBP1	2.87	1.46	1.72
orf19.6183		1.07	0.55	0.97
orf19.6185		1.48	1.17	1.09
orf19.6186		1.97	1.11	1.32
orf19.6187		1.01	1.02	0.84
orf19.6189		1.61	1.04	1.50
orf19.6190	SRB1	1.28	0.92	1.26
orf19.6195		0.90	1.03	0.95
orf19.6198.1		1.26	0.86	1.10
orf19.62		1.46	0.66	0.80
orf19.6209		1.15	0.61	0.81
orf19.6213	SUI2	1.33	1.10	1.13
orf19.6217	PGA63	1.43	0.74	1.15
orf19.6219		2.03	1.48	2.64
orf19.6220.3	MMD1	1.48	0.51	1.25
orf19.6220.4		1.11	0.67	0.93
orf19.6227		1.04	0.79	1.25
orf19.6231	MRPL19	1.52	0.93	1.15
orf19.6233		1.26	0.93	1.25
orf19.6236		0.94	1.09	0.88
orf19.6237	RAC1	1.89	0.65	1.61
orf19.6239		3.40	2.42	1.76
orf19.6240		1.10	0.70	1.01
orf19.6241		1.60	0.72	1.09
orf19.6242		1.45	0.35	1.04
orf19.6246		1.20	1.82	1.70
orf19.6253	RPS23A	1.04	0.61	1.04
orf19.6257	GLT1	1.82	0.91	1.36
orf19.6259		1.07	0.76	0.95
orf19.6261	BPH1	2.04	1.64	1.42
orf19.6264		1.91	0.59	1.27
orf19.6264.4		1.27	0.71	1.09
orf19.6265	RPS22A	1.08	0.75	1.03
orf19.6265.1	RPS14B	1.15	0.75	1.00
orf19.6269		1.26	0.91	1.37
orf19.6271		1.32	1.36	1.02

orf19.6275		2.22	1.00	1.65
orf19.6283		1.29	1.02	1.37
orf19.6284		1.71	1.10	1.35
orf19.6285	GLC7	1.53	1.23	1.16
orf19.6286.2	RPS27	1.04	0.64	0.99
orf19.6294	MYO1	1.47	0.88	1.03
orf19.6295		1.63	0.91	1.01
orf19.6297		0.76	0.66	0.86
orf19.6299		1.79	1.97	1.69
orf19.6311		10.00	0.85	2.64
orf19.6312	RPS3	1.19	0.76	0.82
orf19.6314	RPB8	1.24	0.68	0.91
orf19.6316.4		1.01	0.97	0.90
orf19.6317	ADE6	1.01	0.87	0.78
orf19.6318		1.08	0.66	1.09
orf19.6319		1.38	1.57	1.14
orf19.6322	ARD	3.39	0.82	1.20
orf19.6327	HET1	2.01	0.93	1.77
orf19.6328		1.53	1.05	1.44
orf19.6340		1.01	0.95	0.76
orf19.6343		1.11	0.52	0.84
orf19.6344	RBK1	2.07	1.36	1.73
orf19.6345	RPG1A	1.26	1.10	0.93
orf19.6346		0.99	0.91	0.88
orf19.635.1		2.04	1.31	1.92
orf19.6355		0.81	1.11	0.69
orf19.6360		1.95	1.36	1.77
orf19.6362	SPA2	1.65	1.36	1.16
orf19.6367	SSB1	1.11	0.71	0.95
orf19.6369		0.89	0.85	0.79
orf19.6375	RPS20	1.21	0.67	0.99
orf19.6376	PTC5	2.90	0.89	1.42
orf19.6378	TRM9	1.15	0.87	0.98
orf19.6383		1.68	1.00	1.28
orf19.6387	HSP104	2.80	2.01	1.61
orf19.6389		3.02	1.76	1.83
orf19.6393		2.08	0.86	1.20
orf19.6396		1.42	1.95	1.51
orf19.64		2.39	1.24	1.41
orf19.6403		1.52	1.13	1.32
orf19.6403.1	RPP2A	0.92	0.52	0.80
orf19.6404	GSH2	1.72	1.22	1.59
orf19.6406		1.27	0.67	0.99
orf19.6414.3	TPM2	1.53	0.89	1.32
orf19.6417	TSR1	0.99	0.79	0.63
orf19.6418		0.91	0.85	0.56
orf19.642		1.86	0.68	1.02
orf19.6423		1.76	0.73	1.73
orf19.6424		1.34	0.73	1.21
orf19.6425		1.02	0.64	0.81
orf19.6432		0.90	0.89	1.11
orf19.6434		1.79	1.10	1.11
orf19.6436		3.68	1.24	1.89
orf19.6438		1.20	0.58	0.82
orf19.6442	PRP8	1.12	0.78	0.97
orf19.6448		1.61	0.92	1.23
orf19.6455		1.89	1.06	0.91
orf19.6458.1		1.10	0.47	0.84
orf19.6469		1.39	1.74	0.89
orf19.6474		3.50	1.36	1.90
orf19.6477		0.89	1.41	0.55
orf19.6481	YPS7	1.37	1.50	1.40
orf19.6498		2.43	1.07	1.60
orf19.6500	ECM42	1.35	1.13	2.58
orf19.6507		1.21	1.13	1.02
orf19.6512	EXO70	3.04	1.24	2.80
orf19.6515	HSP90	1.86	0.94	1.16

orf19.6524		1.29	0.82	1.09
orf19.6529	CDC34	0.89	2.28	0.92
orf19.6531	NUC2	2.09	1.15	1.72
orf19.6531.1	TOM71	1.08	0.60	1.03
orf19.6535		2.32	2.12	1.67
orf19.6539		1.27	1.09	0.86
orf19.6540	PFK2	0.65	1.54	0.91
orf19.6541	RPL5	1.00	0.94	0.89
orf19.6547		1.38	0.85	0.98
orf19.6557		2.06	1.24	1.73
orf19.6558		1.15	0.73	1.04
orf19.6563.1		1.18	0.81	1.23
orf19.6565		0.97	0.43	0.69
orf19.6568		1.30	0.82	1.33
orf19.6573	BEM2	2.05	1.40	2.03
orf19.6577	FLU1	0.73	0.98	0.72
orf19.6581		2.69	3.05	2.50
orf19.6582	PRE10	1.69	1.77	1.52
orf19.6584	PRT1	1.32	1.09	0.96
orf19.6587		1.19	0.74	0.89
orf19.6589		0.83	1.39	0.71
orf19.6594	PLB3	1.10	1.06	1.19
orf19.6599.1		1.30	0.82	1.12
orf19.6600		1.47	1.62	1.06
orf19.6604		1.38	0.97	1.06
orf19.661	KRR1	0.64	1.10	0.55
orf19.6612		1.06	0.64	1.18
orf19.6614	HPR5	13.61	1.72	1.38
orf19.6623		1.28	0.82	1.43
orf19.6625		1.10	0.66	0.96
orf19.6632	ACO2	1.77	1.12	1.02
orf19.6640	TPS1	1.06	2.16	1.23
orf19.6645	HMO1	1.39	0.72	1.31
orf19.6648		0.59	0.74	0.56
orf19.665	NEP1	0.83	0.45	0.56
orf19.6652	DBP8	0.85	0.89	0.65
orf19.6656	DUR3	1.44	0.94	1.24
orf19.6663	RPS25B	1.01	0.49	0.80
orf19.6665		1.24	0.97	1.15
orf19.6668		1.95	1.09	1.50
orf19.667.1	RPL37B	0.92	0.62	0.85
orf19.6676		1.36	0.80	1.29
orf19.668		3.23	2.69	1.92
orf19.6682		1.17	0.92	1.05
orf19.6686	ENP2	0.54	0.98	0.45
orf19.6691		0.57	0.87	0.53
orf19.6693		1.60	1.01	1.64
orf19.6696		1.71	1.36	2.33
orf19.6699		1.96	1.02	1.16
orf19.670	SMT3	1.00	0.78	0.94
orf19.6701		1.55	0.87	1.11
orf19.6702	DED81	1.59	1.08	1.22
orf19.6705		0.85	0.59	1.18
orf19.671	PSP1	2.72	1.02	1.41
orf19.6712		2.52	1.28	1.35
orf19.6713		1.74	0.94	1.28
orf19.6717		1.32	0.86	1.02
orf19.6718		1.01	1.34	0.92
orf19.672		2.39	1.05	2.11
orf19.6726		1.68	1.47	1.65
orf19.6729	TIP120	3.31	2.56	1.57
orf19.6730		0.77	1.05	0.80
orf19.6731.1		1.43	0.91	1.22
orf19.6734	TCC1	1.30	1.10	1.39
orf19.6738	VAN1	1.61	0.73	1.33
orf19.6739		1.89	1.44	1.64
orf19.674		1.05	1.39	0.59

orf19.6740		1.06	1.35	0.80
orf19.6748		1.11	0.79	1.09
orf19.6749	KRS1	1.01	0.75	0.82
orf19.675		0.65	1.28	0.67
orf19.6751		1.07	0.97	0.90
orf19.6752		1.25	0.86	0.95
orf19.6763	SLK19	2.57	1.62	1.61
orf19.6766		0.79	0.72	0.70
orf19.6769		1.32	1.26	1.33
orf19.677	CHO1	1.47	0.43	0.81
orf19.6771	UBI4	1.25	1.63	1.16
orf19.6773	ECM29	2.33	2.55	1.69
orf19.6776	GCD2	1.46	1.07	1.03
orf19.6779	PRO2	2.46	1.35	1.46
orf19.6780	MET8	1.42	0.97	1.21
orf19.6782	BMT1	1.56	0.91	1.96
orf19.6783		4.21	1.45	1.72
orf19.6785	RPS12	1.18	0.58	0.99
orf19.6794	FESUR1	1.91	1.03	1.59
orf19.6796		2.40	1.59	3.90
orf19.6797		1.58	1.31	0.89
orf19.680		1.23	0.82	0.97
orf19.6803	HUT1	1.53	1.21	1.41
orf19.6804		1.43	0.99	0.92
orf19.6805		2.17	1.66	1.81
orf19.6812	PMT2	1.59	0.71	1.36
orf19.6814	TDH3	0.84	0.71	0.81
orf19.6816		0.97	1.11	0.86
orf19.6818		1.31	1.31	0.99
orf19.682		2.64	1.15	1.39
orf19.6828.1		1.43	0.74	1.54
orf19.6829		1.16	1.04	0.95
orf19.6833		2.18	1.68	2.51
orf19.6834		2.01	0.60	1.15
orf19.6840		2.62	1.57	2.00
orf19.6845		3.74	0.88	1.41
orf19.6846	PHO85	1.45	1.11	1.35
orf19.6847		1.49	0.69	1.19
orf19.6849	ELC1	2.36	1.38	1.32
orf19.685	YHM1	1.19	0.41	0.85
orf19.6853		1.71	0.95	0.94
orf19.6854	ATP1	1.81	0.78	1.07
orf19.6856		1.80	1.18	0.88
orf19.6858		0.95	0.46	0.85
orf19.686		1.91	1.79	1.30
orf19.6860		1.55	1.13	1.17
orf19.6862		0.59	1.38	0.43
orf19.6865		0.97	0.89	0.72
orf19.6868		2.32	1.76	2.13
orf19.687.1	RPL25	1.27	0.91	1.27
orf19.6871		1.35	0.76	1.46
orf19.6872		2.17	1.31	1.31
orf19.6873	RPS8A	0.99	0.59	0.76
orf19.6875	VPS35	2.20	1.64	1.39
orf19.6879		2.01	1.03	1.26
orf19.688		1.44	0.90	1.21
orf19.6881	YTH1	1.15	1.23	0.90
orf19.6882	OSM1	0.66	1.28	0.90
orf19.6882.1		1.09	0.82	1.02
orf19.6885	SPO7	1.32	0.89	1.21
orf19.6886		0.66	0.80	0.56
orf19.6889	MKK2	1.93	2.20	1.60
orf19.6891	RFC1	1.77	1.04	2.33
orf19.6898.1		1.49	0.94	1.63
orf19.6903		0.87	1.22	1.01
orf19.6904	GCN3	1.07	0.98	1.00
orf19.6907		1.49	1.35	1.23

orf19.6910		0.75	0.76	0.82
orf19.6921		1.12	1.32	1.31
orf19.6923		2.52	1.32	1.39
orf19.693		1.72	1.07	1.65
orf19.6930	SOL1	1.76	0.83	0.99
orf19.6931		2.04	1.29	1.27
orf19.6937	PTR2	0.55	1.08	0.55
orf19.6938		1.99	1.21	1.63
orf19.6939		1.26	1.25	1.14
orf19.6951		1.66	1.14	2.14
orf19.6955	HBR3	0.80	1.00	0.62
orf19.696	STE2	1.96	1.07	1.53
orf19.6967	USO6	1.23	2.24	1.42
orf19.6972	SMI1B	1.57	1.15	1.01
orf19.6975	YST1	1.10	0.94	1.06
orf19.6976		2.40	1.02	1.14
orf19.6977	GPI1	1.10	1.24	1.10
orf19.6988		2.16	0.81	1.83
orf19.6992		0.76	2.54	0.81
orf19.6993	GAP2	0.72	1.68	1.00
orf19.6994	BAT22	1.85	1.33	1.48
orf19.6996		0.56	1.06	0.66
orf19.7011		0.66	0.93	0.66
orf19.7012		0.99	0.51	1.06
orf19.7015	RPP0	1.23	0.68	1.04
orf19.7017	YOX1	1.73	0.86	1.63
orf19.7018	RPS18	1.11	0.63	1.07
orf19.7019	YML6	1.28	0.93	1.09
orf19.702		1.00	1.54	1.08
orf19.7020		1.11	1.10	1.08
orf19.7021	GPH1	1.07	0.23	1.15
orf19.7023		2.71	1.89	1.52
orf19.7025	MCM1	1.90	0.50	1.22
orf19.7034		1.14	1.02	0.88
orf19.7036		1.48	1.22	1.14
orf19.704	SOL3	1.63	1.34	1.56
orf19.7041		1.18	1.07	0.82
orf19.7043.1	ACB1	1.18	0.73	1.12
orf19.7044		2.11	1.19	1.60
orf19.7046	MET28	1.36	1.39	1.07
orf19.7047	RTF1	1.87	1.05	1.72
orf19.7048.1	RPS28B	1.04	0.73	1.07
orf19.7049	CYB5	1.36	0.26	1.35
orf19.705	GCN5	1.31	0.89	0.99
orf19.7051		0.90	0.85	0.88
orf19.7052		5.80	1.38	1.93
orf19.7053	GAC1	1.02	0.45	0.78
orf19.7057		0.98	0.77	0.84
orf19.7062	RPA135	0.87	0.66	0.64
orf19.7063		0.93	0.53	0.84
orf19.7064	GLN4	1.13	1.18	0.76
orf19.7067		2.66	1.41	1.28
orf19.7068	MAC1	1.61	1.65	2.28
orf19.7069		0.67	0.49	0.53
orf19.7072	PEL1	0.95	1.02	0.85
orf19.7076		1.19	1.00	1.04
orf19.7077		2.53	1.02	1.86
orf19.7080	LEU2	2.24	2.22	2.70
orf19.7081	SPL1	1.19	1.96	0.84
orf19.7082		1.68	1.71	1.73
orf19.7088		0.98	1.07	1.36
orf19.7089	PMR1	1.10	0.62	1.06
orf19.7097		1.66	1.08	1.22
orf19.710		2.04	0.95	1.67
orf19.7101		1.38	0.90	1.32
orf19.7104		0.99	0.79	1.02
orf19.7107		0.97	0.83	0.81

orf19.7108		1.33	0.72	1.24
orf19.7121		2.22	0.94	1.45
orf19.7123		1.99	1.84	1.53
orf19.7125		1.82	1.24	1.12
orf19.7128	SYS1	2.19	1.01	1.45
orf19.713		1.82	1.58	2.45
orf19.7136	SPT6	1.34	0.84	0.87
orf19.714		2.13	1.13	1.34
orf19.7145		2.08	1.43	1.45
orf19.7149		1.37	0.85	0.90
orf19.7150	NRG1	1.33	1.53	1.24
orf19.7152		1.53	0.98	1.27
orf19.7153		1.24	1.19	1.07
orf19.7154		0.72	0.72	0.58
orf19.7160		0.81	1.22	0.66
orf19.7166		0.55	1.85	0.53
orf19.7170		1.14	0.60	0.98
orf19.7175	HLJ1	2.63	1.37	1.97
orf19.7176	NPT1	1.31	1.18	1.88
orf19.7177	KAP120	1.00	1.03	0.88
orf19.7178	PRE5	2.31	2.62	1.71
orf19.7179		2.90	2.02	1.53
orf19.7184		2.97	1.16	1.88
orf19.7185		1.81	1.00	1.07
orf19.7187	MAM33	0.99	0.70	0.94
orf19.719		1.31	1.65	1.53
orf19.7193		1.69	0.85	1.05
orf19.7195	RAD6	1.82	1.06	1.36
orf19.7197		0.70	1.06	0.41
orf19.7198		1.37	1.54	0.99
orf19.7199		1.66	1.03	1.29
orf19.7200		4.08	1.54	1.81
orf19.7202		1.67	0.80	1.23
orf19.7203		1.40	1.14	1.34
orf19.7216		2.13	1.11	1.79
orf19.7217	RPL4B	1.08	0.68	1.04
orf19.7219	FTR1	1.04	0.49	1.06
orf19.7228		2.79	1.48	3.48
orf19.7233		3.54	0.89	1.84
orf19.7236	TIF35	1.28	1.00	1.03
orf19.7238	NPL3	1.09	0.75	0.76
orf19.7242	NCR1	3.50	1.25	2.02
orf19.7243		2.50	0.88	1.54
orf19.7244		2.01	2.30	1.50
orf19.7250		2.74	2.86	2.15
orf19.7252		2.31	1.69	2.57
orf19.7255	RPC10	0.87	0.72	0.75
orf19.7256		1.72	0.94	1.47
orf19.7260		2.39	1.60	2.04
orf19.7264		1.48	1.11	1.09
orf19.7266		1.78	1.46	1.50
orf19.7269		1.46	0.82	1.10
orf19.7270		1.25	1.51	1.10
orf19.7272		3.29	1.60	1.66
orf19.7278		1.84	2.13	1.28
orf19.7297		1.57	1.29	1.02
orf19.7298	CHS2	0.90	0.66	1.55
orf19.73		3.06	1.20	1.84
orf19.7308	TUB1	1.36	0.49	0.89
orf19.731	EMP46	1.57	1.74	1.42
orf19.7312	ERG13	1.33	0.72	0.91
orf19.7322		1.18	1.20	1.12
orf19.7325		0.85	0.56	0.57
orf19.7326		1.56	2.16	0.89
orf19.7327	PHO88	1.60	0.59	4.01
orf19.7329		1.42	0.96	0.94
orf19.7332	ELF1	0.51	1.25	0.43

orf19.7335	PRE8	1.42	0.93	1.01
orf19.7346		1.03	0.73	1.25
orf19.7349	CHS4	1.30	1.85	1.09
orf19.735		1.74	1.08	1.28
orf19.7354		1.17	0.42	0.93
orf19.7358		1.19	1.49	1.16
orf19.7363	KRE6	1.60	0.65	1.02
orf19.7366		1.20	0.95	1.02
orf19.7367	UBP1	1.06	1.20	0.89
orf19.7368		1.42	0.66	1.24
orf19.7378		2.20	1.38	1.37
orf19.738	MYO5	2.25	2.67	2.03
orf19.7383	MNN9	2.24	0.71	1.55
orf19.7384	NOG1	0.75	0.93	0.65
orf19.7386		1.10	1.25	1.43
orf19.7387		1.05	0.96	0.87
orf19.7388	PBS2	1.97	0.85	1.64
orf19.7391	OCH1	1.50	0.77	1.12
orf19.7392		1.45	0.89	0.99
orf19.7394	GDA1	1.46	0.99	1.45
orf19.740	HAP41	1.43	0.57	0.92
orf19.7404		1.10	1.47	1.22
orf19.7409	ERV25	1.53	0.65	1.37
orf19.7420		2.06	2.81	1.35
orf19.7421	CYP5	1.59	0.95	1.45
orf19.7422		0.68	0.80	0.51
orf19.7427		1.00	0.63	1.05
orf19.7433		1.07	1.00	0.87
orf19.7436	AAF1	1.12	1.55	1.00
orf19.7437		1.99	2.42	1.46
orf19.744	GDB1	1.25	1.28	1.13
orf19.7442		1.74	1.17	1.89
orf19.7447	JEN1	1.39	1.00	1.16
orf19.7448	LYS9	1.56	0.83	1.01
orf19.7450		2.63	2.35	1.61
orf19.7454	TAF60	1.84	1.01	1.49
orf19.7466	ACC1	0.65	0.53	0.56
orf19.7475	PHO81	1.15	1.31	1.68
orf19.7477	YRB1	1.17	1.01	1.09
orf19.7479	NTH1	1.70	2.16	0.93
orf19.748		1.06	0.87	0.95
orf19.7483	CRM1	1.67	1.24	1.33
orf19.7484	ADE1	1.28	0.60	1.00
orf19.7485		1.04	0.73	0.93
orf19.7486	MRPL6	1.76	1.67	1.51
orf19.7488		0.87	1.07	0.82
orf19.7489		0.98	0.58	1.20
orf19.7489.3		1.05	0.63	1.07
orf19.7509.1	ATP17	1.35	0.89	1.51
orf19.7509.2	LSM6	1.19	0.75	1.08
orf19.7511		1.21	0.75	1.08
orf19.7520	POT1	2.72	0.74	1.16
orf19.7523	MKC1	1.71	1.08	1.34
orf19.753		2.78	1.13	2.66
orf19.7534		0.72	0.62	0.49
orf19.7538		1.99	0.96	1.40
orf19.754		0.98	0.74	0.78
orf19.7549	PMT5	1.91	1.22	1.56
orf19.755	MRPL37	1.33	0.88	1.21
orf19.7551	ALO1	1.31	1.29	0.99
orf19.7552		0.96	1.51	0.57
orf19.7563	BET2	2.29	1.38	1.92
orf19.7566		1.35	0.44	0.84
orf19.7569		0.90	0.63	0.62
orf19.7571	UBC4	1.84	1.44	1.62
orf19.7572	SPT7	1.96	1.15	1.37
orf19.7577	MSS51	1.27	0.97	1.16

orf19.7579	FGR34	2.44	1.25	1.39
orf19.7580		0.92	0.85	0.96
orf19.7581		1.54	1.10	0.91
orf19.7586	CHT3	0.61	0.40	0.69
orf19.7590		2.12	0.89	1.35
orf19.7591		2.02	1.20	1.37
orf19.7592	FAA4	0.92	0.74	0.60
orf19.7593		1.17	1.23	1.02
orf19.7596		3.89	1.86	2.94
orf19.7599		0.75	0.95	0.63
orf19.76	SPB1	0.61	1.09	0.49
orf19.760		1.68	1.40	1.42
orf19.7602		1.80	1.50	1.29
orf19.7604		2.37	1.74	1.78
orf19.7613	HCR1	1.25	1.15	1.02
orf19.7615		2.90	1.54	1.79
orf19.7617		1.40	1.11	1.14
orf19.7618		0.87	0.70	0.57
orf19.7624		1.43	1.50	1.02
orf19.7626	EIF4E	1.32	0.89	1.28
orf19.763		1.28	1.43	1.07
orf19.7631		3.80	1.17	2.31
orf19.7638	PRO1	1.20	1.55	1.81
orf19.7643		1.66	1.12	1.44
orf19.7648		5.89	1.37	2.39
orf19.7650	LTV1	0.69	0.79	0.69
orf19.7652	CKA1	1.86	1.82	1.25
orf19.7655	RPO21	1.39	0.74	1.10
orf19.7658	RFC4	2.06	1.56	1.50
orf19.7661	HMI1	2.00	1.14	1.98
orf19.7662		1.85	1.33	1.18
orf19.7663		1.64	1.58	1.51
orf19.7664		1.13	1.08	1.32
orf19.7668	MAL2	4.09	0.84	1.79
orf19.7670		1.55	0.66	0.92
orf19.7672		2.07	0.93	1.77
orf19.7673		1.22	0.77	1.29
orf19.7675		1.22	0.65	0.97
orf19.7676	XYL2	0.88	0.88	0.83
orf19.7678		1.80	1.05	1.76
orf19.772	LYS21	1.36	0.84	1.03
orf19.773		1.13	0.70	0.55
orf19.775		2.23	1.58	1.74
orf19.776		2.82	1.75	1.34
orf19.778	PIL1	1.55	0.80	0.95
orf19.779		2.14	0.97	1.26
orf19.785		1.44	0.77	1.40
orf19.789	PYC2	1.41	0.67	0.86
orf19.791	RIM11	2.18	1.39	2.24
orf19.793	CAK1	1.06	0.52	0.76
orf19.797	BAT21	1.15	1.90	0.97
orf19.798	TAF14	1.44	1.17	1.28
orf19.801	TBF1	1.01	0.59	0.78
orf19.807	CHS5	2.45	4.09	1.88
orf19.809		0.56	0.89	0.48
orf19.813		0.67	0.59	0.32
orf19.814	SSY1	2.08	2.92	2.08
orf19.815	DCK1	1.95	1.12	1.35
orf19.819		1.54	1.03	1.57
orf19.820	SDS22	1.59	2.77	1.50
orf19.825	GCD7	1.12	1.33	0.97
orf19.827.1	RPL39	1.16	0.72	1.03
orf19.828		1.35	0.69	1.07
orf19.829	SCH9	0.92	0.80	0.87
orf19.83		1.10	1.18	1.43
orf19.832	GPI13	1.20	0.86	1.12
orf19.838.1	RPS9B	1.12	0.68	1.07

orf19.839		0.85	0.64	0.76
orf19.840	RPL21A	1.11	0.77	1.07
orf19.842	ASR3	1.71	0.69	1.03
orf19.863		1.17	0.84	0.86
orf19.873.1	COX6	1.25	0.77	1.07
orf19.876	PGA33	2.19	0.76	1.51
orf19.879		1.96	1.14	1.32
orf19.880		1.08	1.06	0.90
orf19.882	HSP78	2.84	10.70	3.74
orf19.886	PAN1	1.93	1.59	1.68
orf19.889	THI20	1.76	2.61	2.32
orf19.891		1.45	1.14	0.82
orf19.895	HOG1	1.73	1.57	1.42
orf19.898	HEM2	1.38	1.04	1.32
orf19.90		2.35	1.81	1.91
orf19.900		1.38	1.01	0.97
orf19.906	ROM2	1.36	1.29	1.07
orf19.907		2.05	0.63	1.77
orf19.908	FEN12	1.31	0.90	1.28
orf19.912		1.35	1.03	0.85
orf19.913.2		1.40	0.92	1.42
orf19.915		1.59	2.67	1.28
orf19.917		1.90	1.38	1.29
orf19.918	CDR11	2.27	0.97	1.46
orf19.919		3.26	1.28	2.25
orf19.92		2.13	1.16	1.48
orf19.920	RMT2	1.03	0.98	0.80
orf19.922	ERG11	0.82	0.53	0.91
orf19.923	THR1	1.37	1.14	1.45
orf19.924		1.59	1.31	1.45
orf19.926		2.17	1.31	1.31
orf19.927		1.83	0.99	1.54
orf19.93		2.05	0.88	1.39
orf19.930	PET9	1.78	0.64	1.33
orf19.939	NAM7	0.88	1.20	1.03
orf19.941	SEC14	1.21	0.87	1.13
orf19.943	FET33	1.94	0.72	1.30
orf19.946	MET14	1.17	1.15	1.13
orf19.947	MRP17	1.31	1.42	1.23
orf19.952		7.94	0.97	1.85
orf19.953.1	COF1	1.41	0.85	1.12
orf19.955		1.80	1.01	1.41
orf19.96	TOP1	0.76	0.59	0.65
orf19.961.2		1.27	0.92	1.05
orf19.962		0.82	0.75	0.64
orf19.964		2.14	1.00	0.97
orf19.967		1.59	0.85	0.97
orf19.968	PGA14	1.45	0.82	1.17
orf19.969		1.05	0.61	0.74
orf19.976	BRE1	1.50	1.17	1.19
orf19.978	BDF1	0.81	0.92	0.72
orf19.980		1.07	0.94	1.10
orf19.989		1.38	0.83	0.91
orf19.992	LKH1	1.50	1.37	1.38
orf19.993		2.48	2.03	1.60
orf19.998		1.93	1.40	1.28
