# Supplementary material for: Mechanisms Underlying the Exquisite Sensitivity of Candida albicans to Combinatorial Cationic and Oxidative Stress That Enhances the Potent Fungicidal Activity of Phagocytes
Source: mBio. 2014 Jul 15;5(4):e01334-14. doi: 10.1128/mBio.01334-14 (PMC4161263; doi:10.1128/mBio.01334-14)
Supplement: Table S3 — qRT-PCR primers and probes. [file mbo004141905st3.pdf]

Table S3. qRT-PCR primers and probes used

Gene	Probe	Forward Primer	Reverse Primer
ACT1	9	aaccaccggtattgttttgg	gcgtaaattggaacaacgtg
HGT10	22	accaatttcgccgttgttat	acaaccccaaccactagcag
TRR1	77	ttcagaaacaacccattagctg	aaatggcttcttcacaagctg
CAT1	78	tggttttattctccgacagagg	tgaccagagtaaccattcatttct
